# Supplementary material for: New Derivatives of 5-Substituted Uracils: Potential Agents with a Wide Spectrum of Biological Activity
Source: Molecules. 2022 Apr 30;27(9):2866. doi: 10.3390/molecules27092866 (PMC9102953; doi:10.3390/molecules27092866)
Supplement: Supplementary file 1 [file molecules-27-02866-s001.zip › molecules-1674577-supplementary.pdf]

Supplementary Material

New derivatives of 5-substituted uracils: potential agents with a wide spectrum of biological activity

Vasily A. Kezin[1], Elena S. Matyugina[1], Mikhail S. Novikov[2], Alexander O. Chizhov[3], Robert Snoeck[4], Graciela Andrei[4], Sergei N. Kochetkov[1], Anastasiya L. Khandazhinskaya[1*]

[1] *Engelhardt Institute of Molecular Biology, Russian Academy of Science, Moscow, 119991 Russia. vassilevs58@yandex.ru (V.A.K); matyugina@gmail.com (E.S.M.); kochet@eimb.ru (S.N.K.); khandazhinskaya@bk.ru (A.L.K).*

[2] *Department of Pharmaceutical & Toxicological Chemistry, Volgograd State Medical University, Volgograd, 400131 Russia. m-novikov1@mail.ru (M.S.N.)*

[3] *N.D. Zelinsky Institute of Organic Chemistry RAS, Leninski pr. 47, Moscow 119991, Russia. chizhov@ioc.ac.ru (A.O.C.)*

[4] *Rega Institute for Medical Research, KU Leuven, B-3000 Leuven, Belgium. robert.snoeck@kuleuven.be (R.S.), graciela.andrei@rega.kuleuven.be (G.A.)*

**Correspondence: khandazhinskaya@bk.ru; Tel.: +7 (499) 135-60-65*

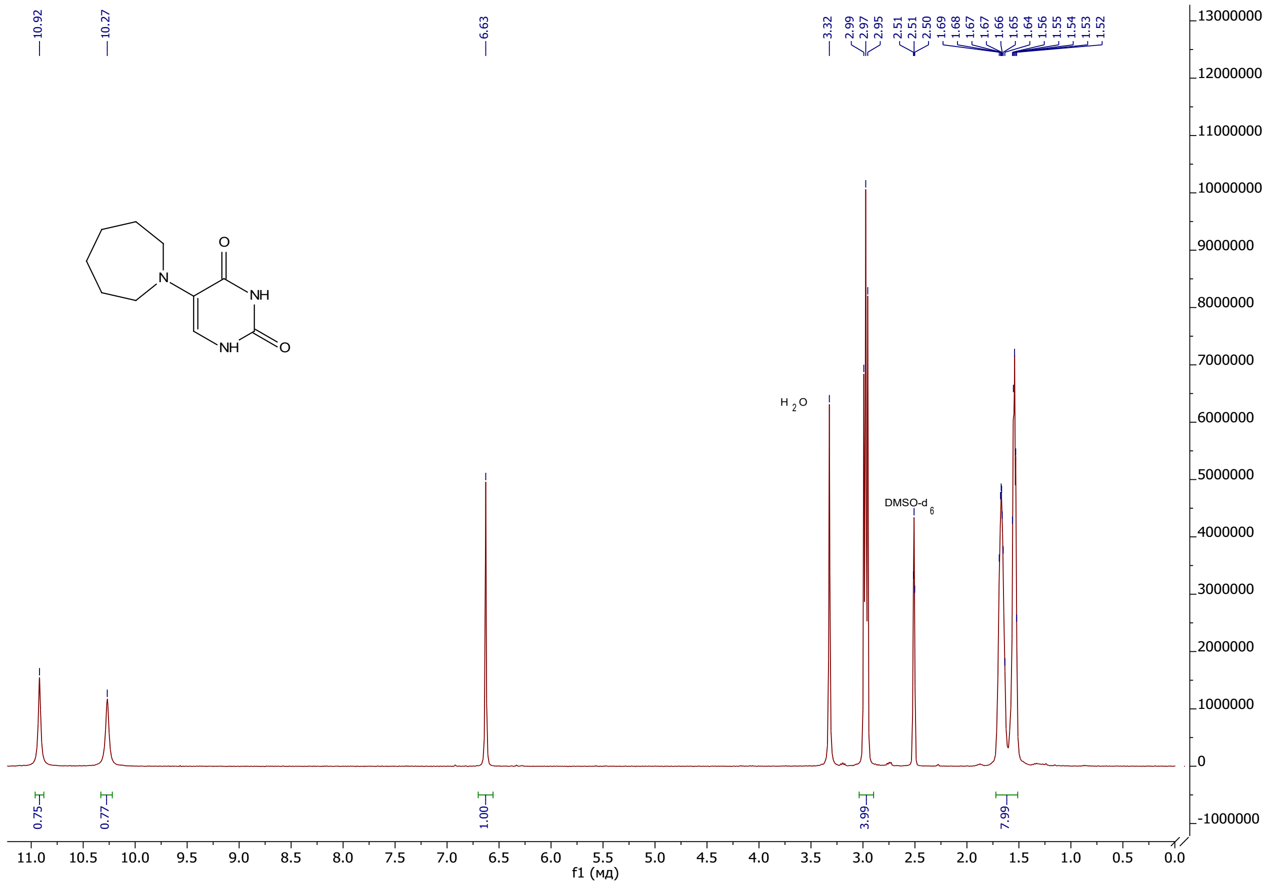

Figure S1 ^{1}H NMR spectrum of compound **2a** in DMSO-*d6*

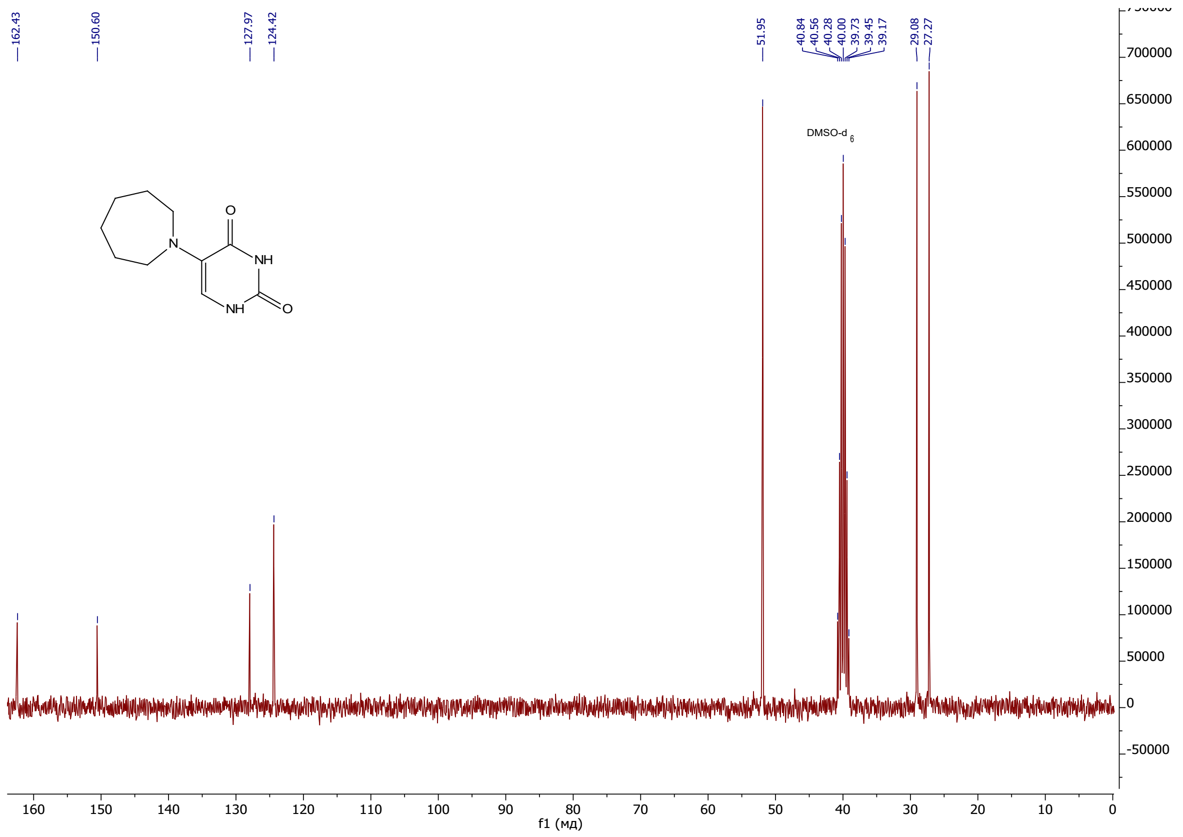

Figure S2 ^{13}C NMR spectrum of compound **2a** in DMSO-*d6*

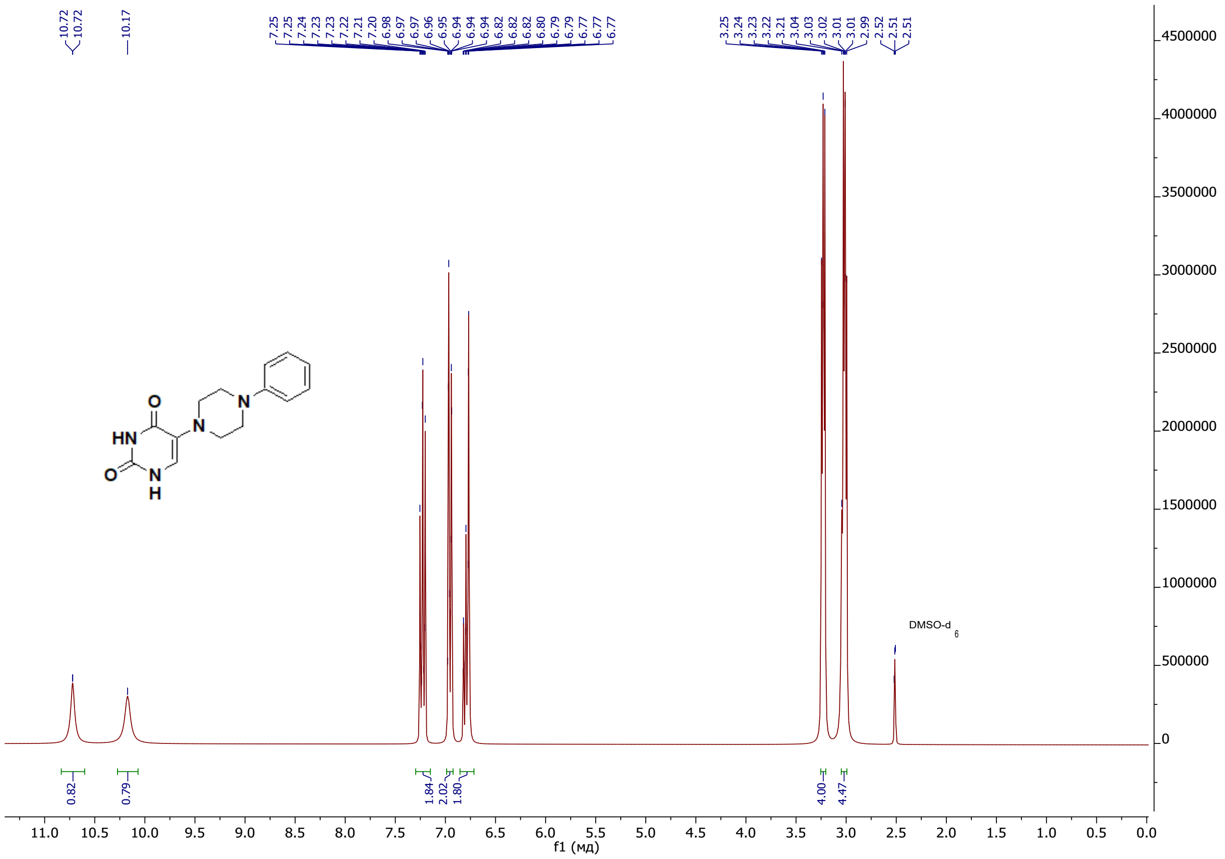

Figure S3 ^{1}H NMR spectrum of compound **2b** in DMSO-*d*6 at 400 MHz.

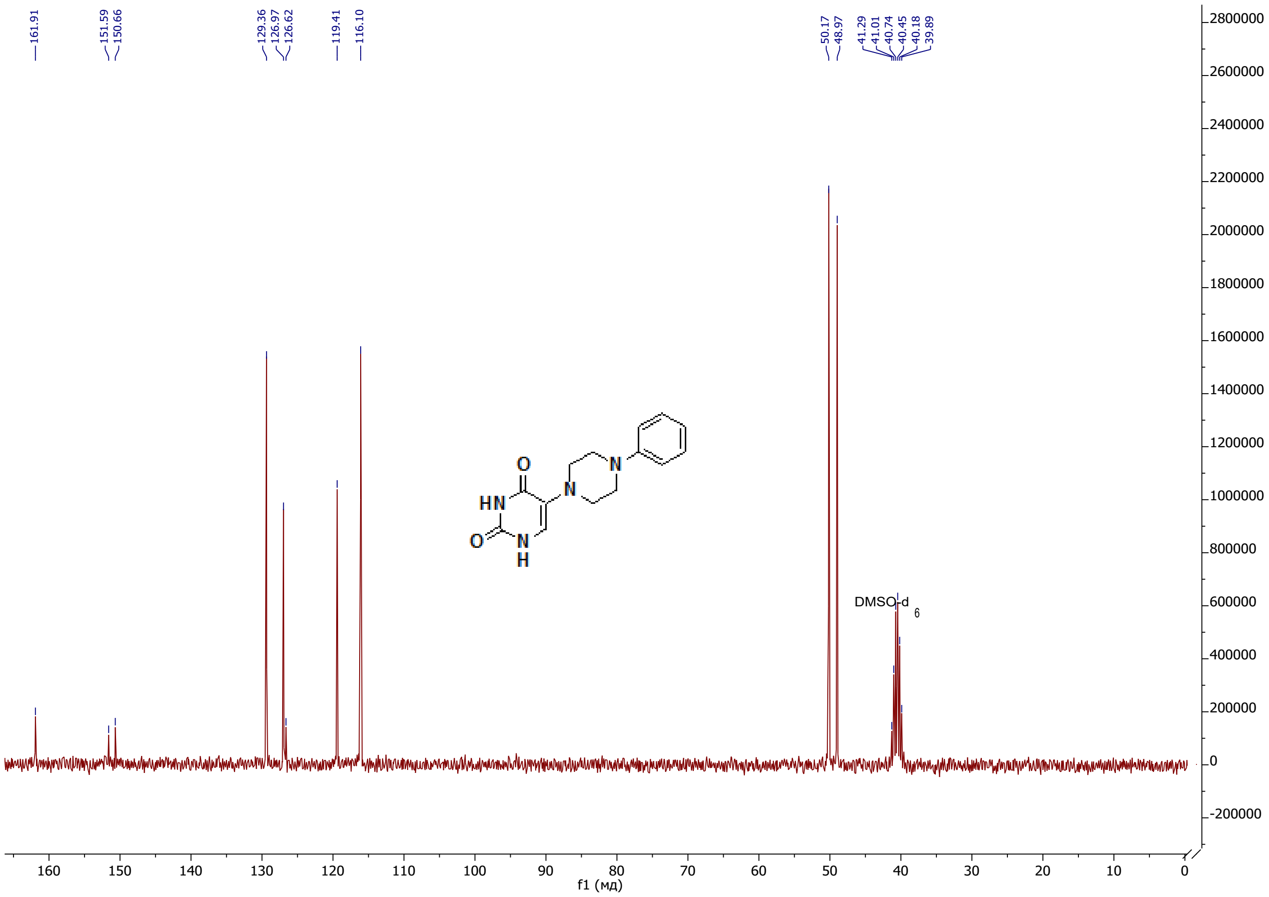

Figure S4 ^{13}C NMR spectrum of compound **2b** in DMSO-*d*6 at 100 MHz.

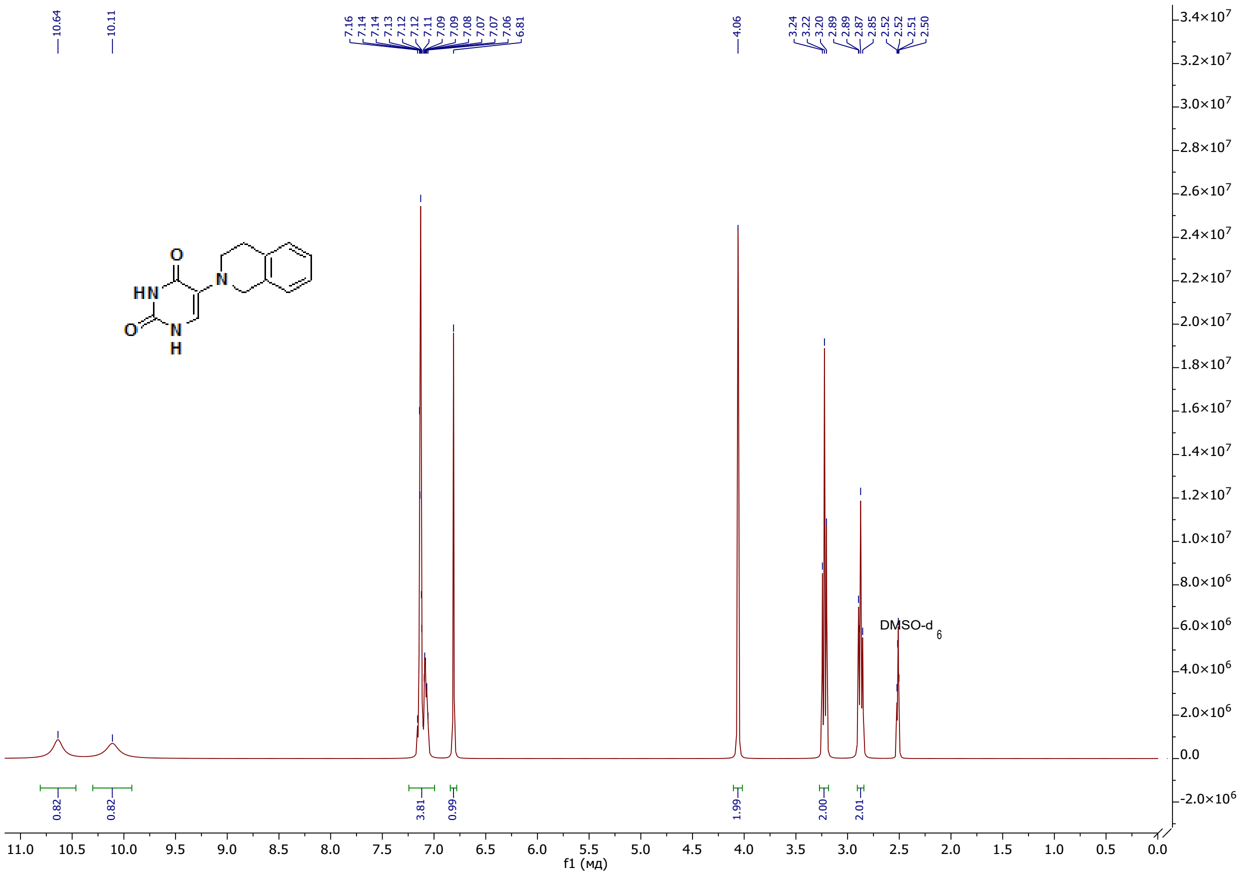

Figure S5 ^{1}H NMR spectrum of compound **2c** in DMSO-*d*6 at 400 MHz.

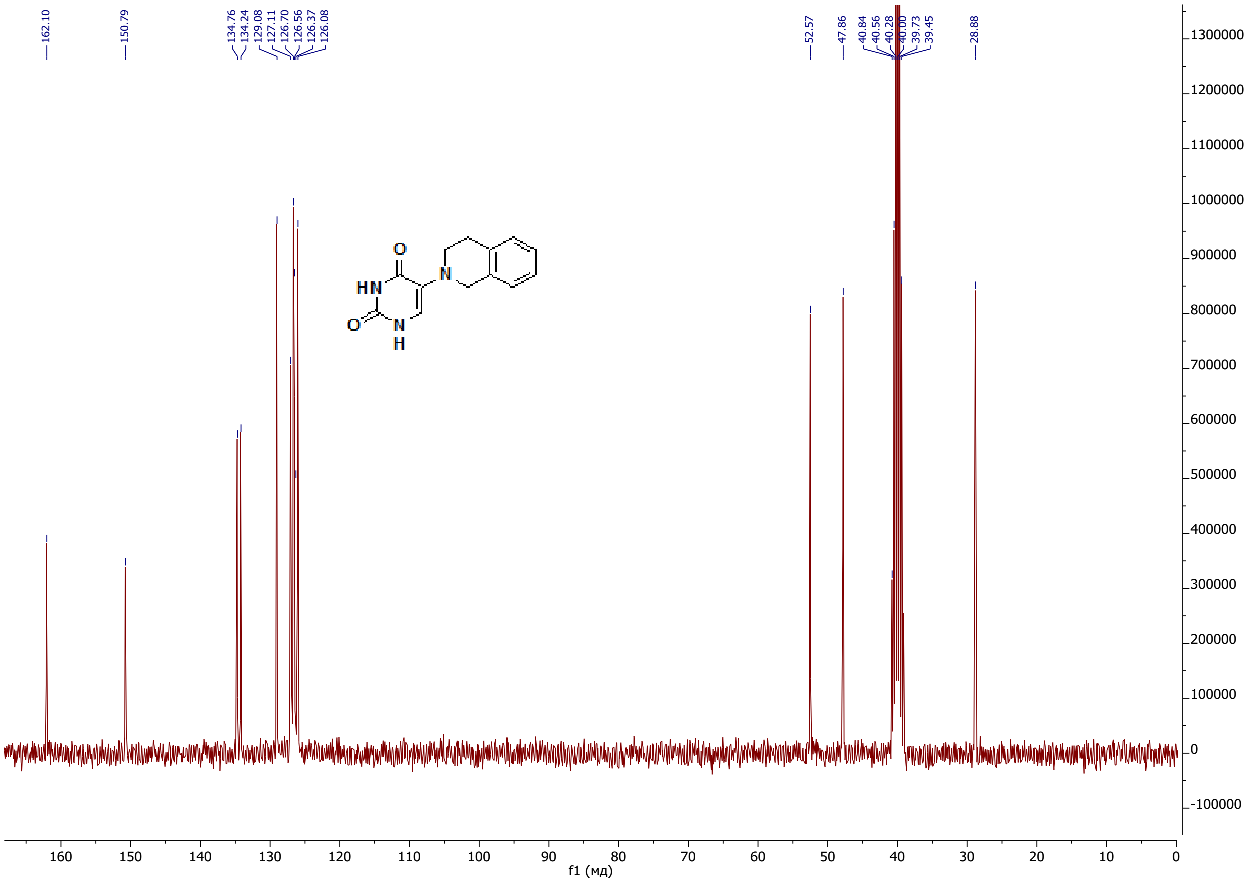

Figure S6 ^{13}C NMR spectrum of compound **2c** in DMSO-*d*6 at 100 MHz.

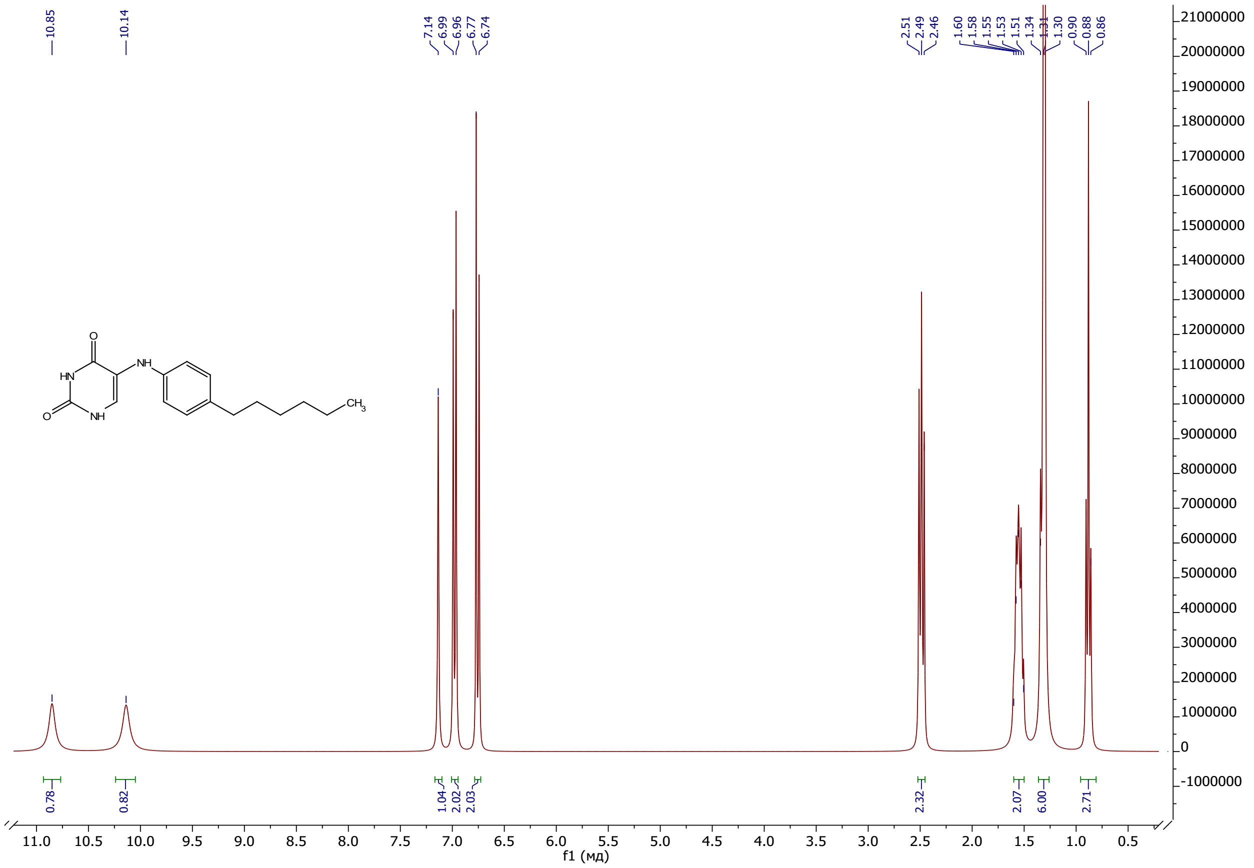

Figure S7 ^{1}H NMR spectrum of compound **2d** in DMSO-*d*6 at 400 MHz.

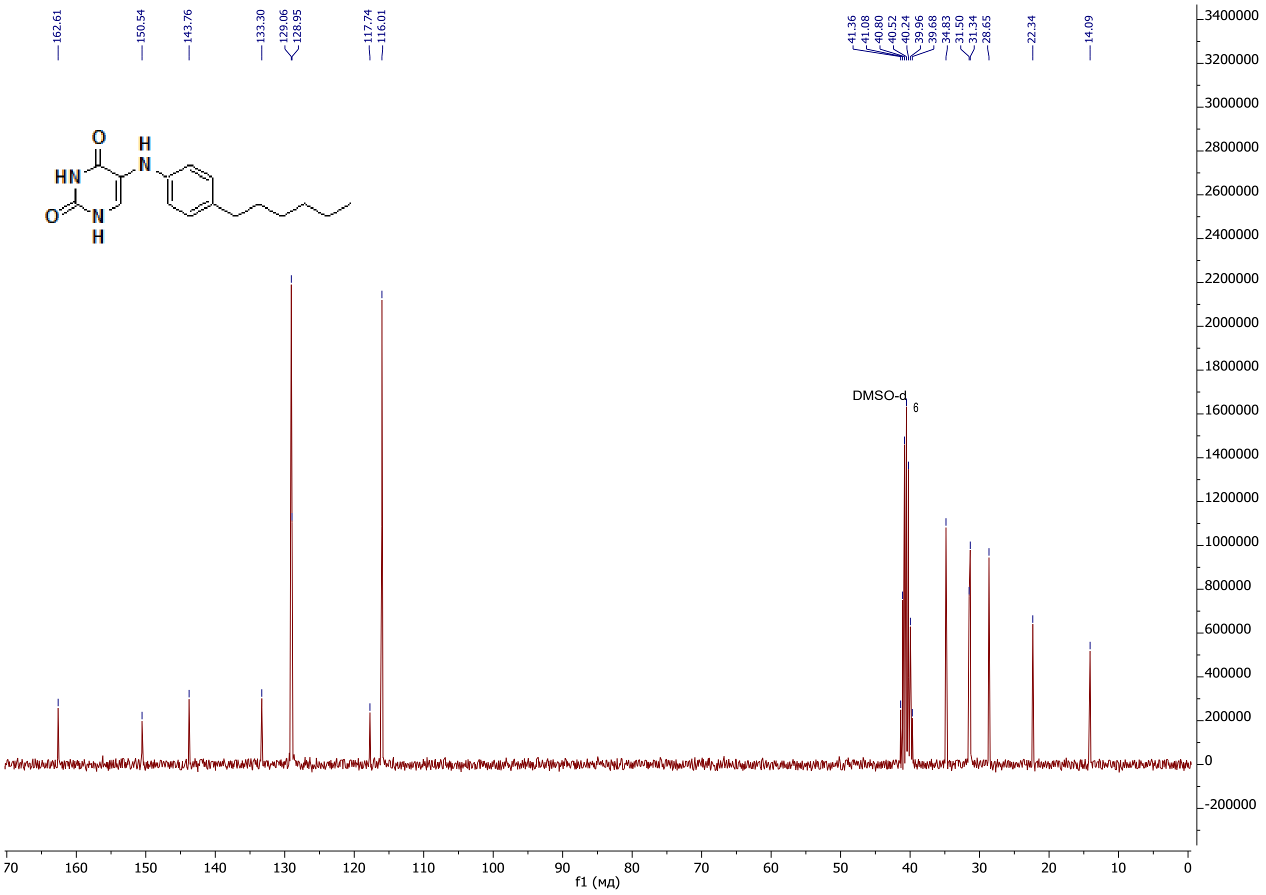

Figure S8 ^{13}C NMR spectrum of compound **2d** in DMSO-*d*6 at 100 MHz.

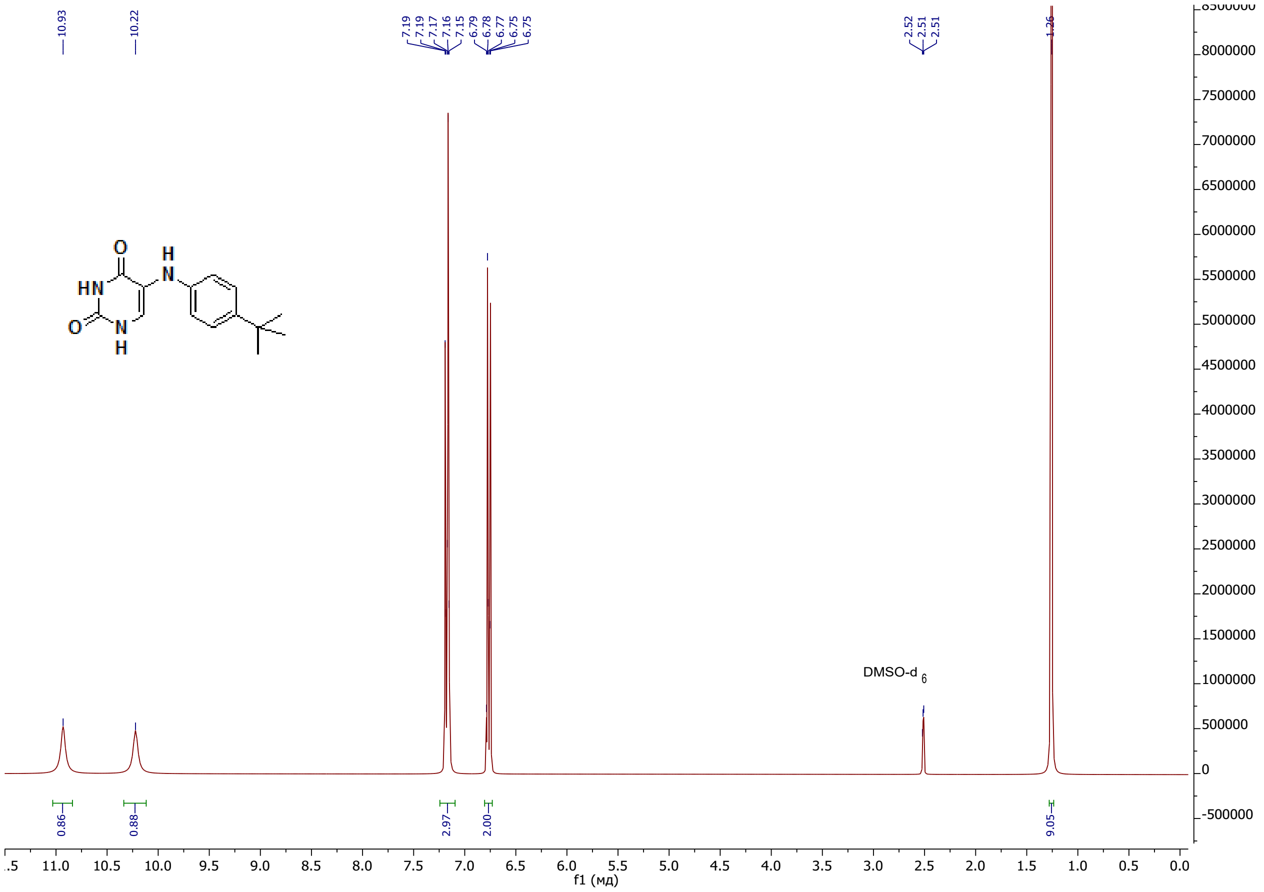

Figure S9 ^{1}H NMR spectrum of compound **2e** in DMSO-*d6* at 400 MHz.

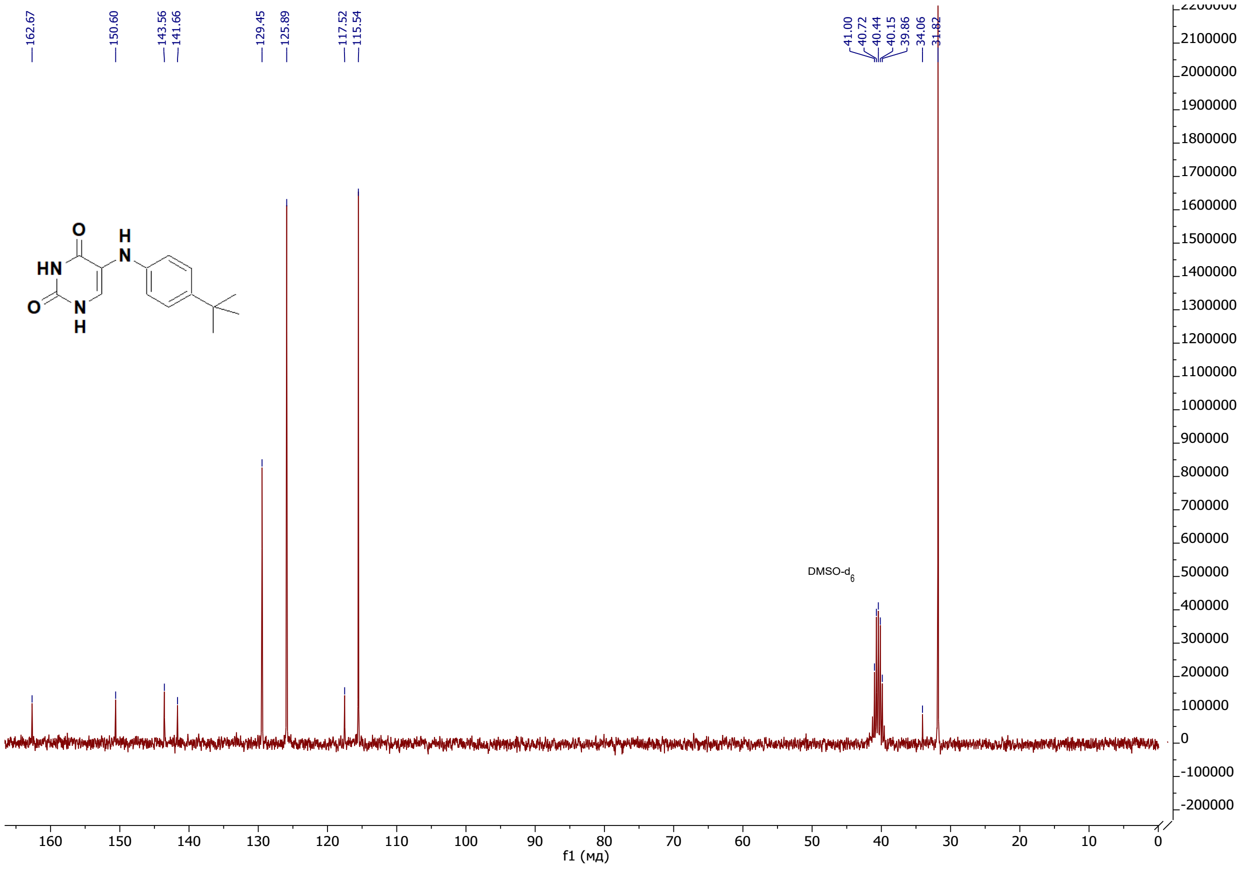

Figure S10 ^{13}C NMR spectrum of compound **2e** in DMSO-*d6* at 100 MHz.

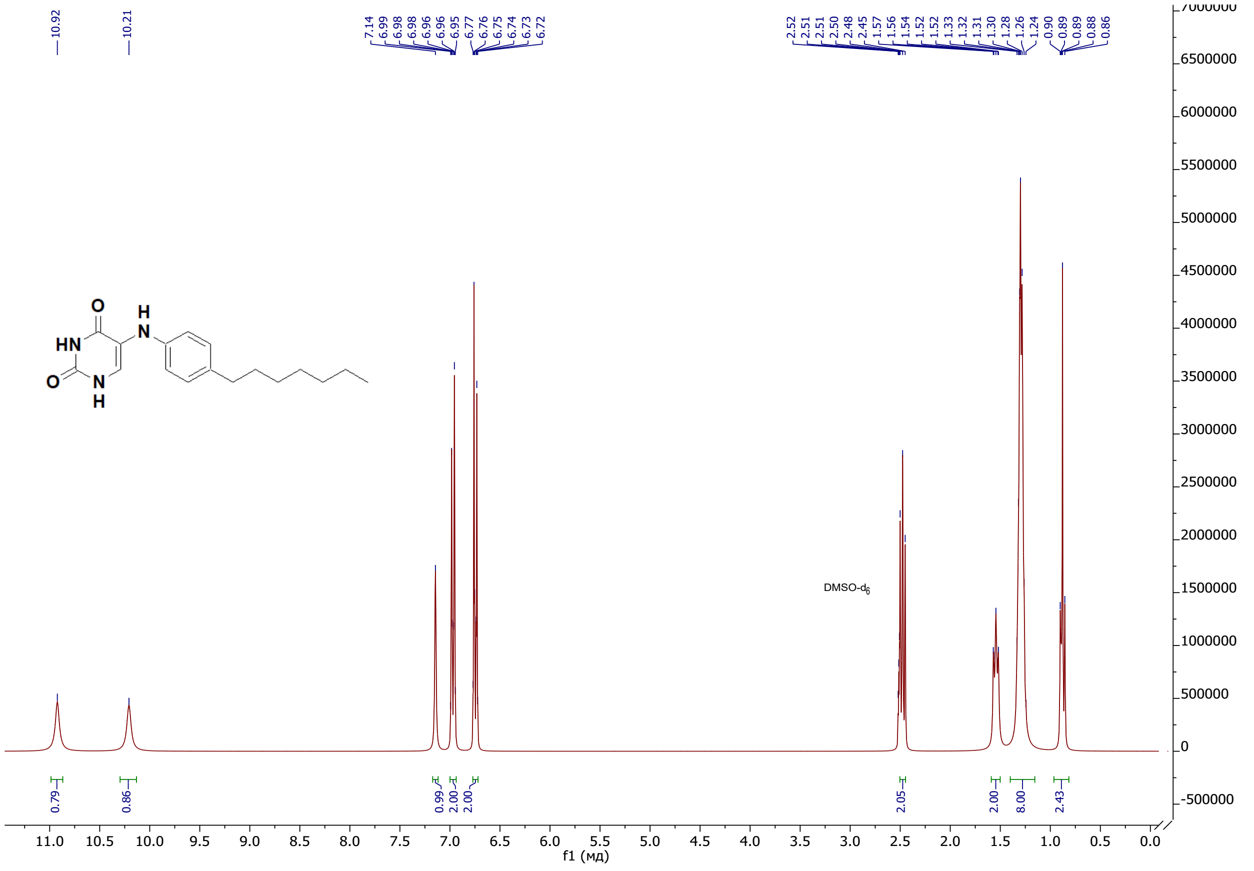

Figure S11 ^{1}H NMR spectrum of compound **2f** in DMSO-*d6* at 400 MHz.

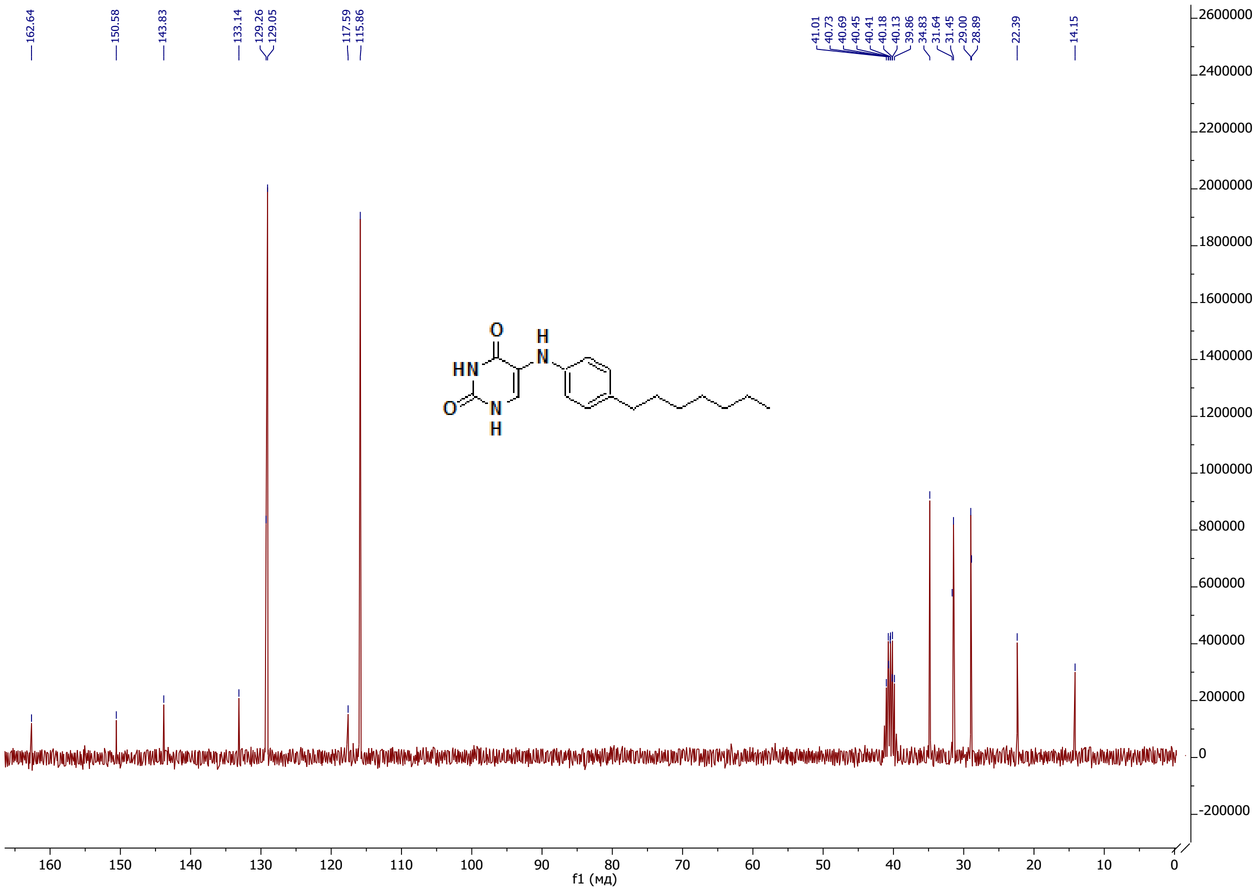

Figure S12 ^{13}C NMR spectrum of compound **2f** in DMSO-*d6* at 100 MHz.

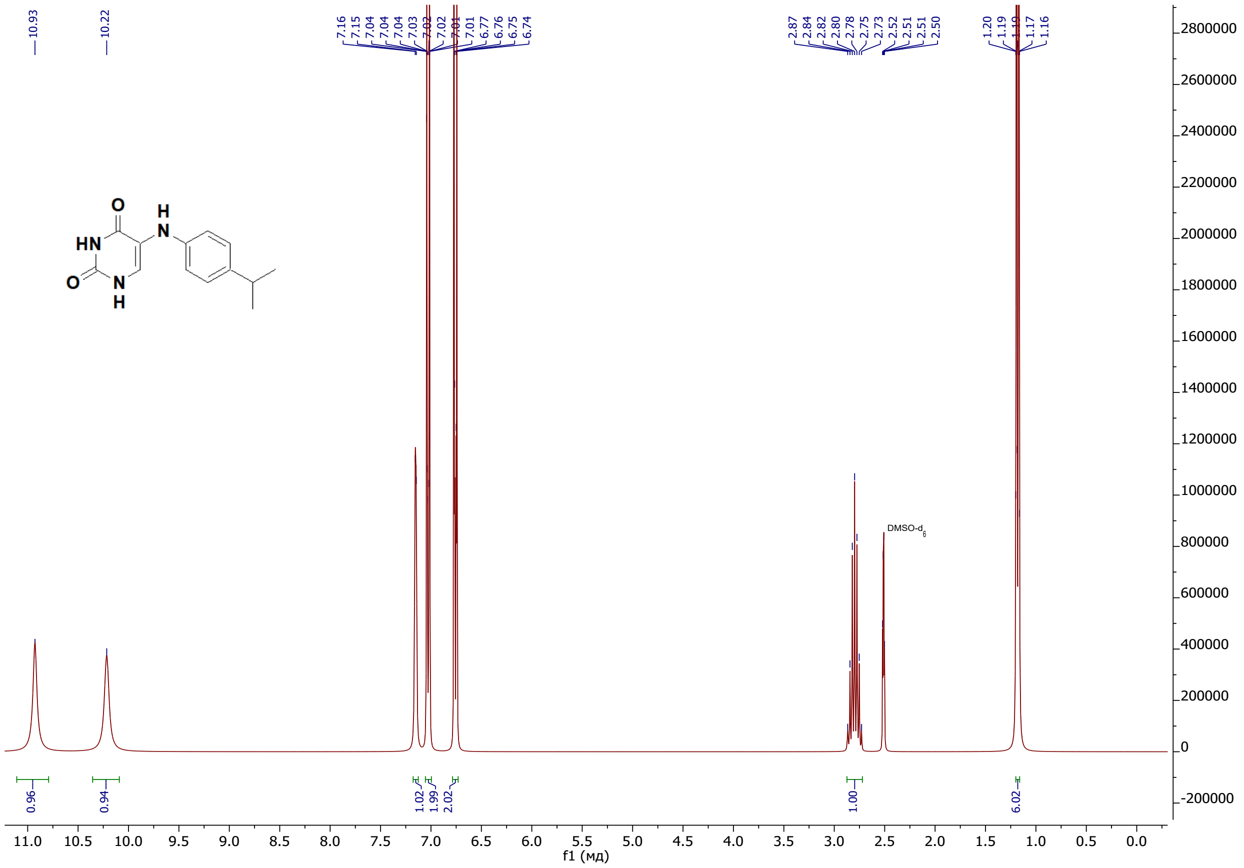

Figure S13 ^{1}H NMR spectrum of compound **2g** in DMSO-*d6* at 400 MHz.

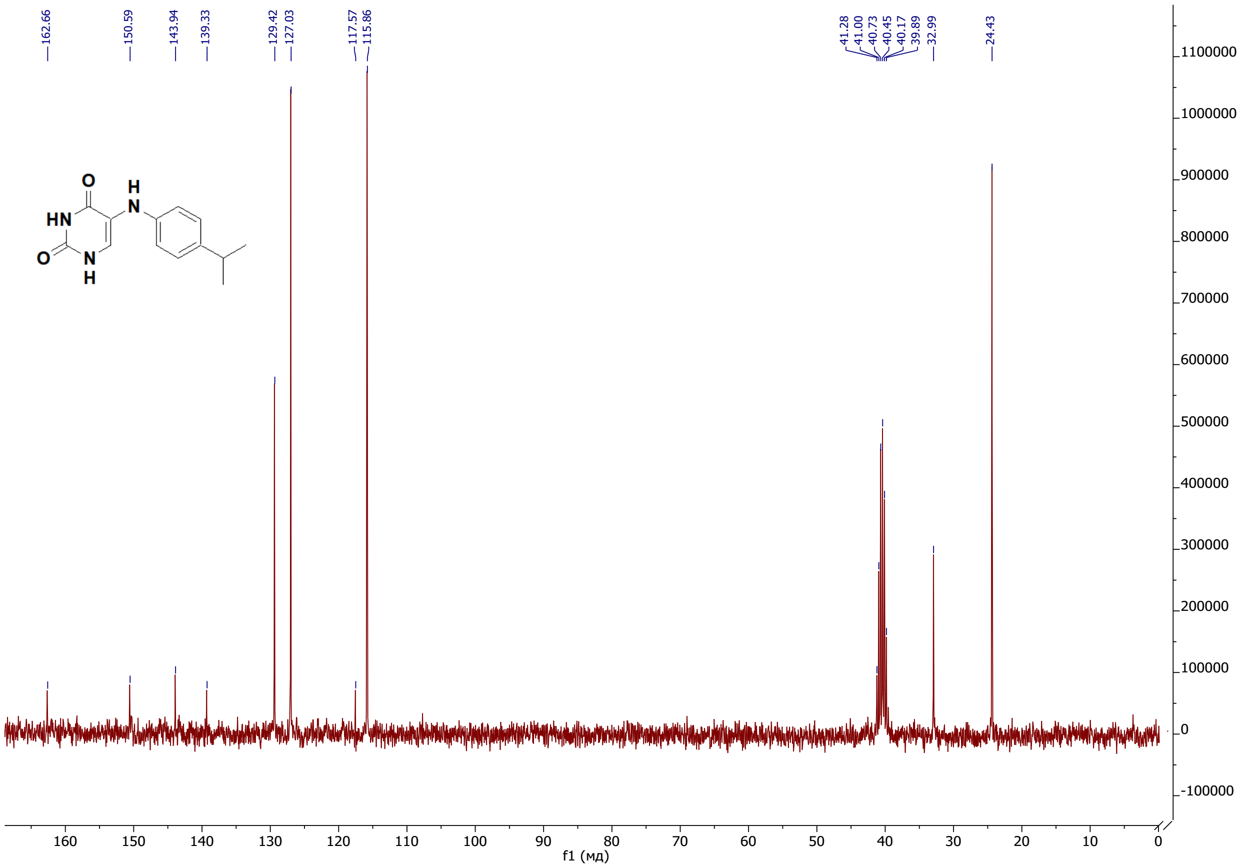

Figure S14 ^{13}C NMR spectrum of compound **2g** in DMSO-*d6* at 100 MHz.

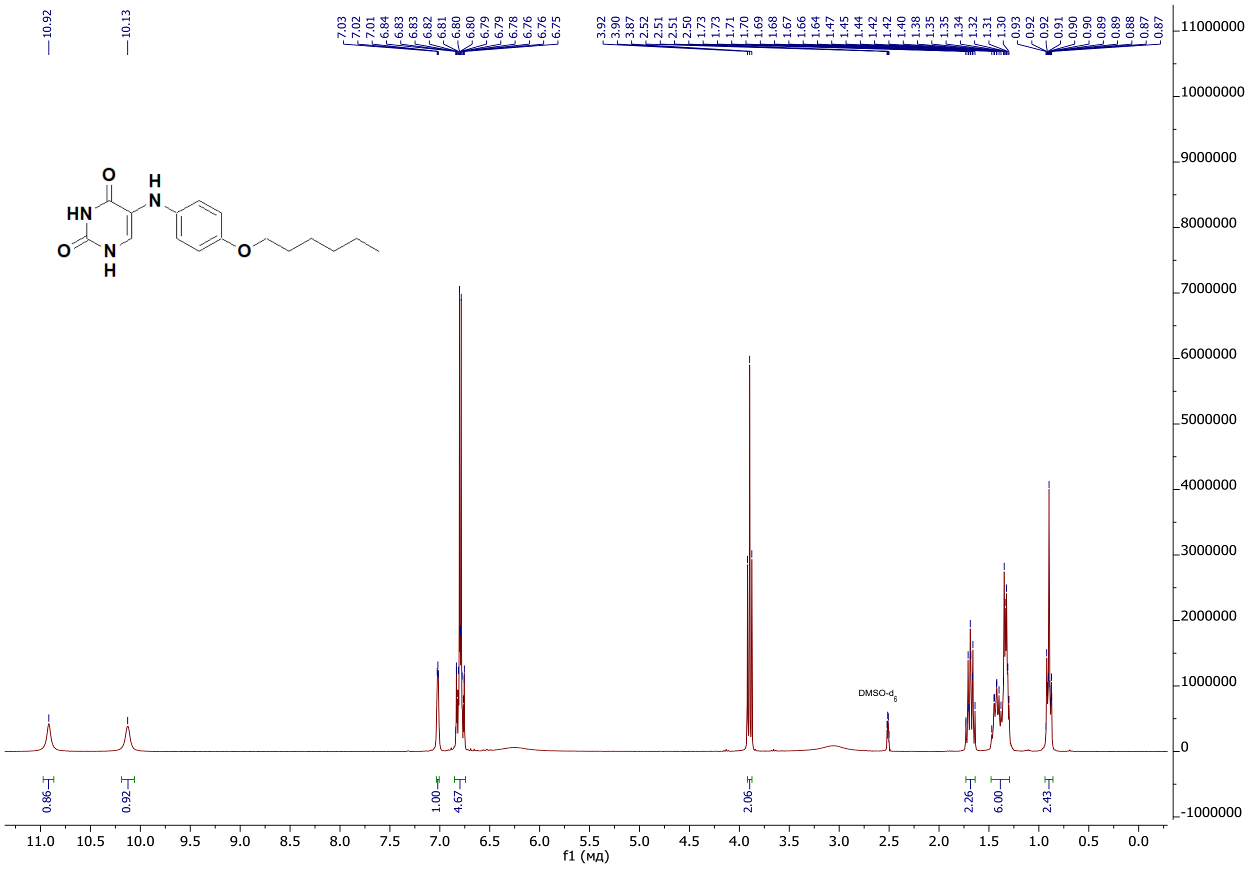

Figure S15 ^{1}H NMR spectrum of compound **2h** in DMSO-*d6* at 400 MHz.

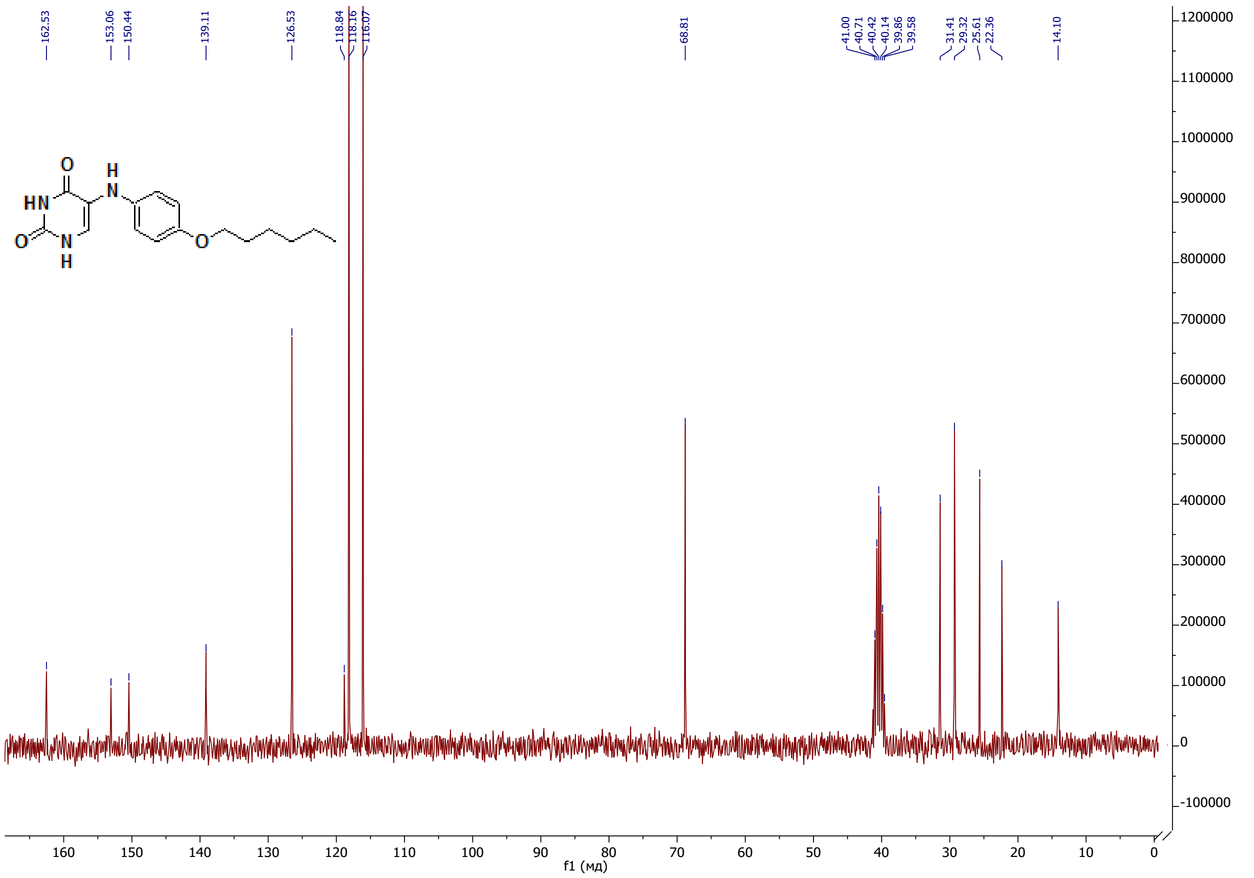

Figure S16 ^{13}C NMR spectrum of compound **2h** in DMSO-*d6* at 100 MHz.

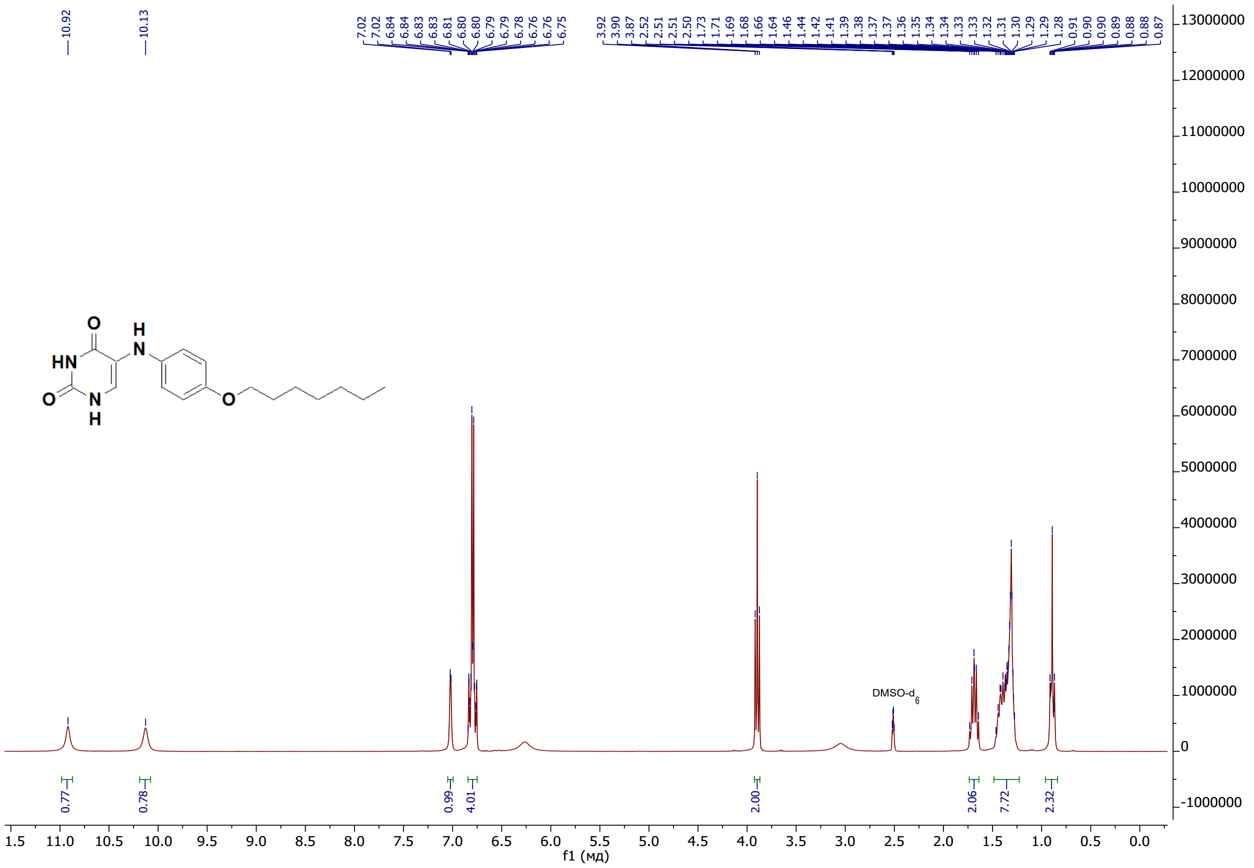

Figure S17 ^{1}H NMR spectrum of compound **2i** in DMSO-*d*6 at 400 MHz.

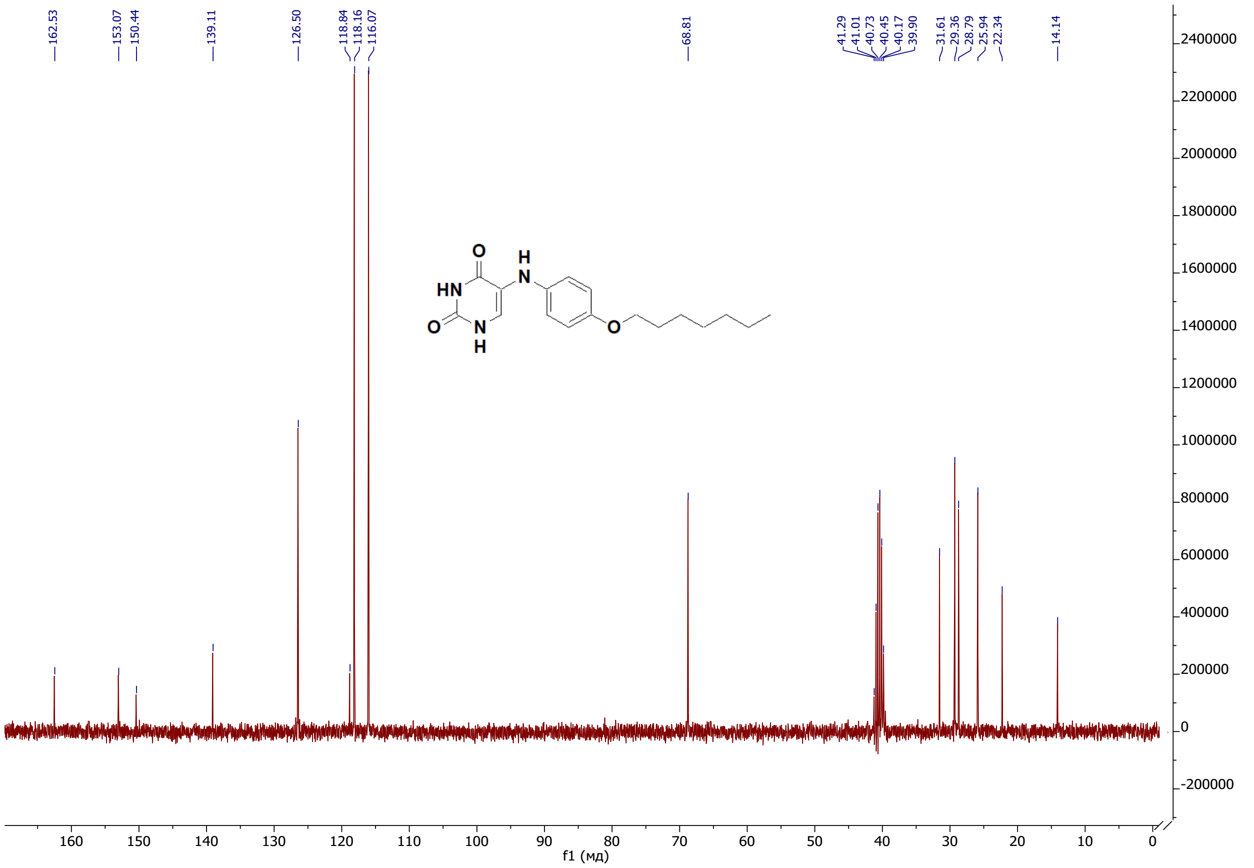

Figure S18 ^{13}C NMR spectrum of compound **2i** in DMSO-*d*6 at 100 MHz.

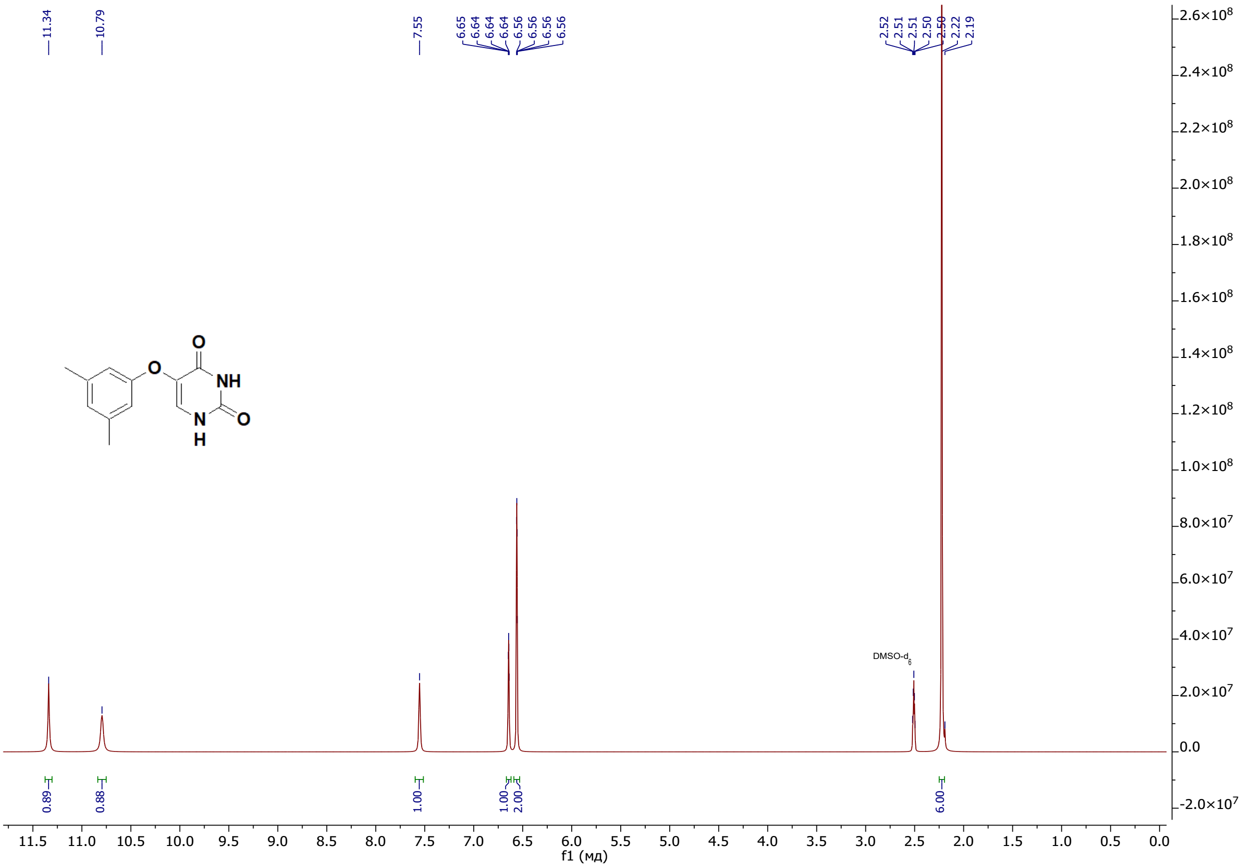

Figure S19 ^{1}H NMR spectrum of compound **5a** in DMSO-*d*6 at 400 MHz.

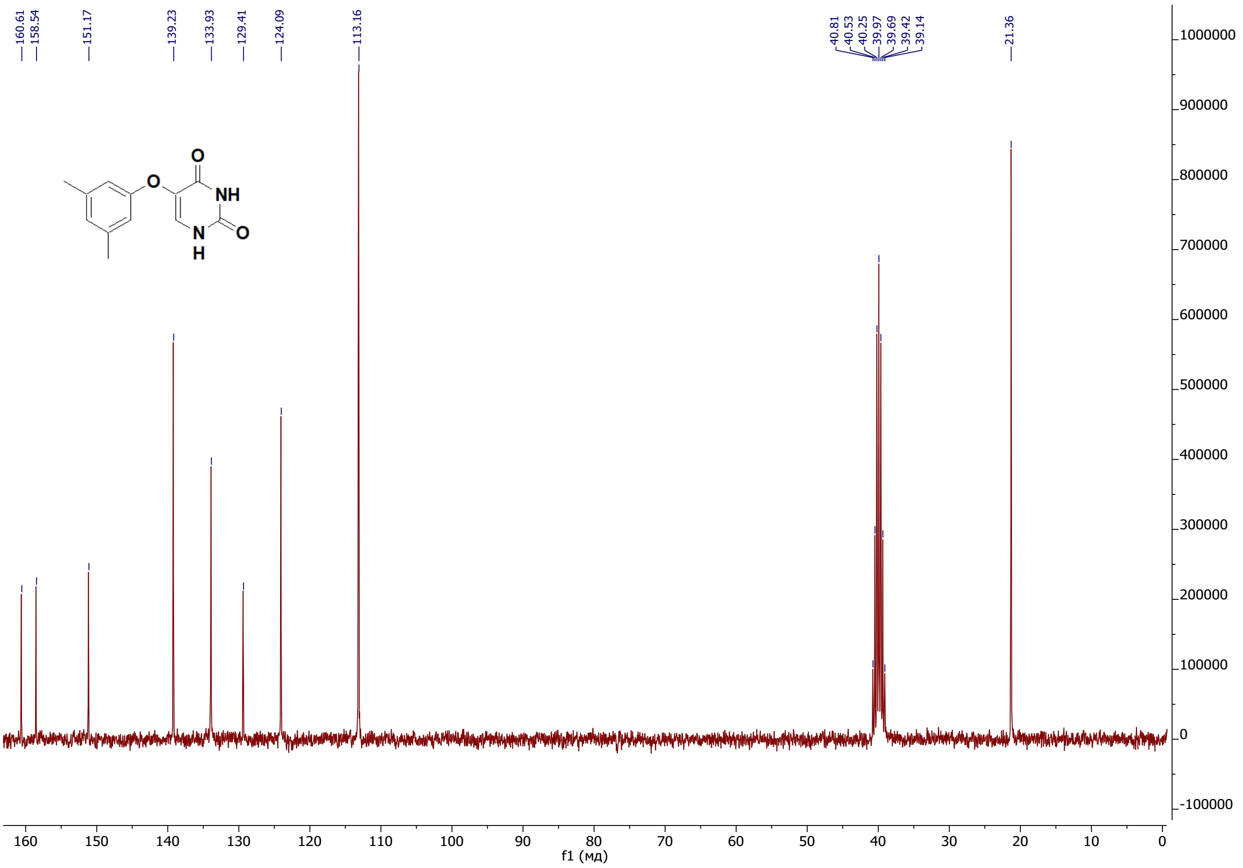

Figure S20 ^{13}C NMR spectrum of compound **5a** in DMSO-*d*6 at 100 MHz.

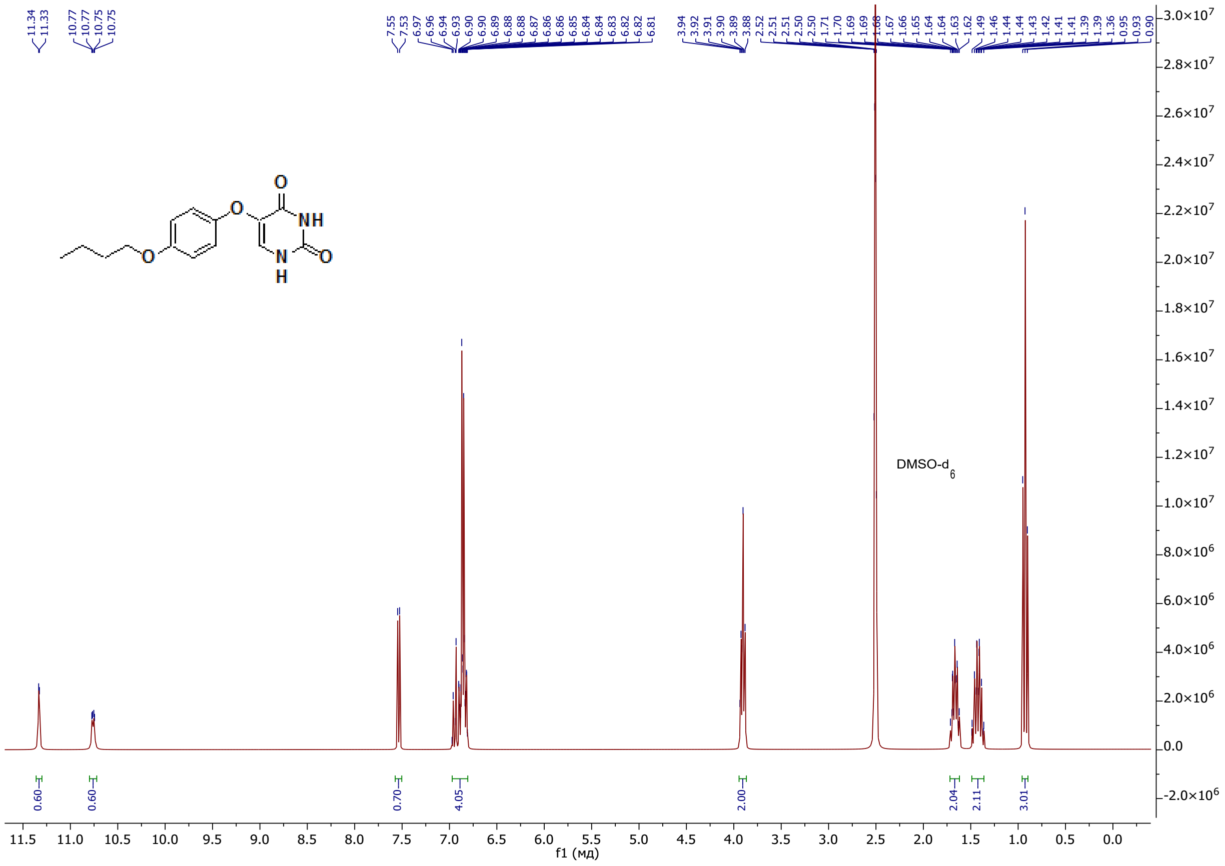

Figure S21 ^{1}H NMR spectrum of compound **5b** in DMSO-*d6* at 400 MHz.

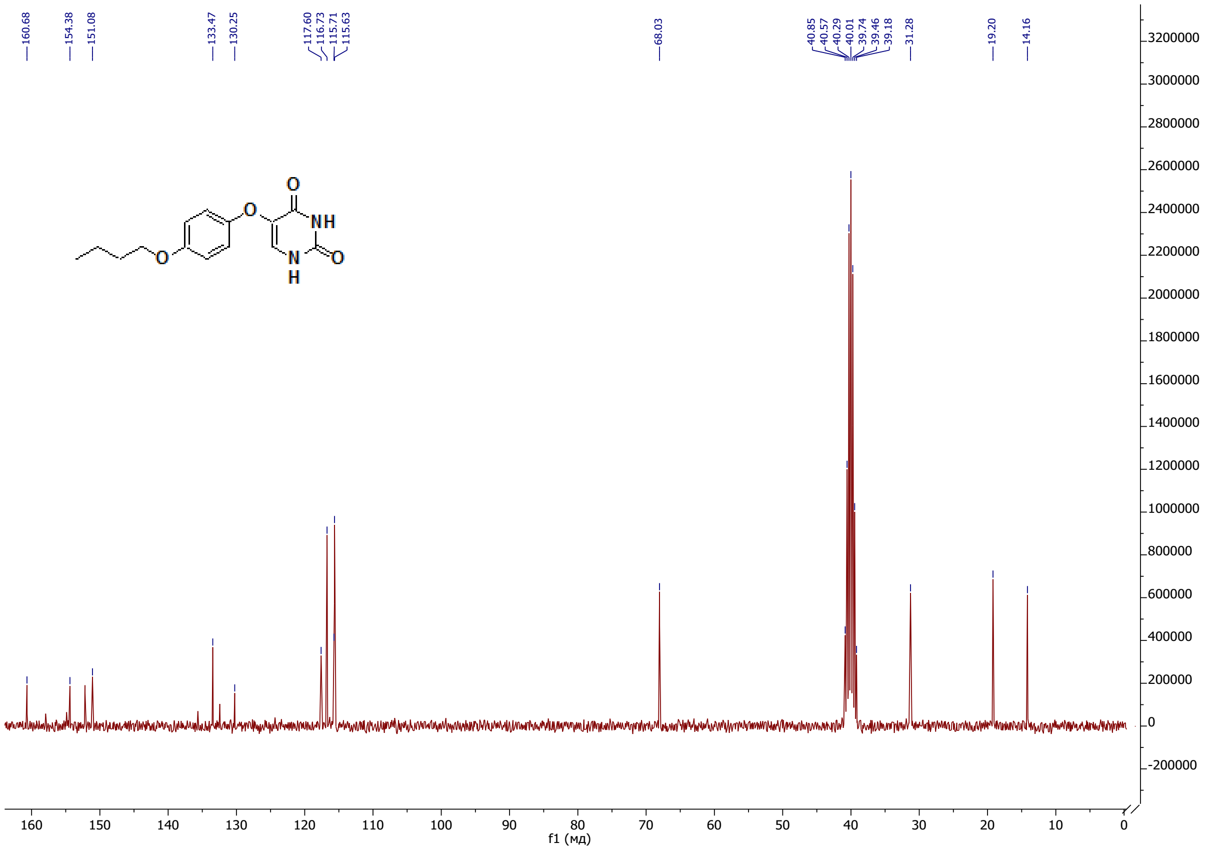

Figure S22 ^{13}C NMR spectrum of compound **5b** in DMSO-*d6* at 100 MHz.

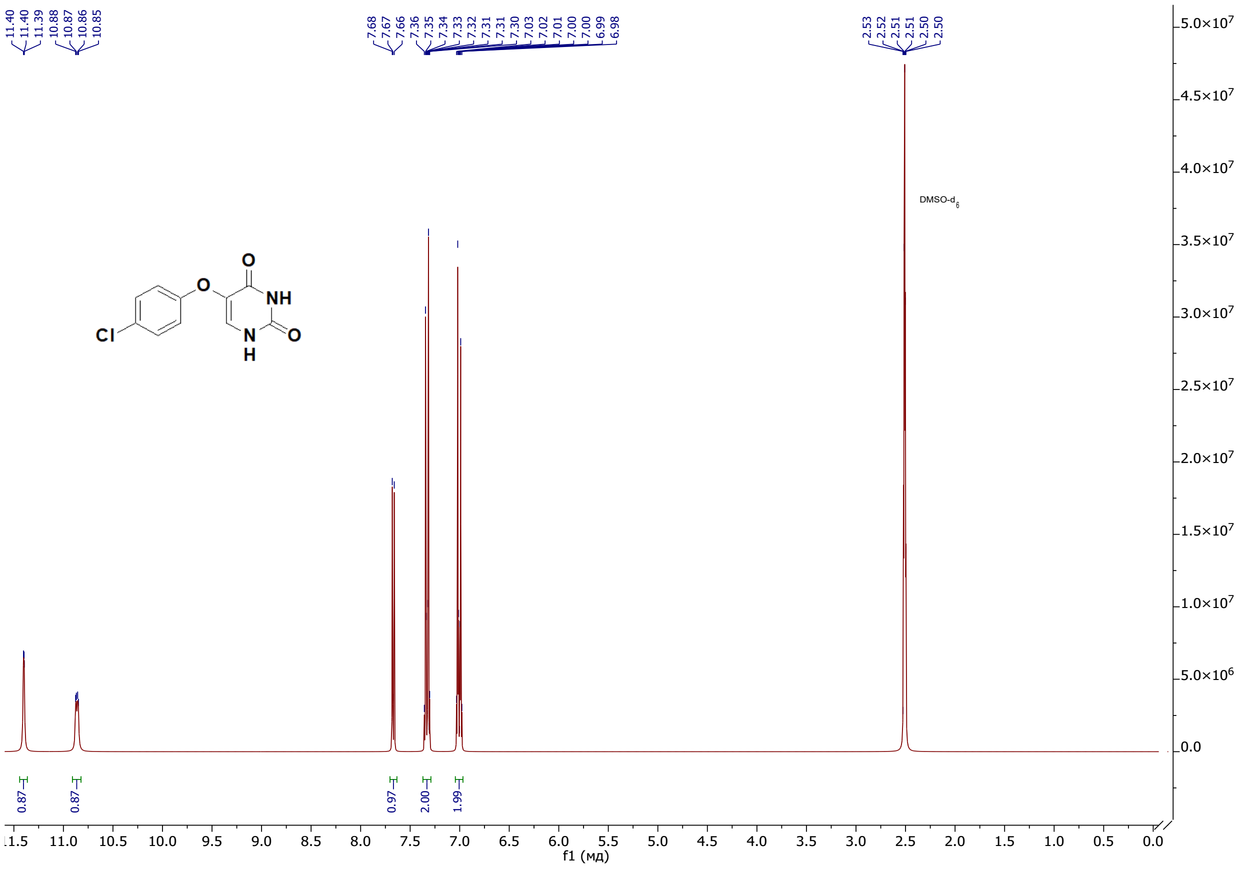

Figure S23 ^{1}H NMR spectrum of compound **5c** in DMSO-*d6* at 400 MHz.

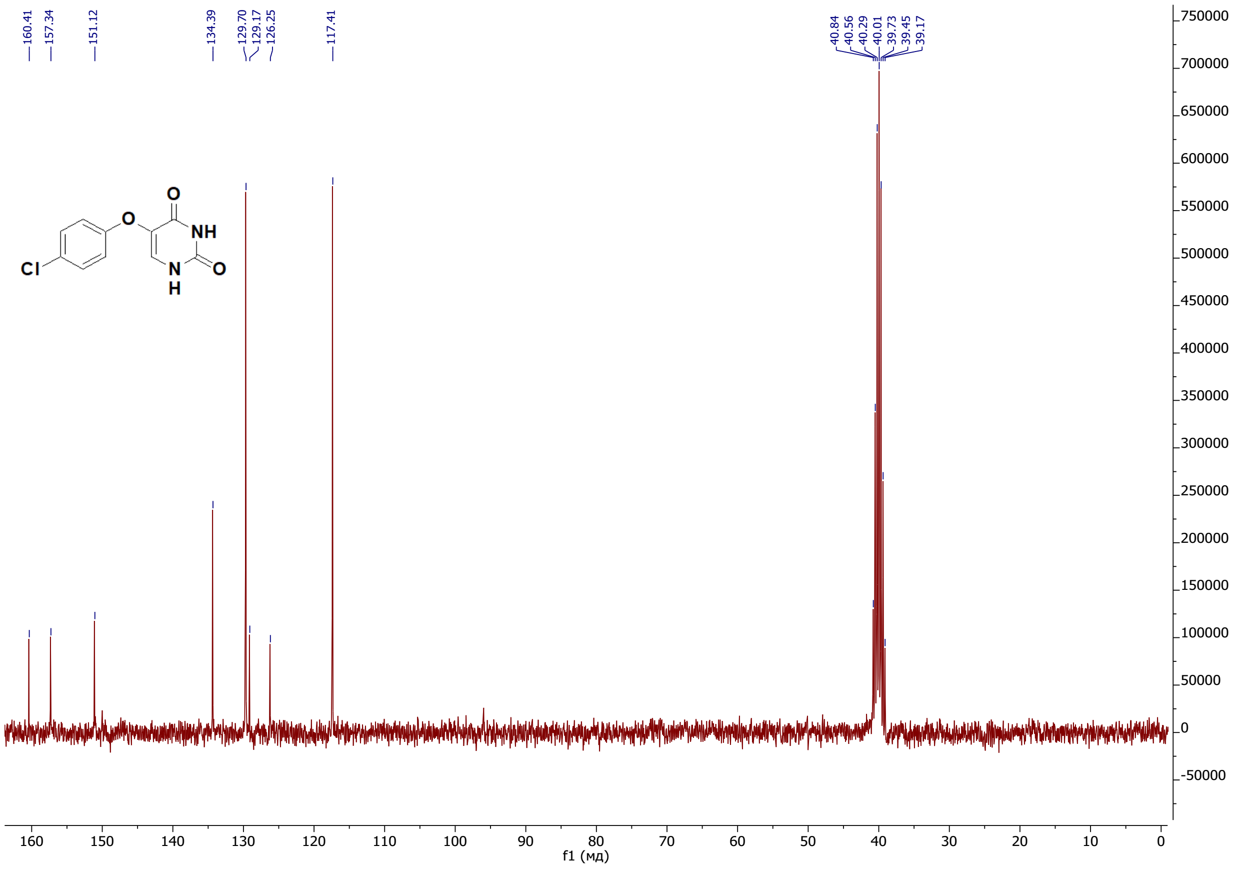

Figure S24 ^{13}C NMR spectrum of compound **5c** in DMSO-*d6* at 100 MHz.

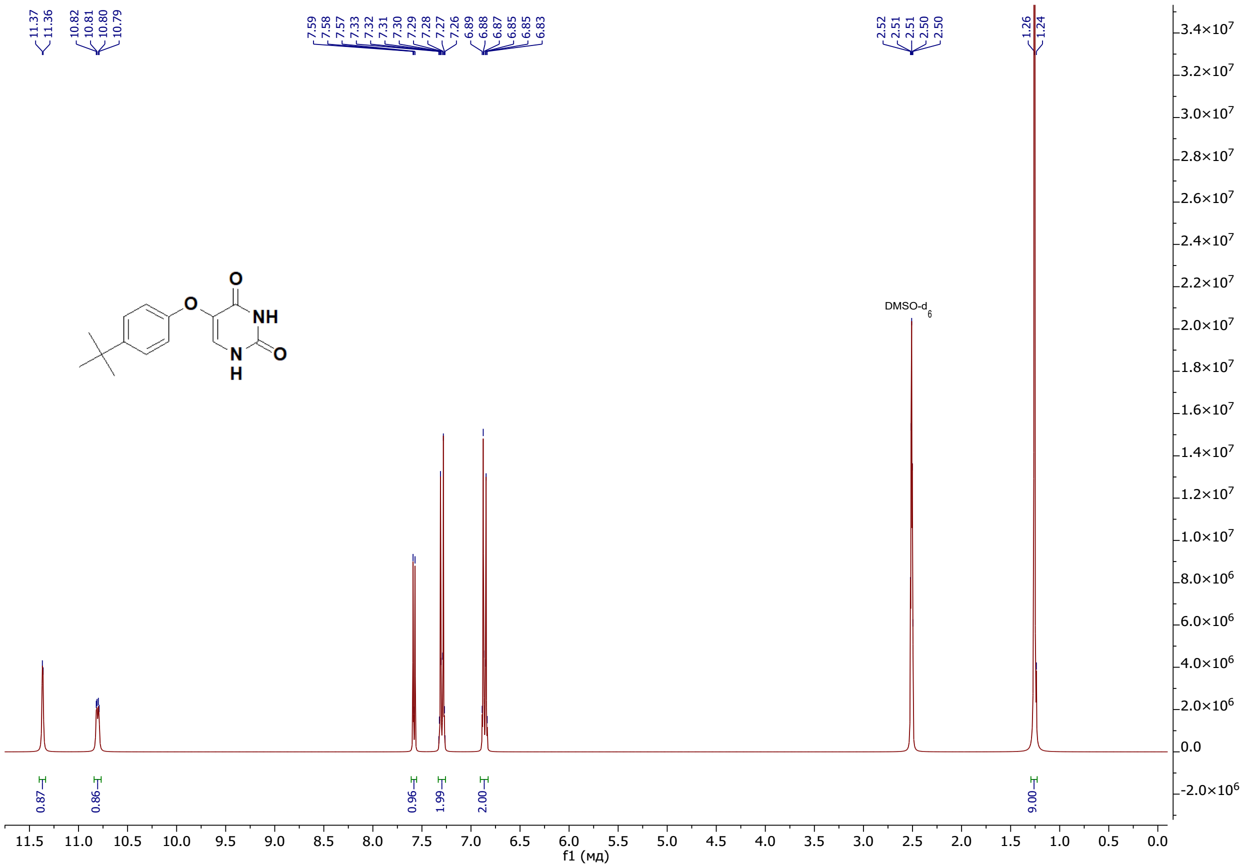

Figure S25 ^{1}H NMR spectrum of compound **5d** in DMSO-*d6* at 400 MHz.

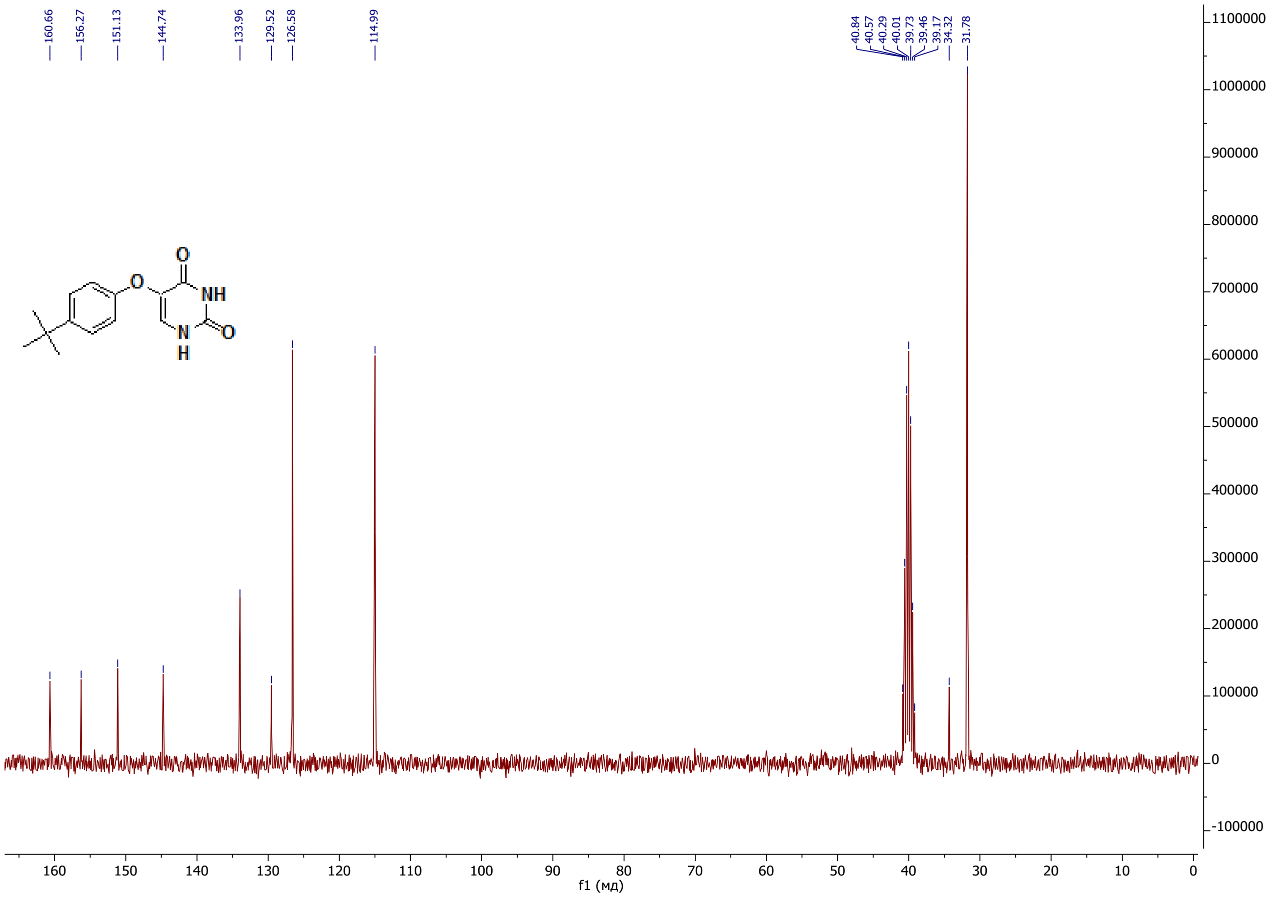

Figure S26 ^{13}C NMR spectrum of compound **5d** in DMSO-*d6* at 100 MHz.

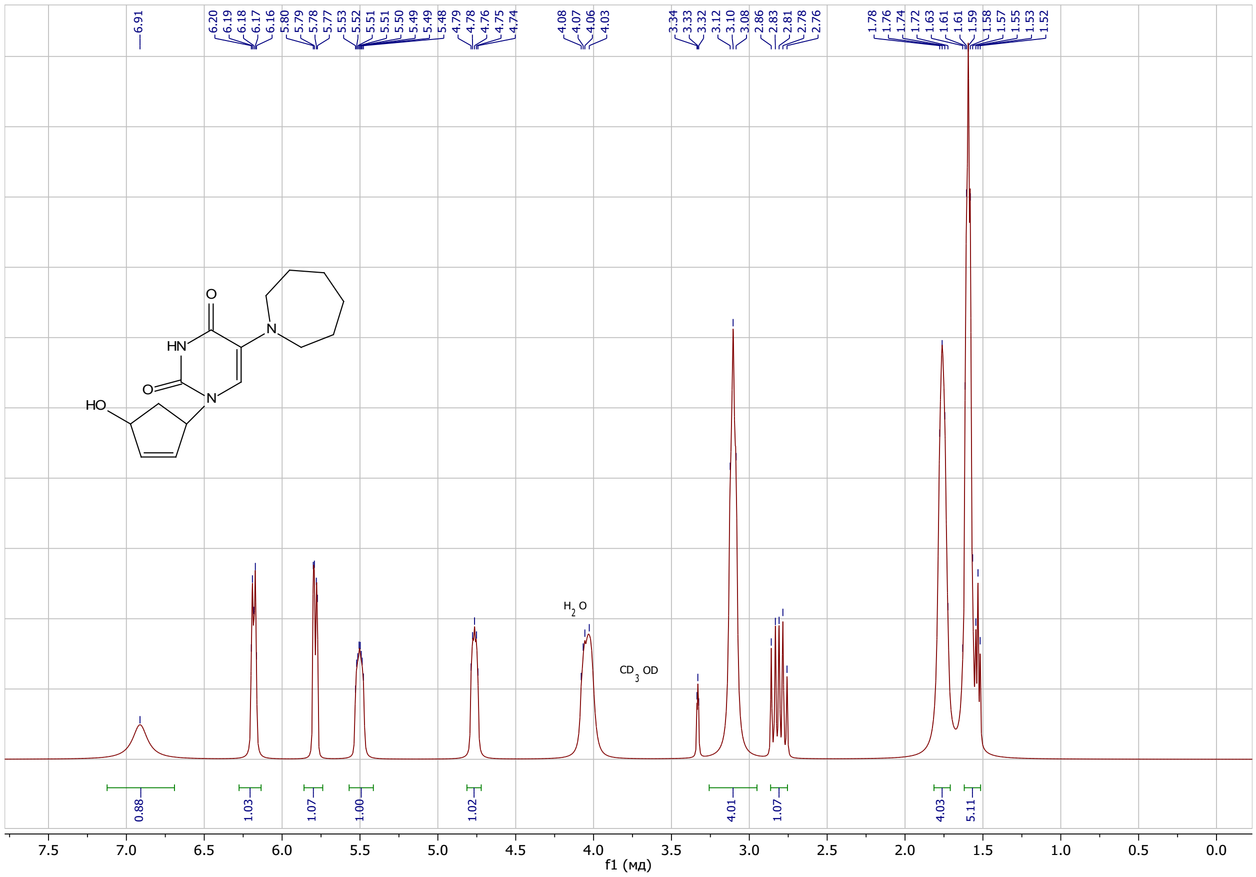

Figure S27 ^{1}H NMR spectrum of compound **3a** in $CDCl_3$ at 400 MHz.

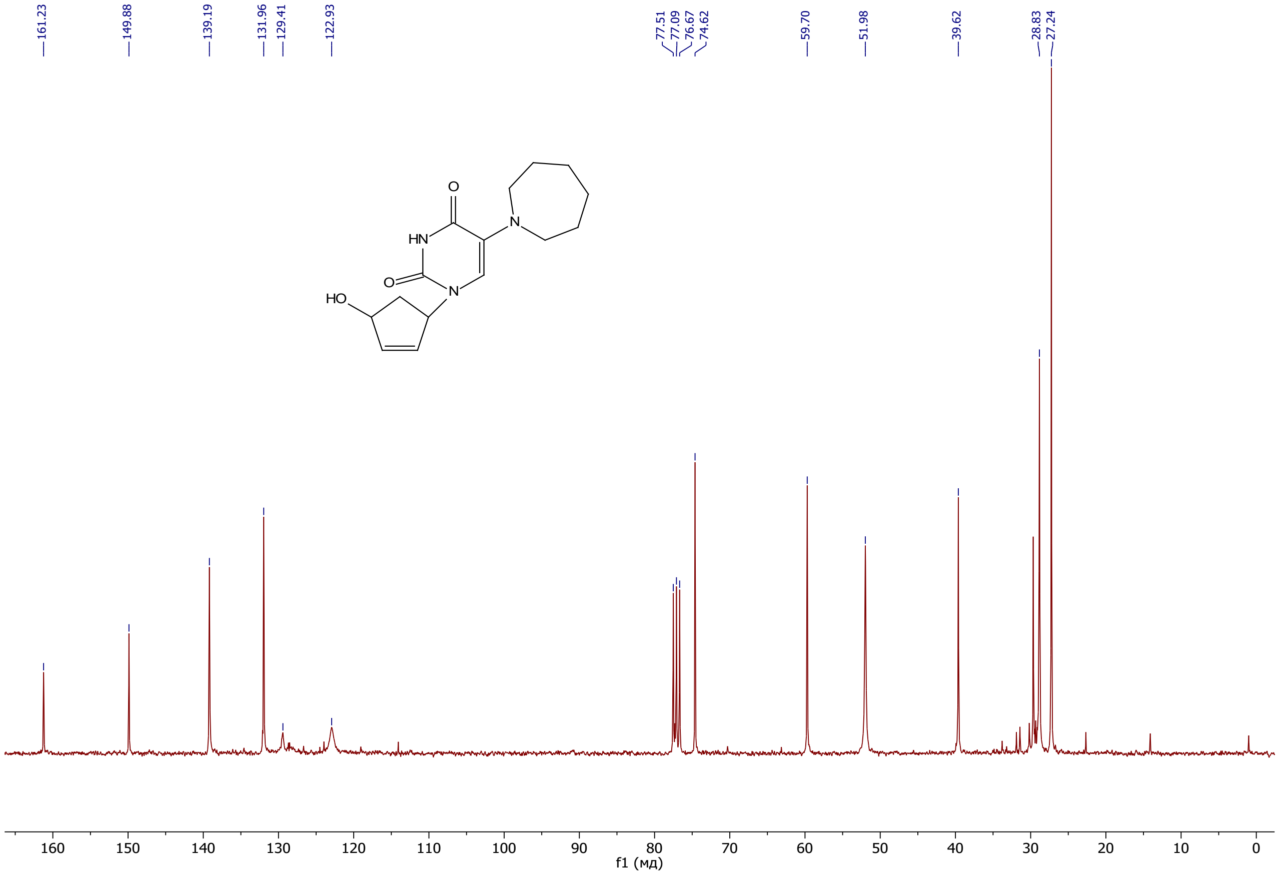

Figure S28 ^{13}C NMR spectrum of compound **3a** in $CDCl_3$ at 100 MHz.

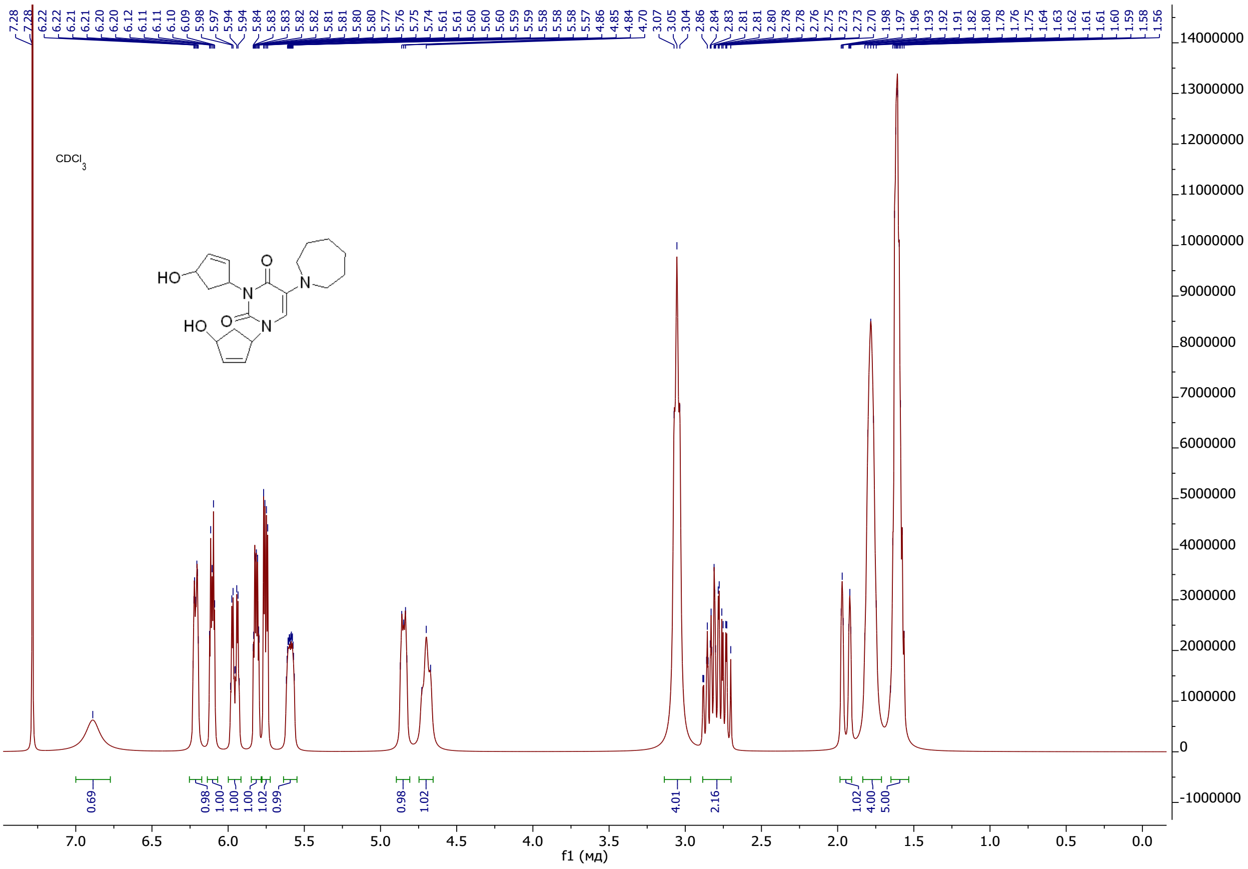

Figure S29 ^{1}H NMR spectrum of compound **4a** in $CDCl_3$ at 400 MHz.

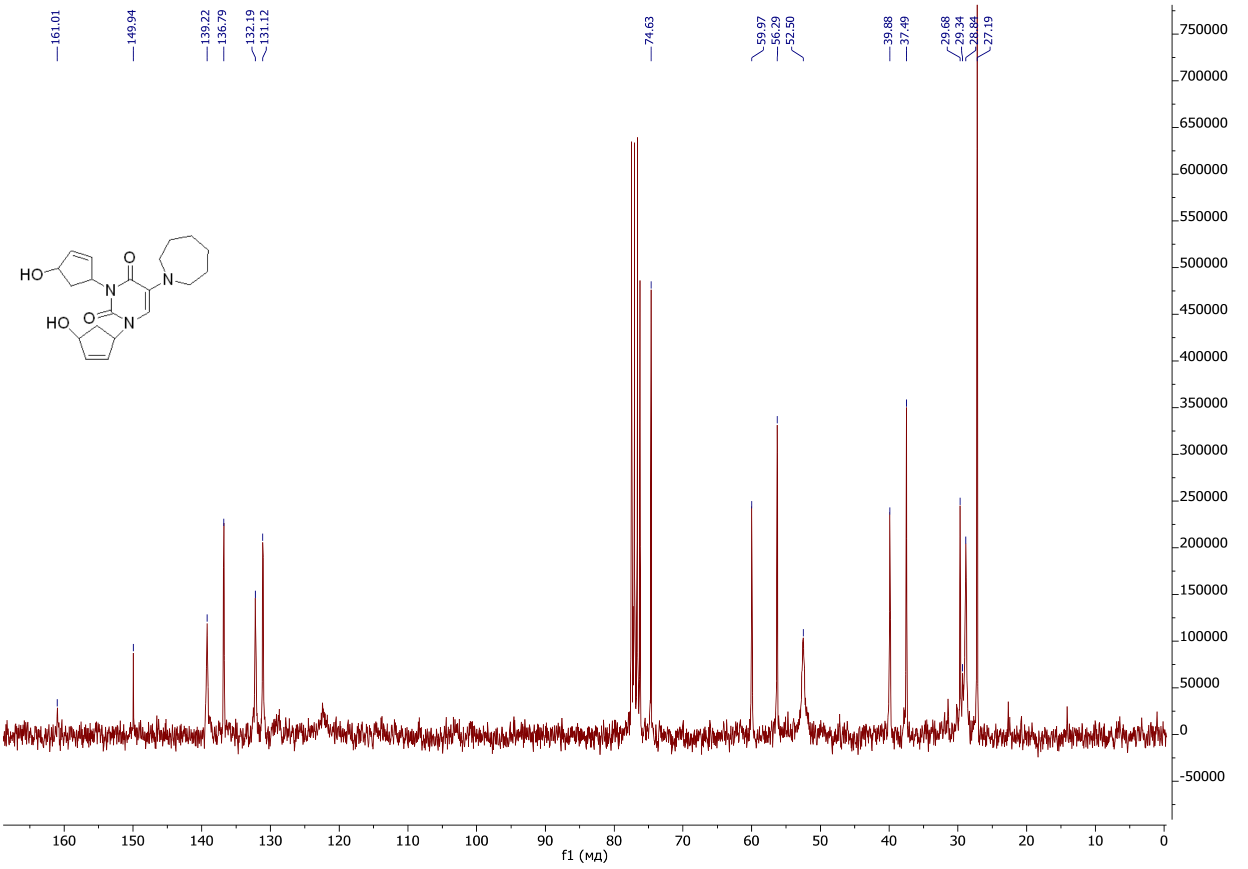

Figure S30 ^{13}C NMR spectrum of compound **4a** in $CDCl_3$ at 100 MHz.

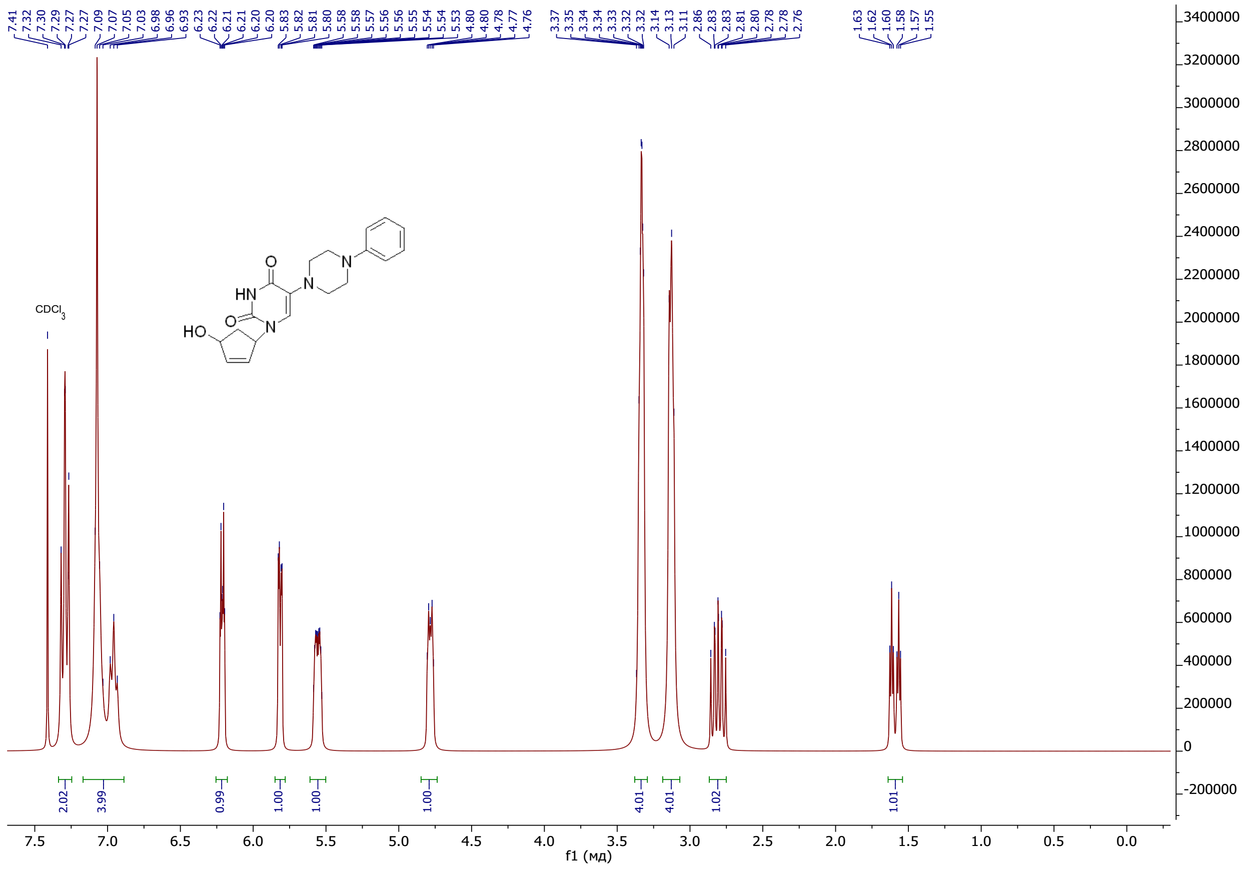

Figure S31 ^{1}H NMR spectrum of compound **3b** in $CDCl_3$ at 400 MHz.

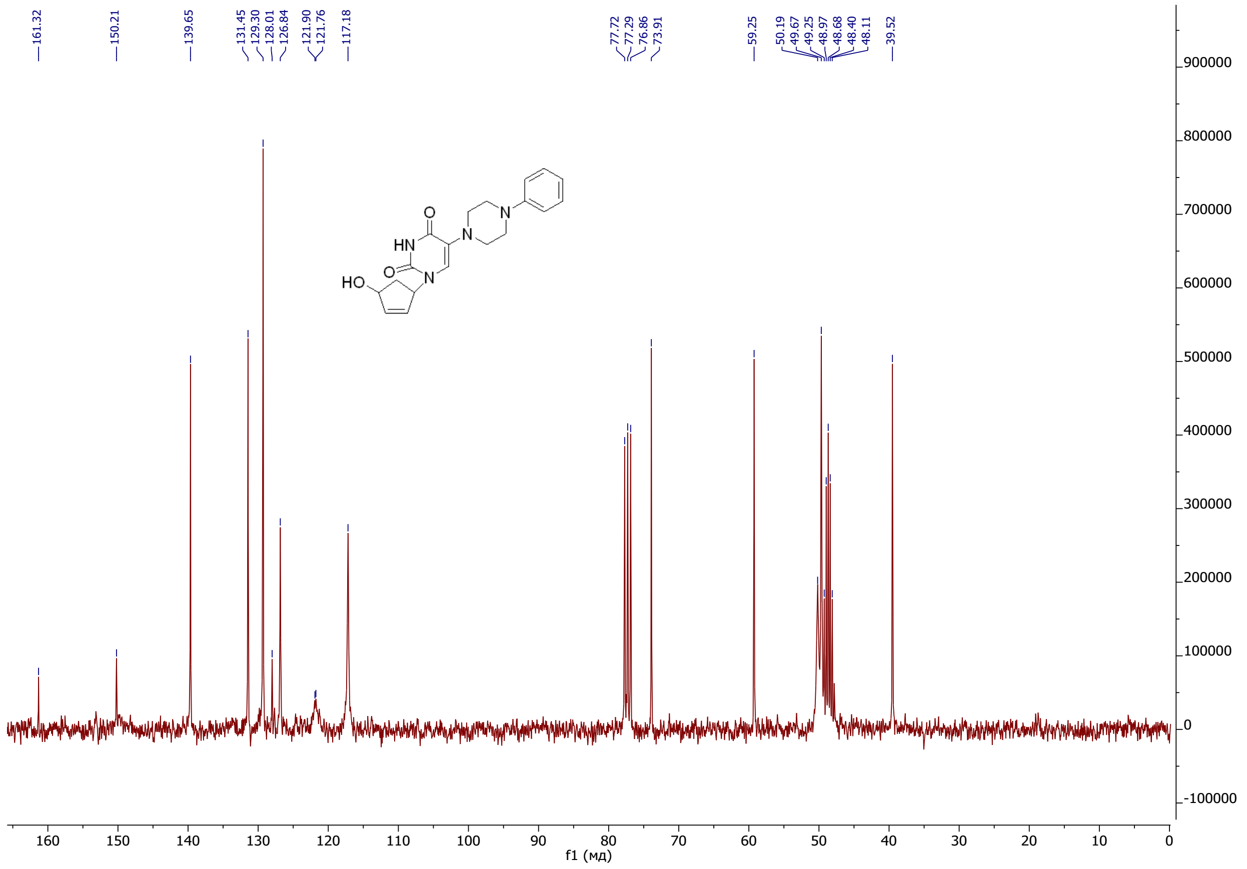

Figure S32 ^{13}C NMR spectrum of compound **3b** in $CDCl_3$:CD_3OD at 100 MHz.

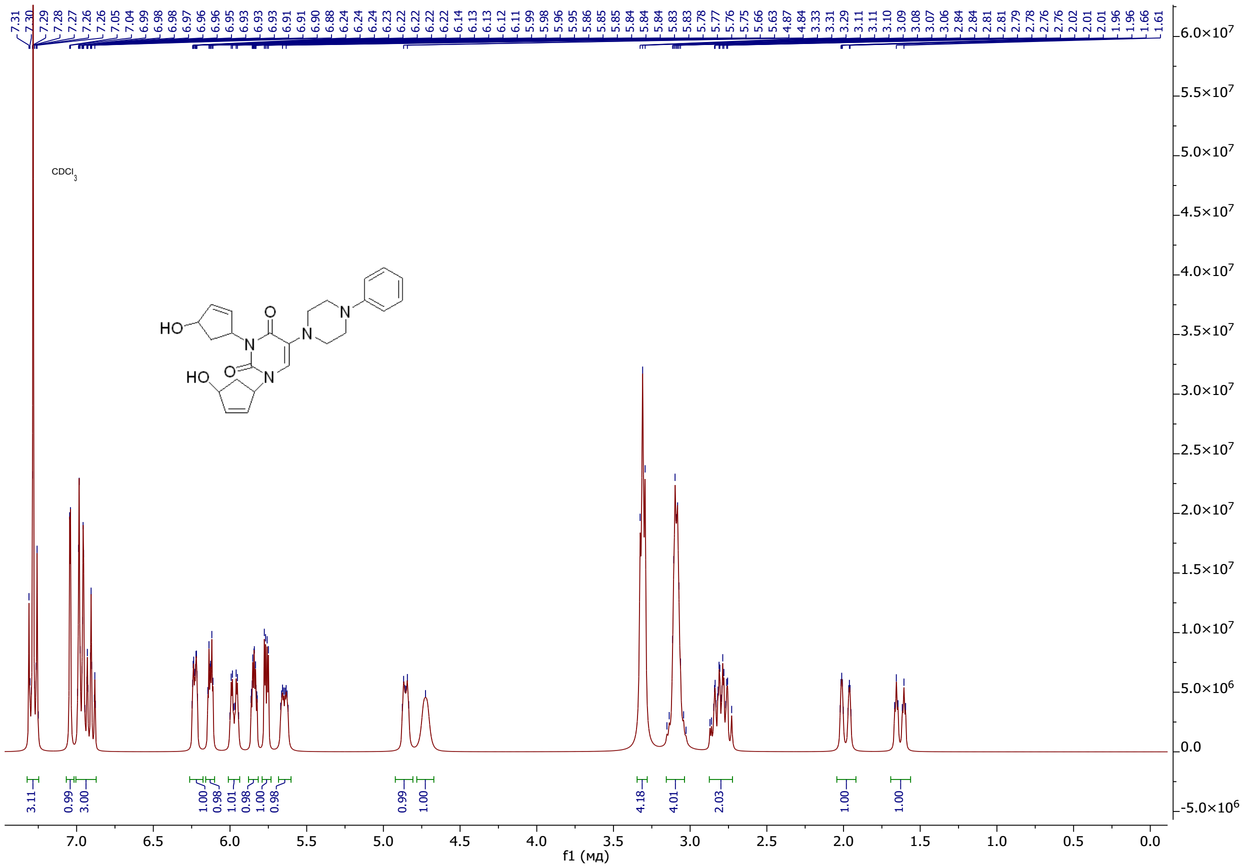

Figure S33 ^{1}H NMR spectrum of compound **4b** in $CDCl_3$ at 400 MHz.

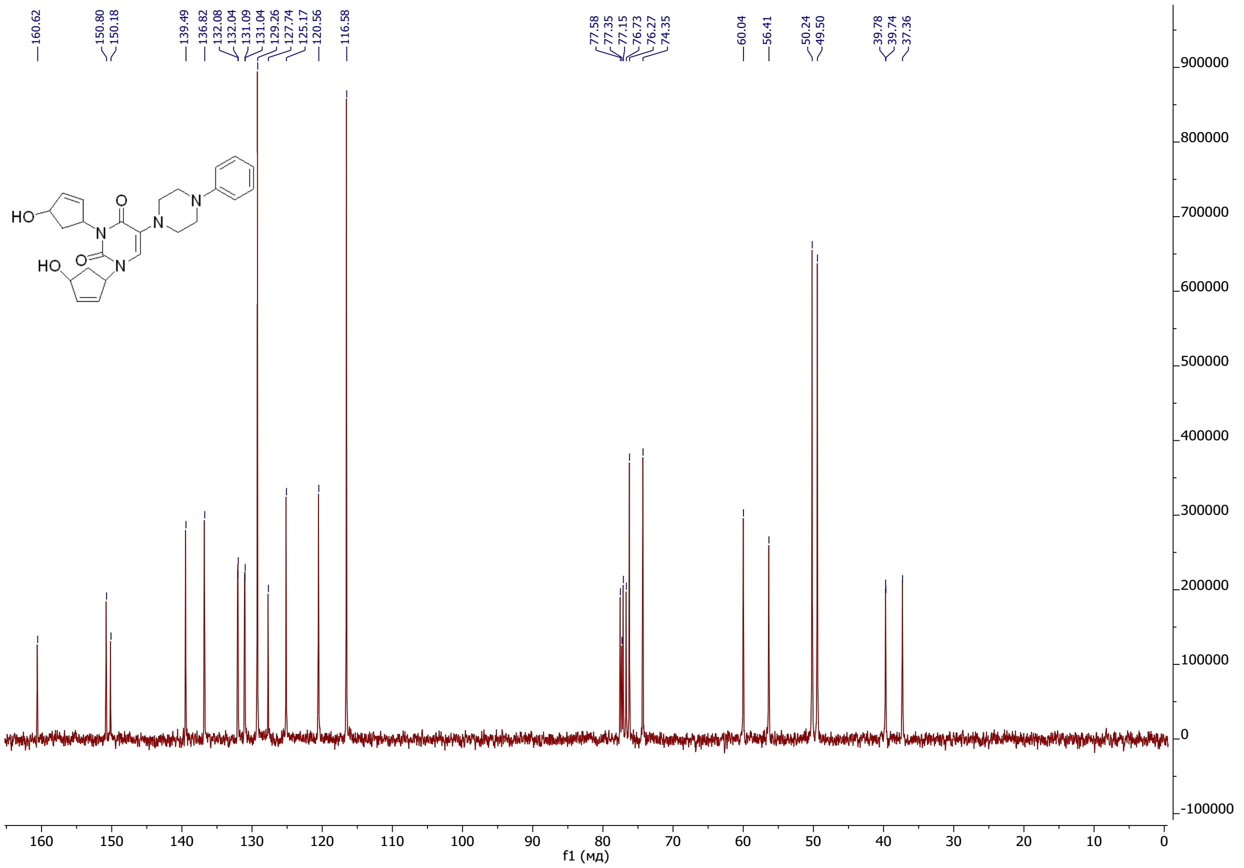

Figure S34 ^{13}C NMR spectrum of compound **4b** in $CDCl_3$ at 100 MHz.

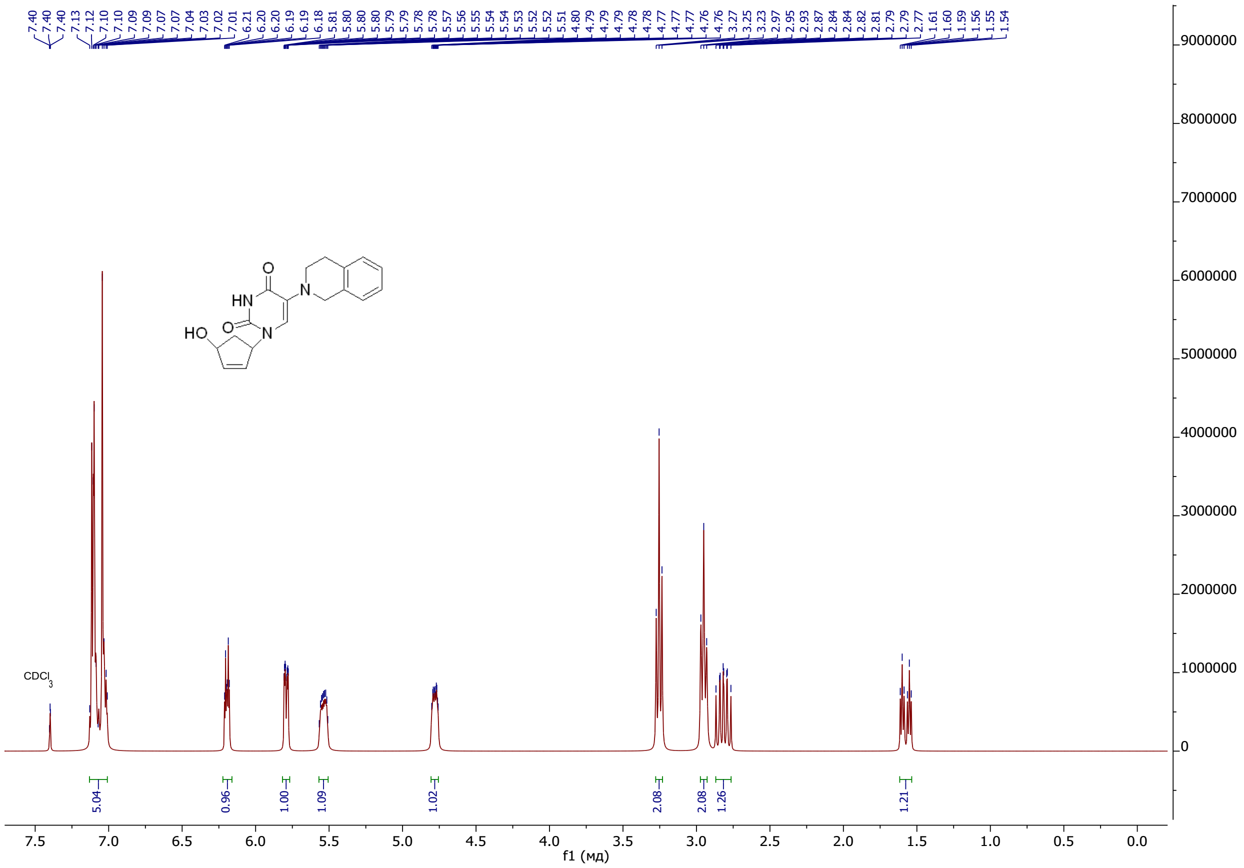

Figure S35 ^{1}H NMR spectrum of compound **3c** in $CDCl_3$:CD_3OD at 400 MHz.

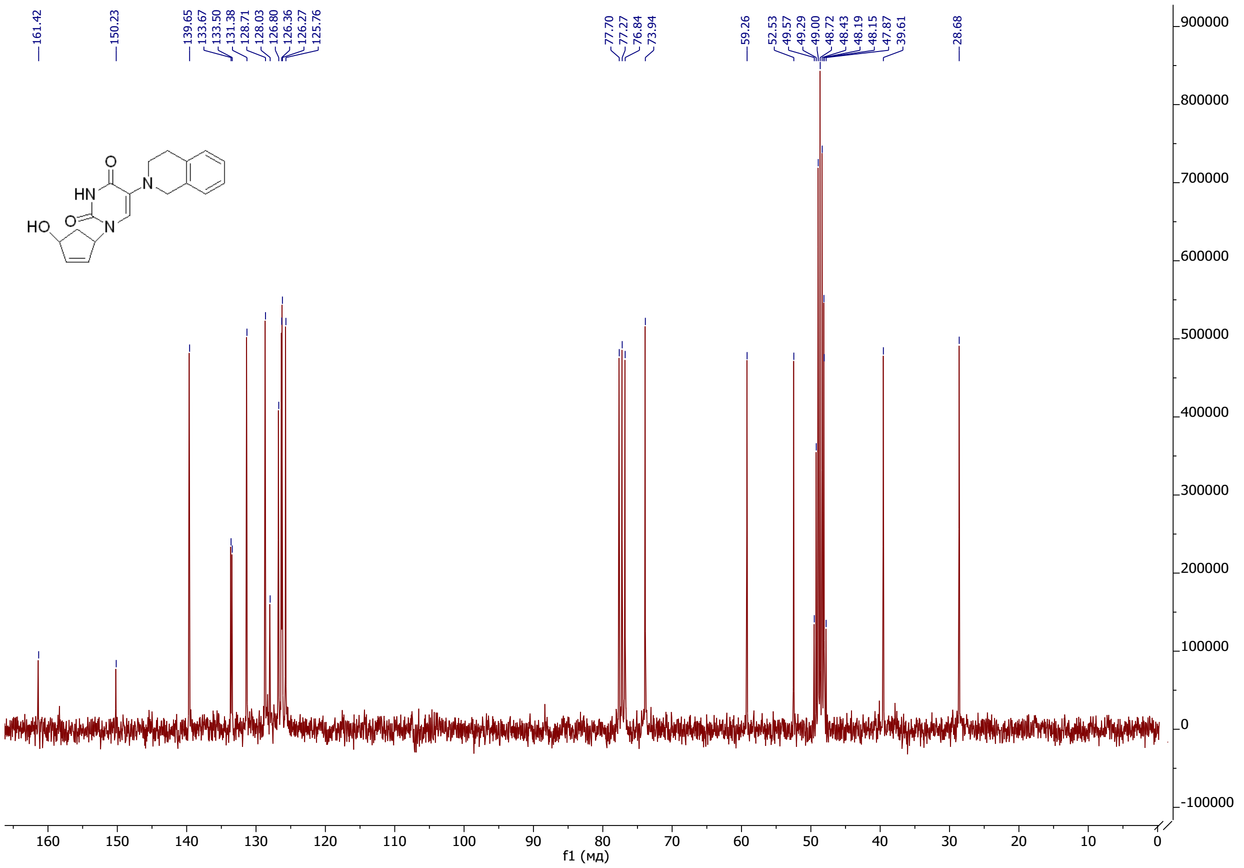

Figure S36 ^{13}C NMR spectrum of compound **3c** in $CDCl_3$:CD_3OD at 100 MHz.

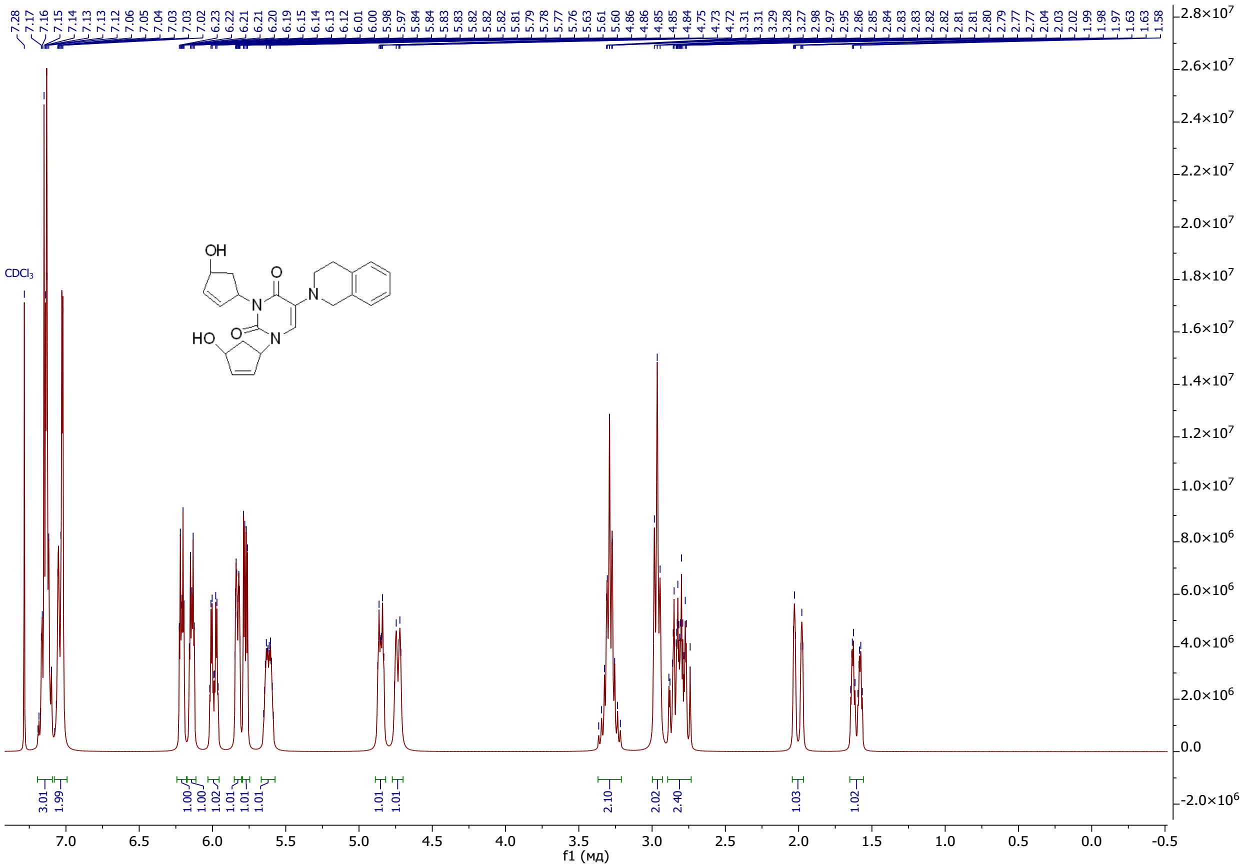

Figure S37 ^{1}H NMR spectrum of compound **4c** in $CDCl_3$ at 400 MHz.

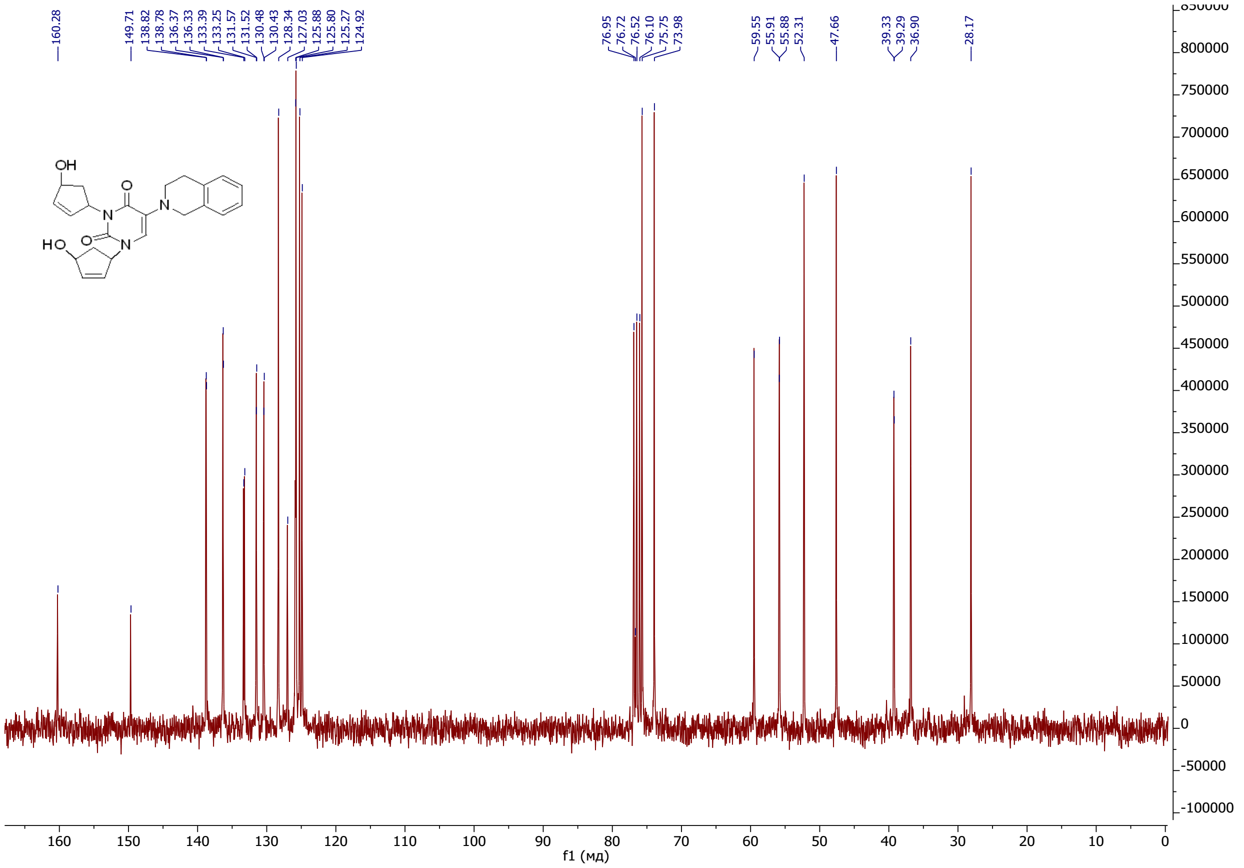

Figure S38 ^{13}C NMR spectrum of compound **4c** in $CDCl_3$ at 100 MHz.

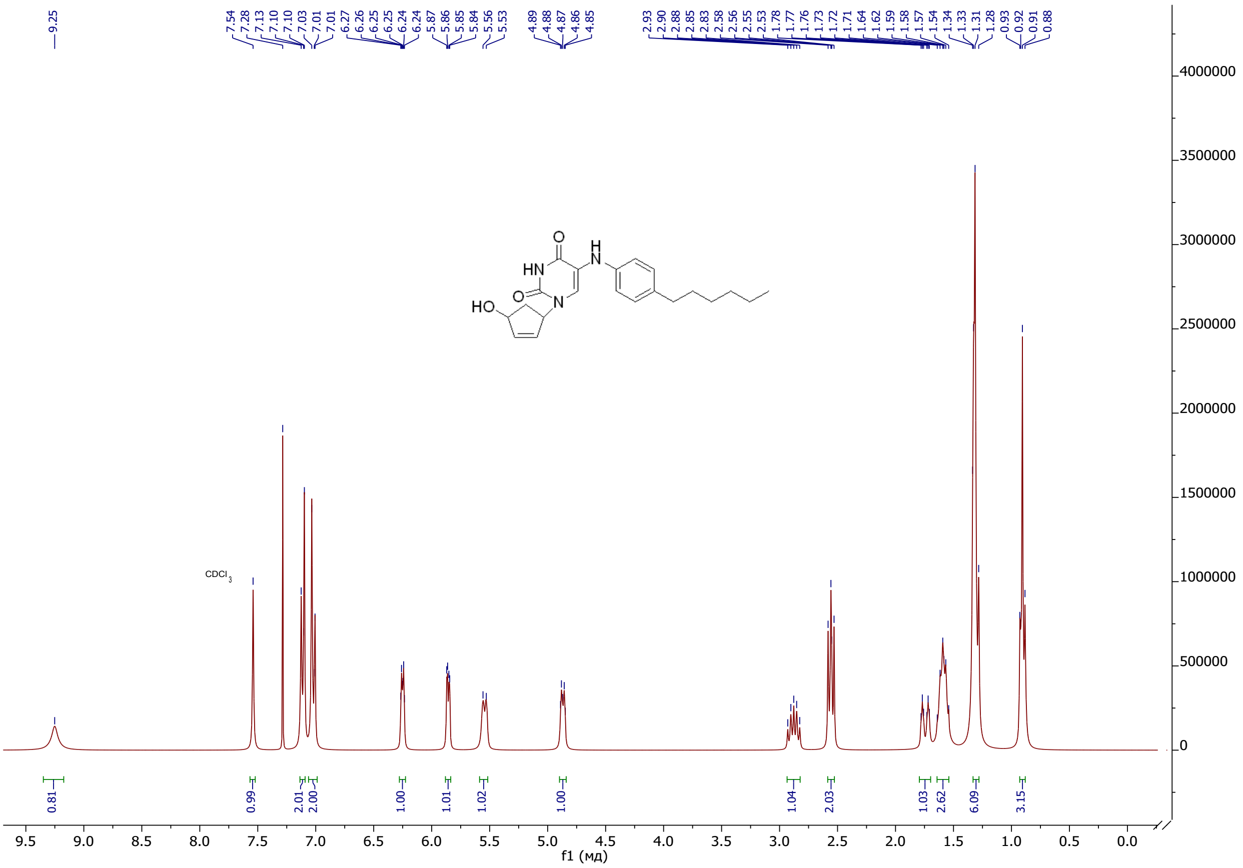

Figure S39 ^{1}H NMR spectrum of compound **3d** in $CDCl_3$ at 400 MHz.

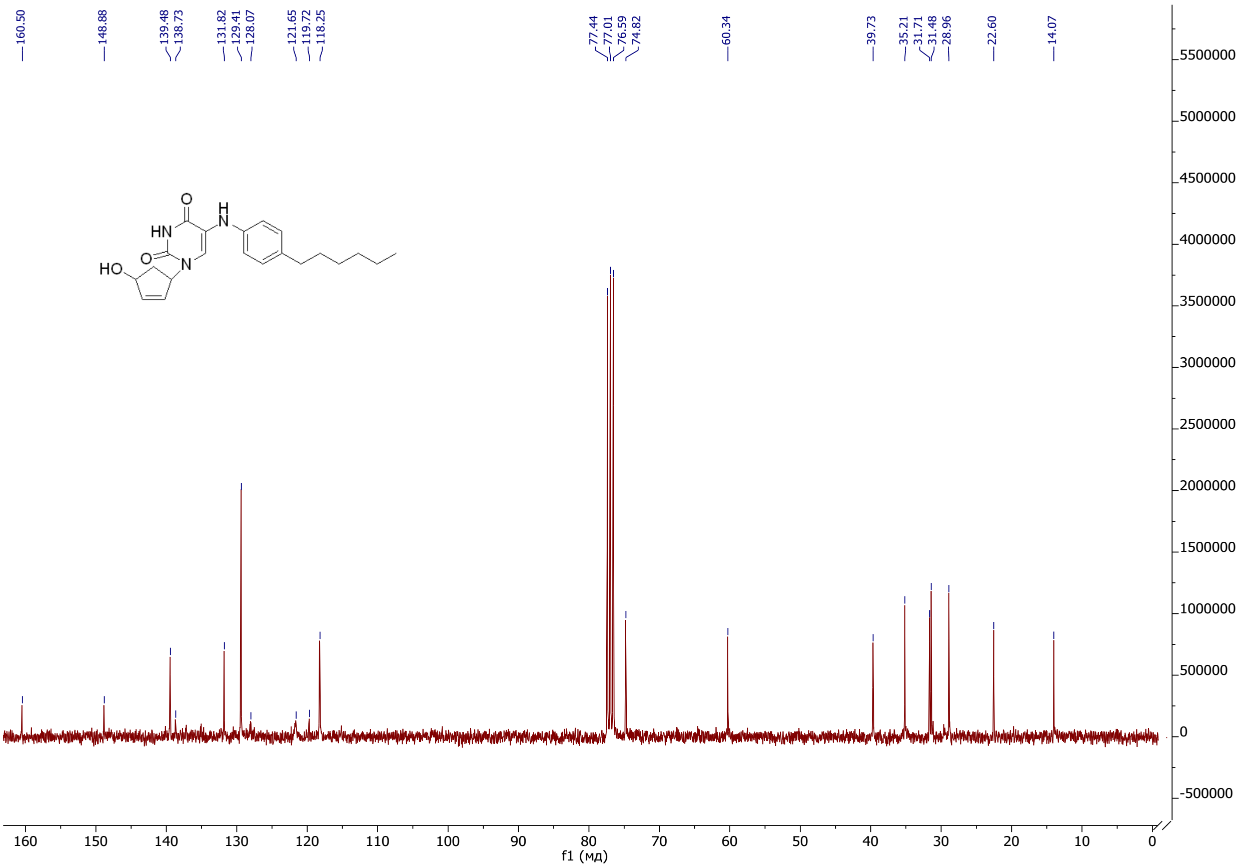

Figure S40 ^{13}C NMR spectrum of compound **3d** in $CDCl_3$ at 100 MHz.

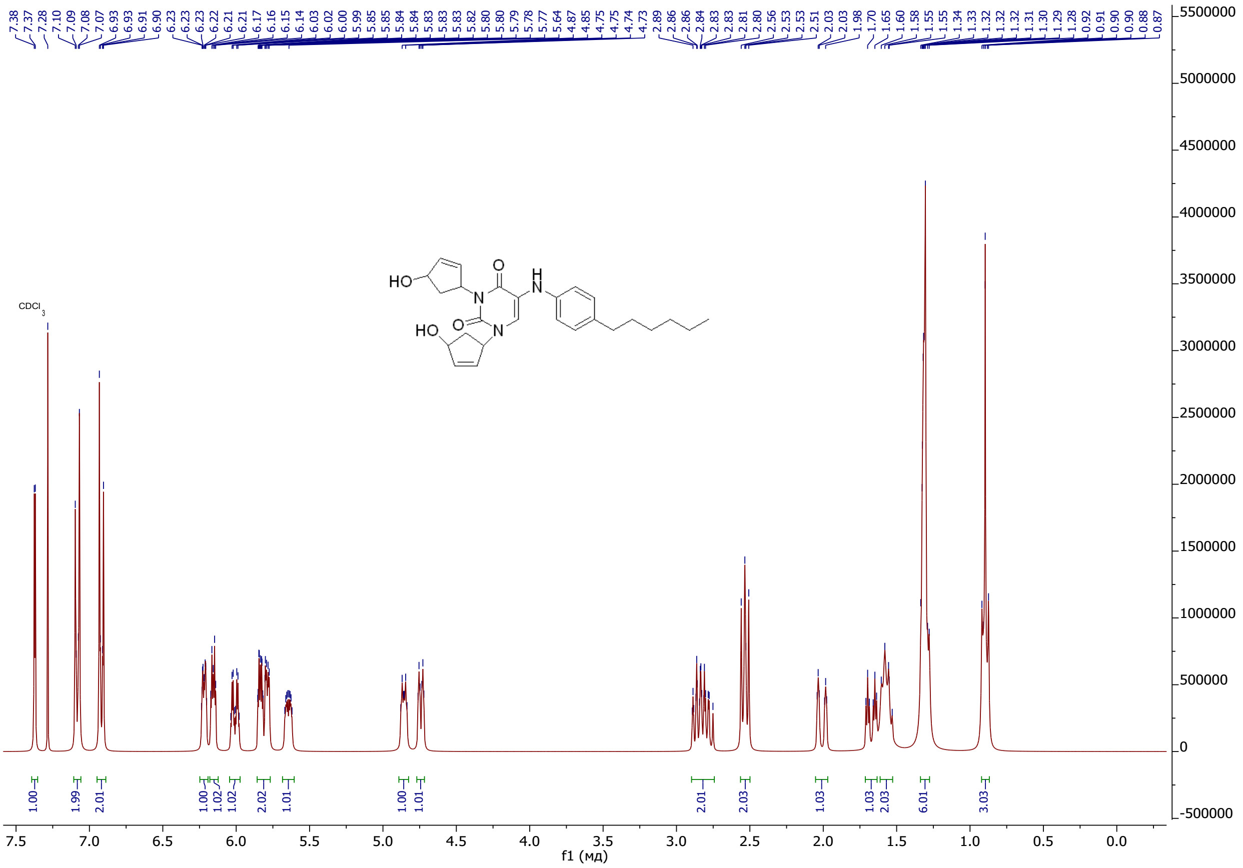

Figure S41 ^{1}H NMR spectrum of compound **4d** in $CDCl_3$ at 400 MHz.

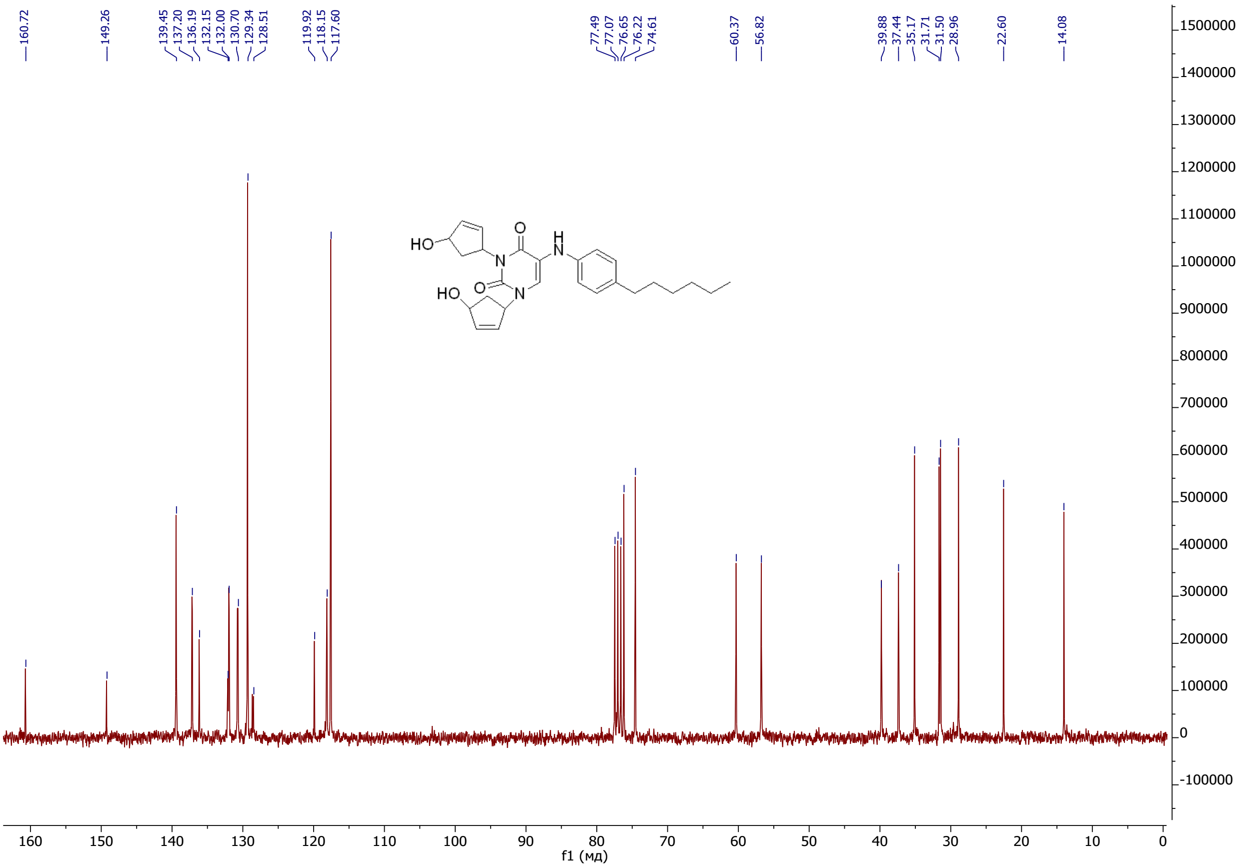

Figure S42 ^{13}C NMR spectrum of compound **4d** in $CDCl_3$ at 100 MHz.

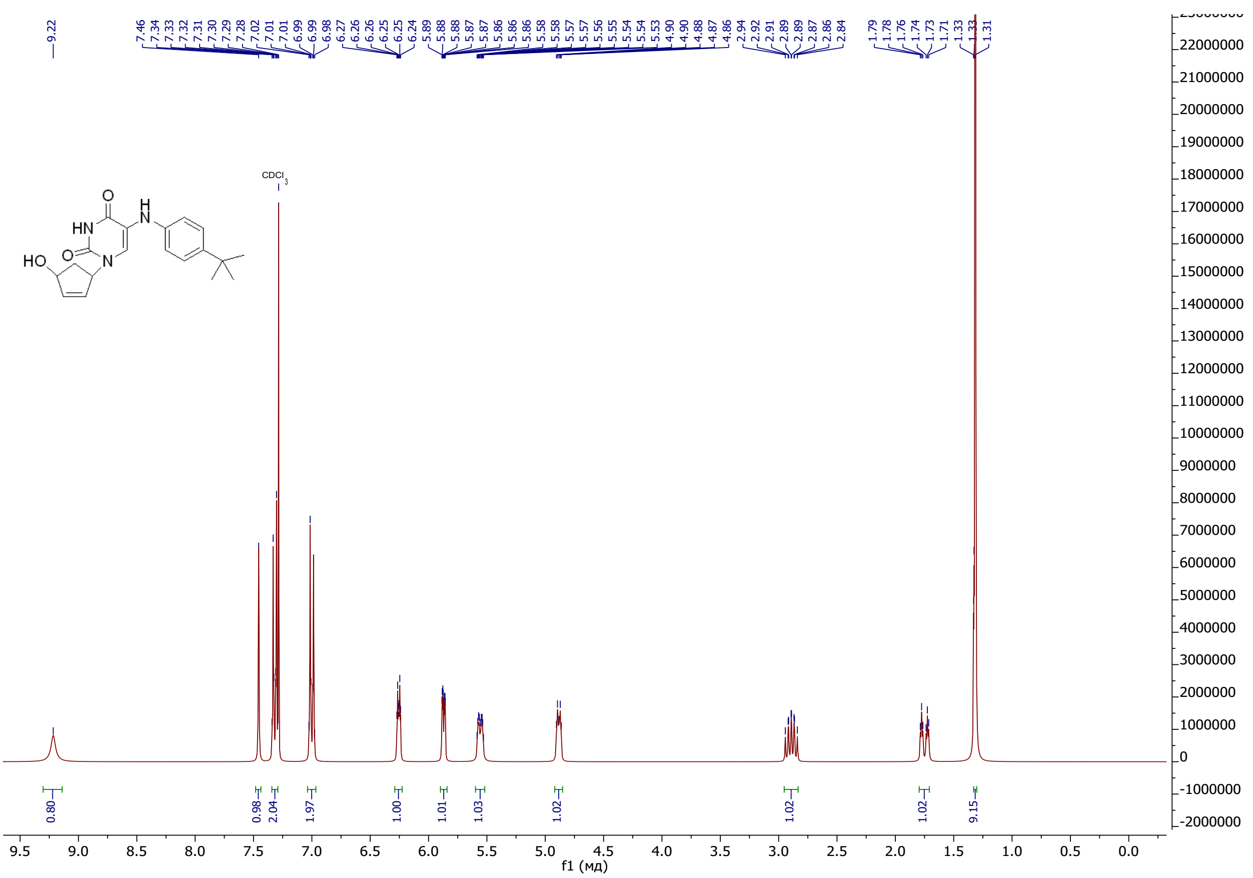

Figure S43 ^{1}H NMR spectrum of compound **3e** in $CDCl_3$ at 400 MHz.

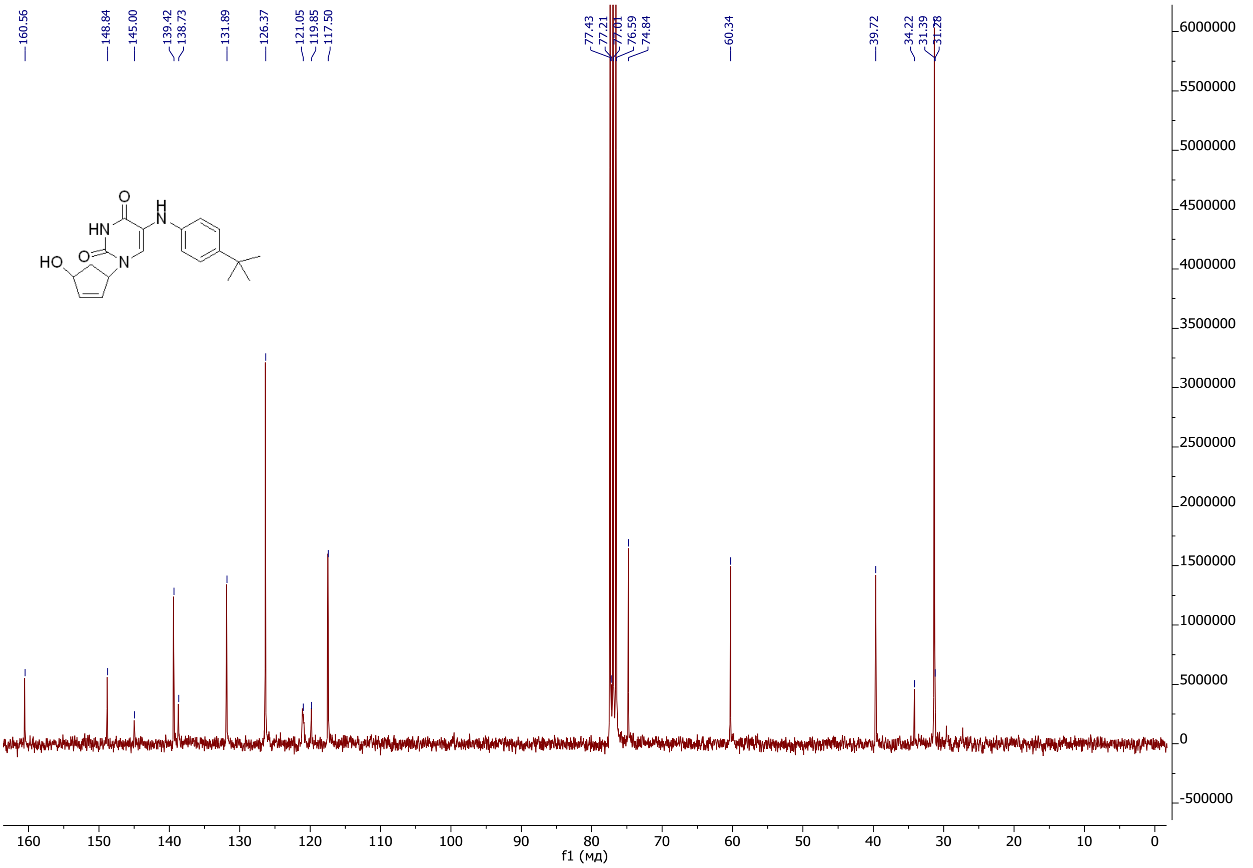

Figure S44 ^{13}C NMR spectrum of compound **3e** in $CDCl_3$ at 100 MHz.

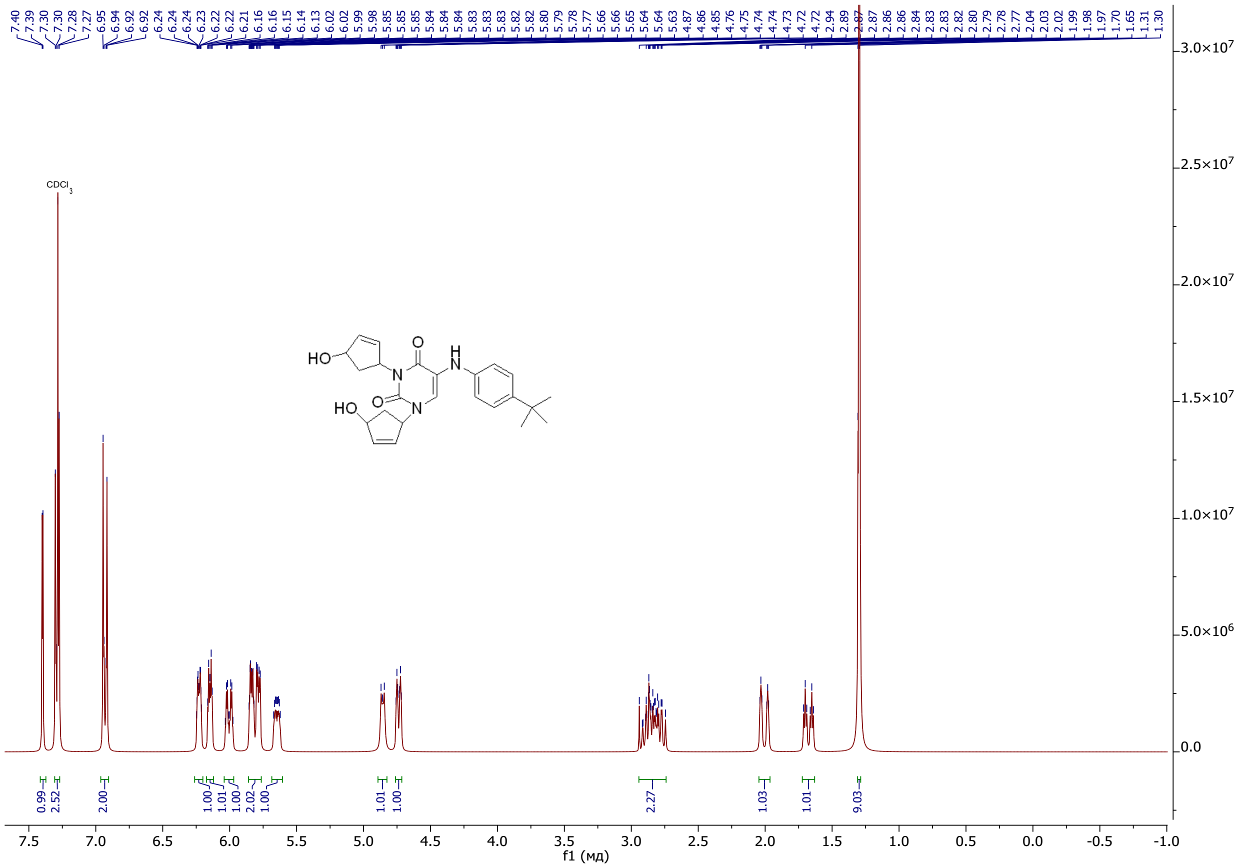

Figure S45 ^{1}H NMR spectrum of compound **4e** in $CDCl_3$ at 400 MHz.

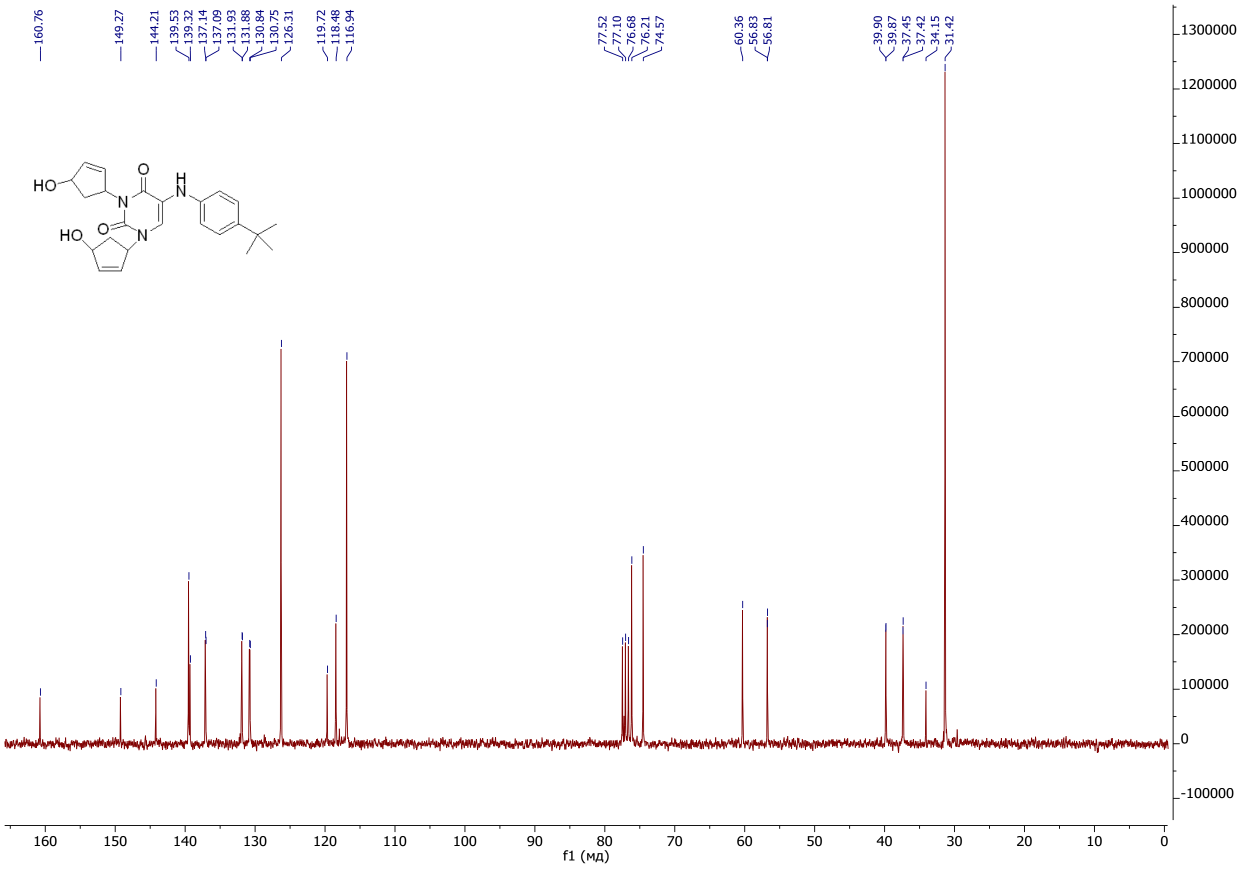

Figure S46 ^{13}C NMR spectrum of compound **4e** in $CDCl_3$ at 100 MHz.

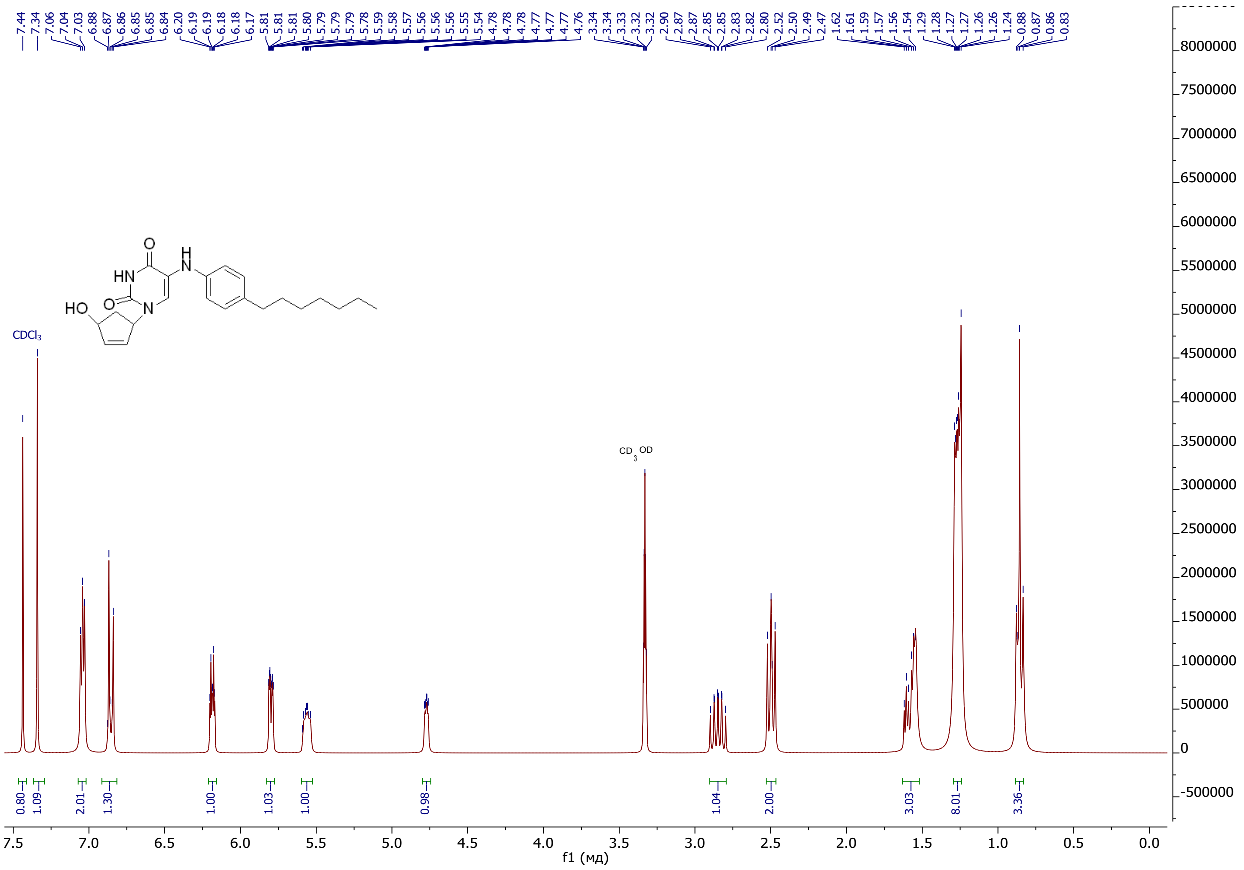

Figure S47 ^{1}H NMR spectrum of compound **3f** in $CDCl_3$:CD_3OD at 400 MHz.

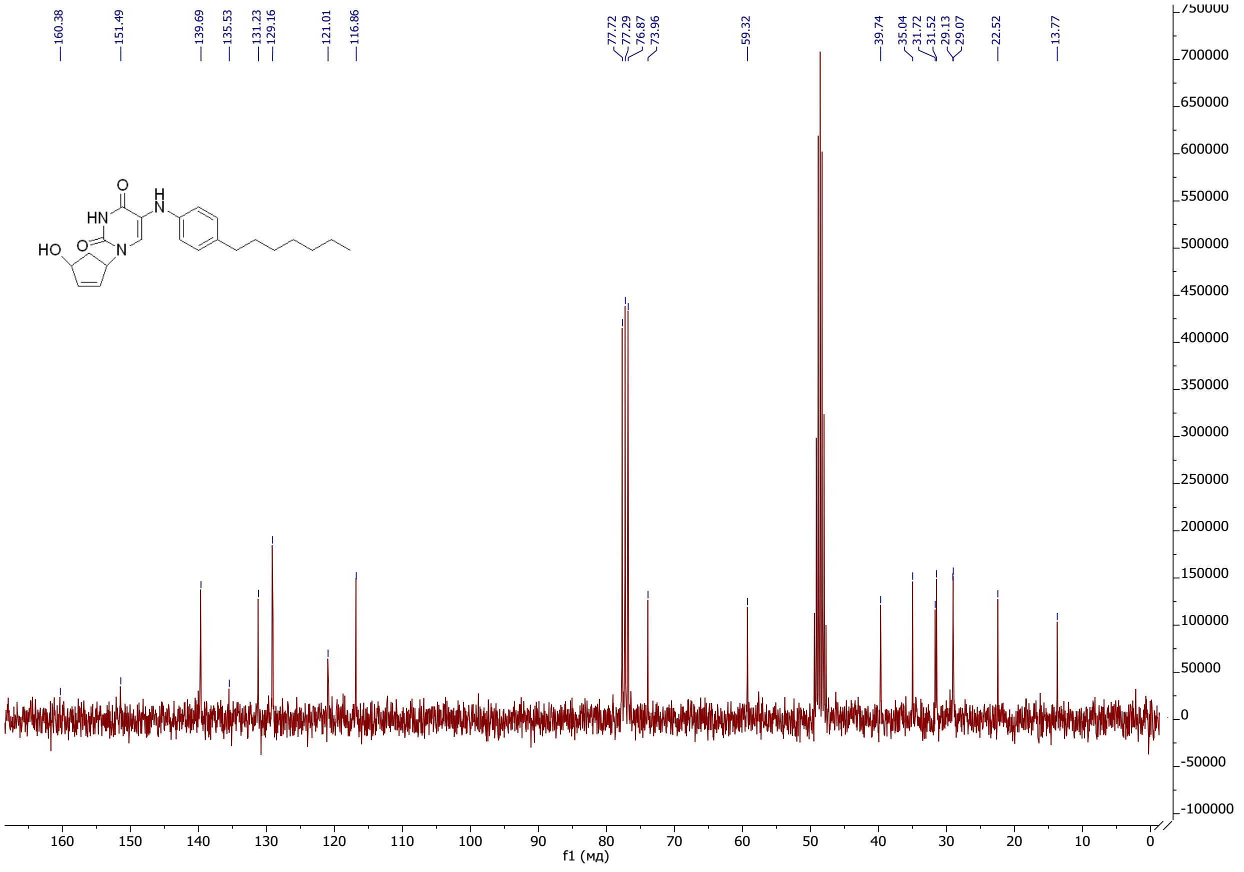

Figure S48 ^{13}C NMR spectrum of compound **3f** in $CDCl_3$:CD_3OD at 100 MHz.

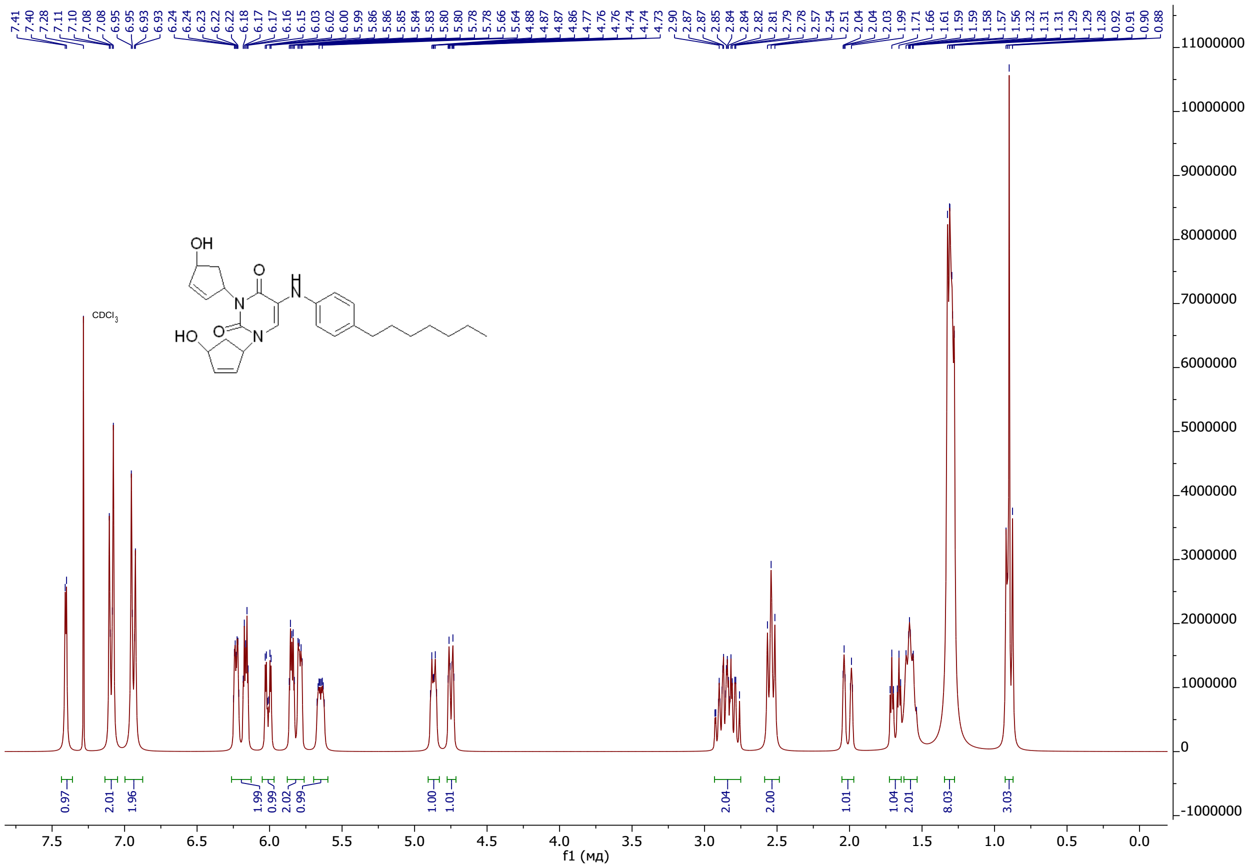

Figure S49 ^{1}H NMR spectrum of compound **4f** in $CDCl_3$ at 400 MHz.

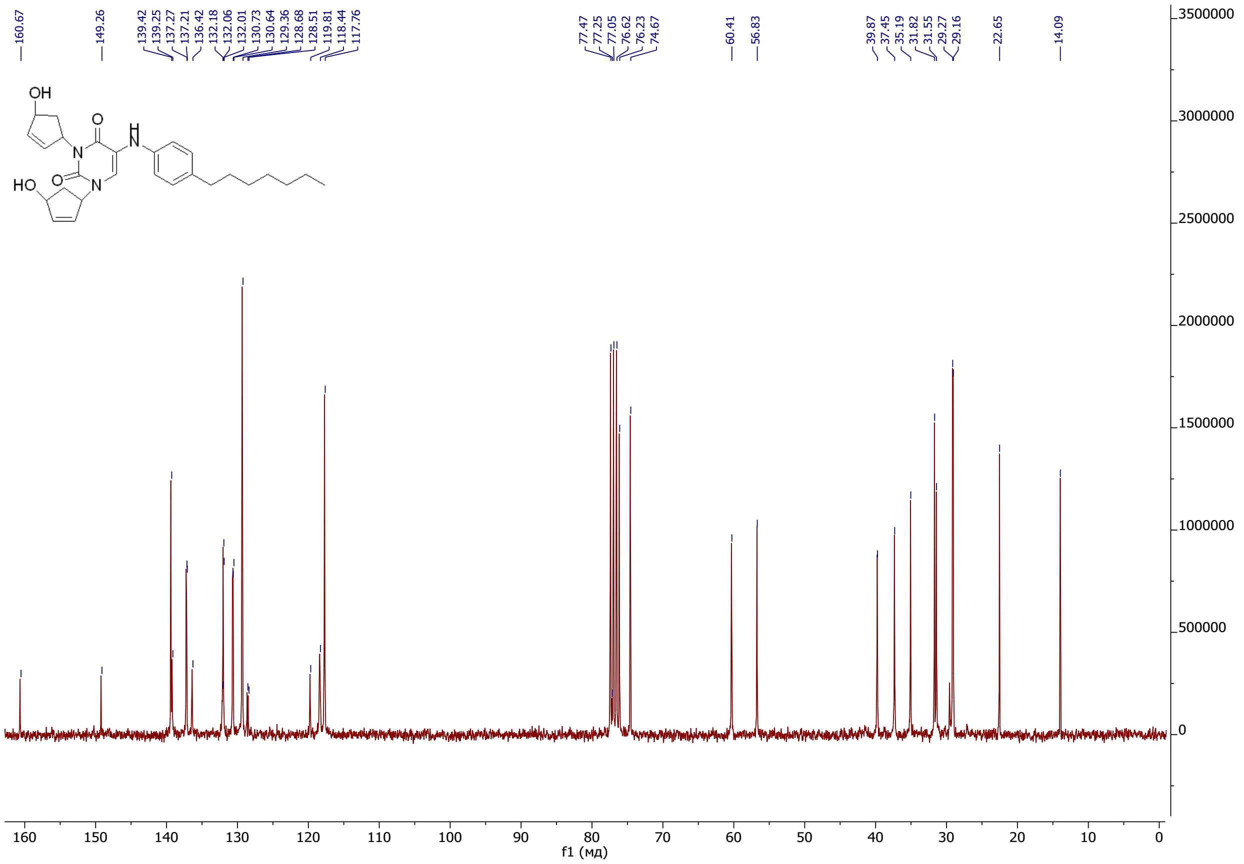

Figure S50 ^{13}C NMR spectrum of compound **4f** in $CDCl_3$ at 100 MHz.

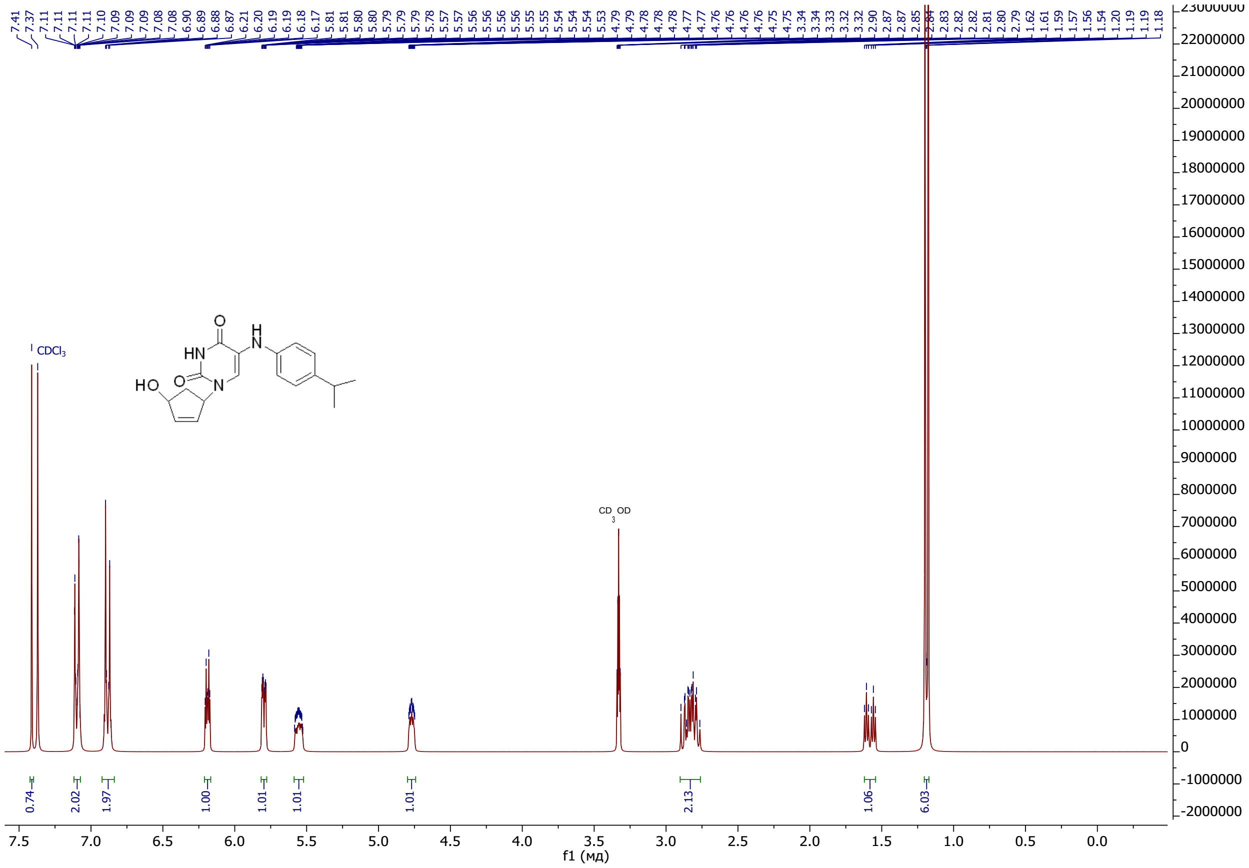

Figure S51 ^{1}H NMR spectrum of compound **3g** in $CDCl_3$:CD_3OD at 400 MHz.

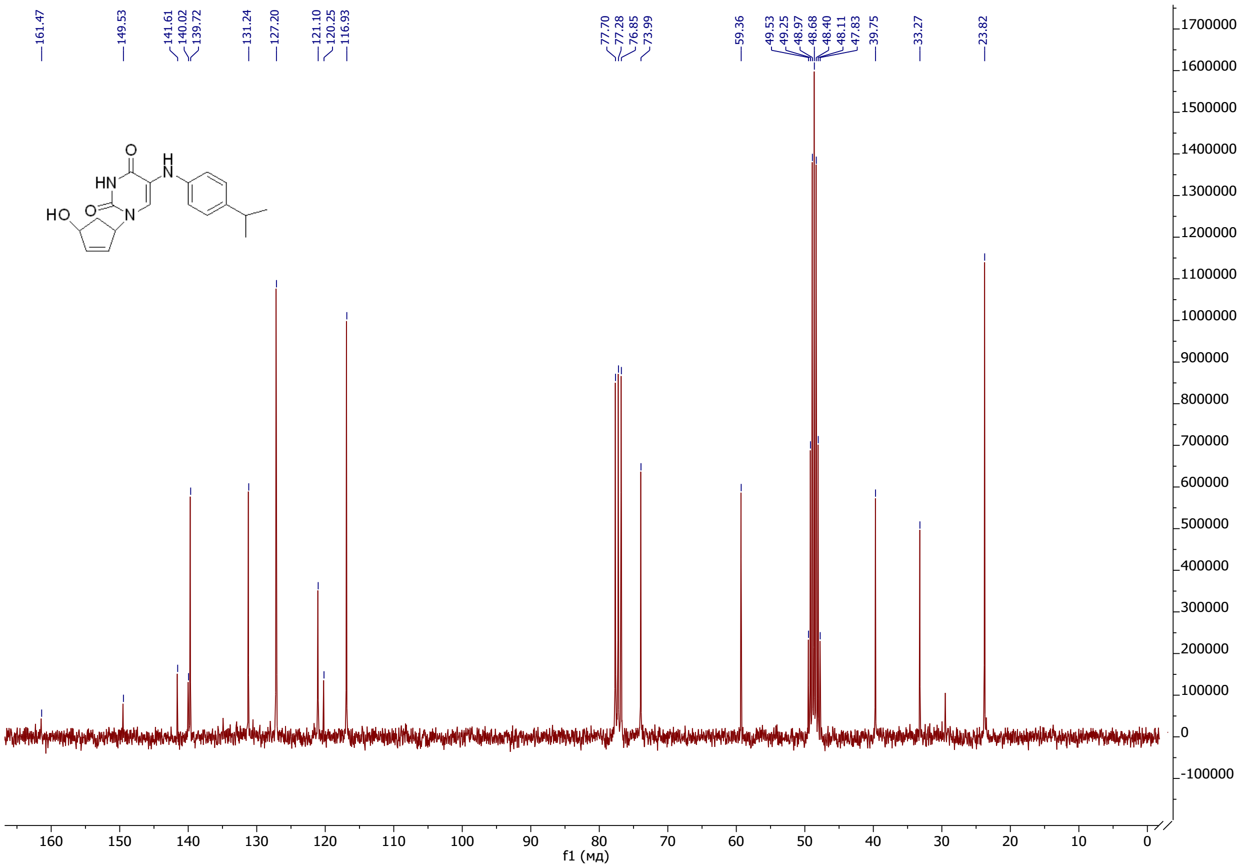

Figure S52 ^{13}C NMR spectrum of compound **3g** in $CDCl_3$:CD_3OD at 100 MHz.

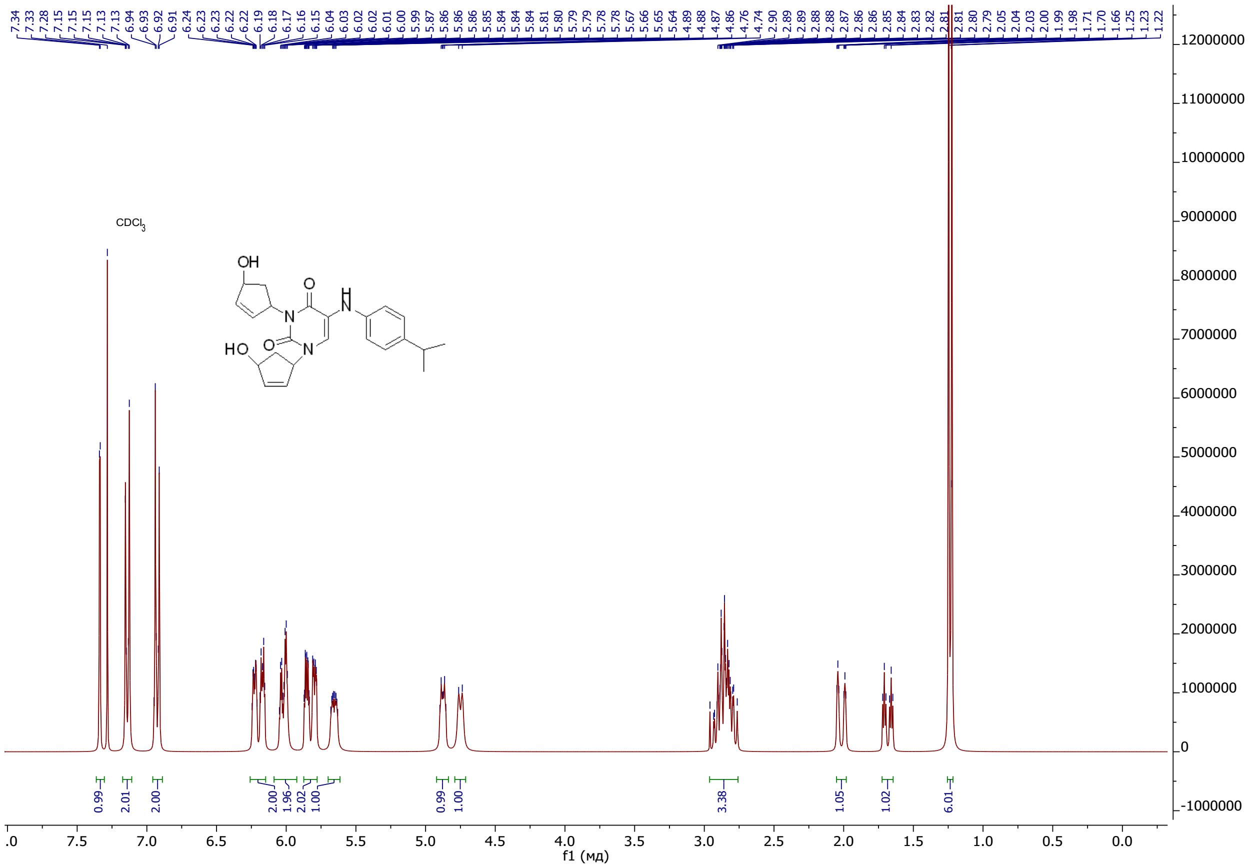

Figure S53 ^{1}H NMR spectrum of compound **4g** in $CDCl_3$ at 400 MHz.

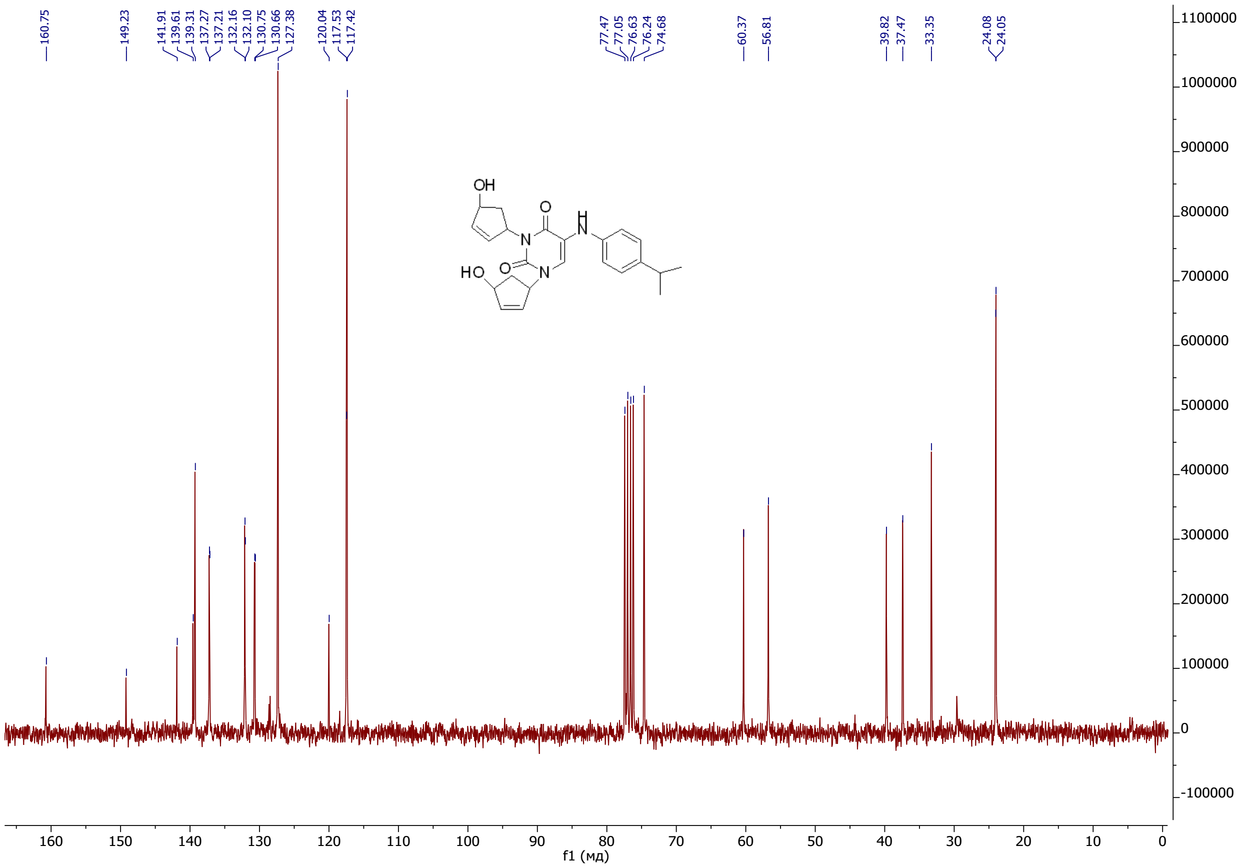

Figure S54 ^{13}C NMR spectrum of compound **4g** in $CDCl_3$ at 100 MHz.

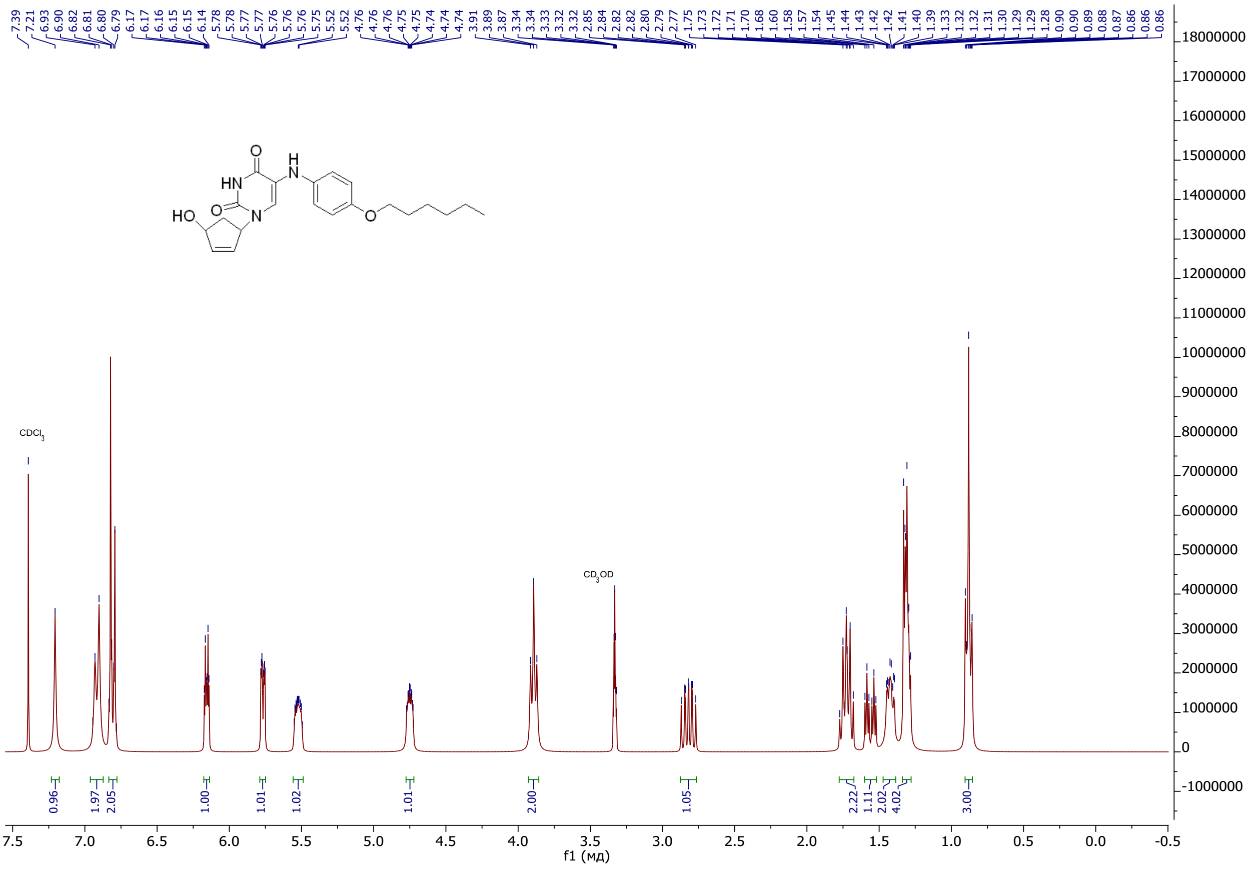

Figure S55 ^{1}H NMR spectrum of compound **3h** in $CDCl_3$:CD_3OD at 400 MHz.

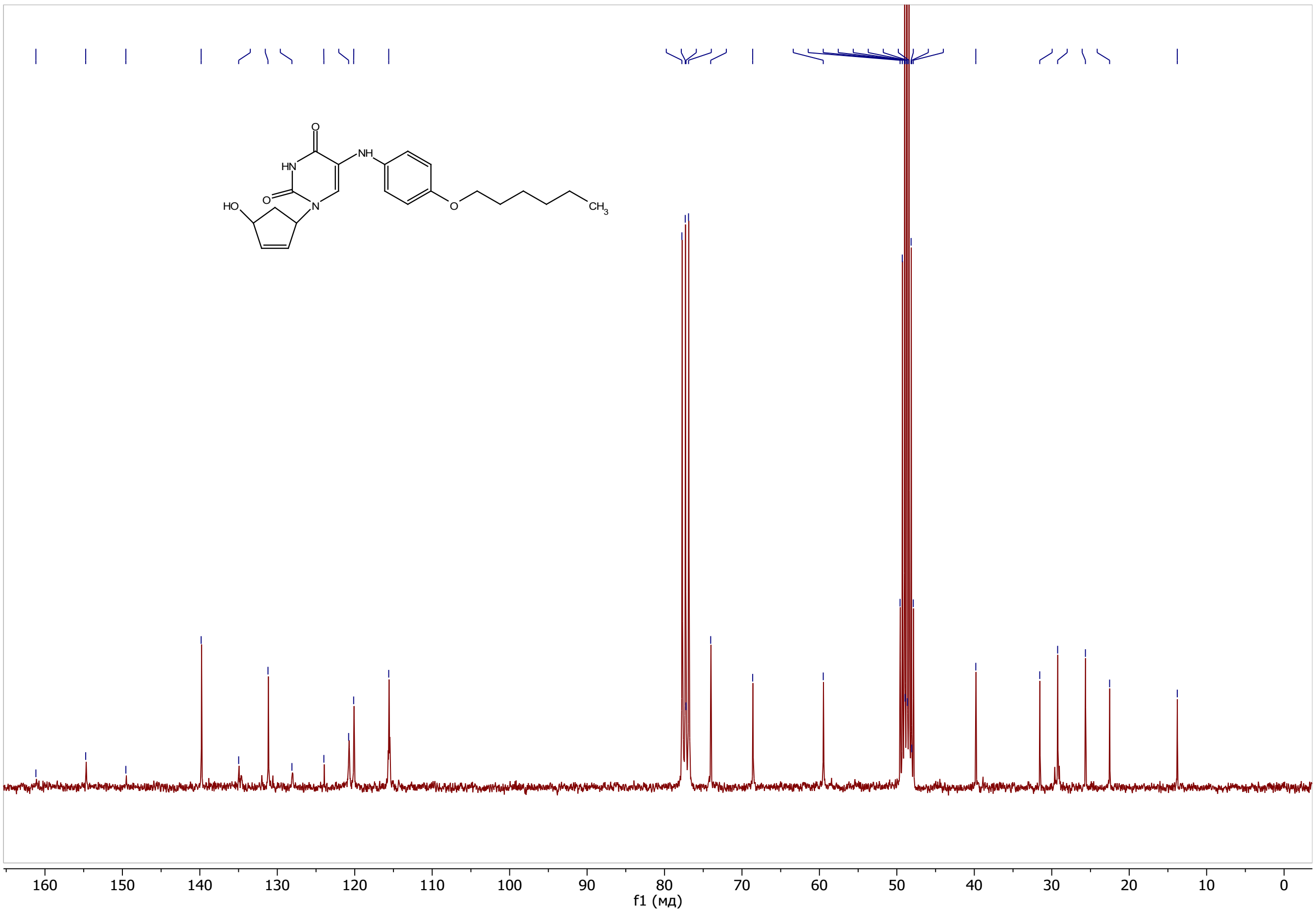

Figure S56 ^{13}C NMR spectrum of compound **3h** in $CDCl_3$:CD_3OD at 100 MHz.

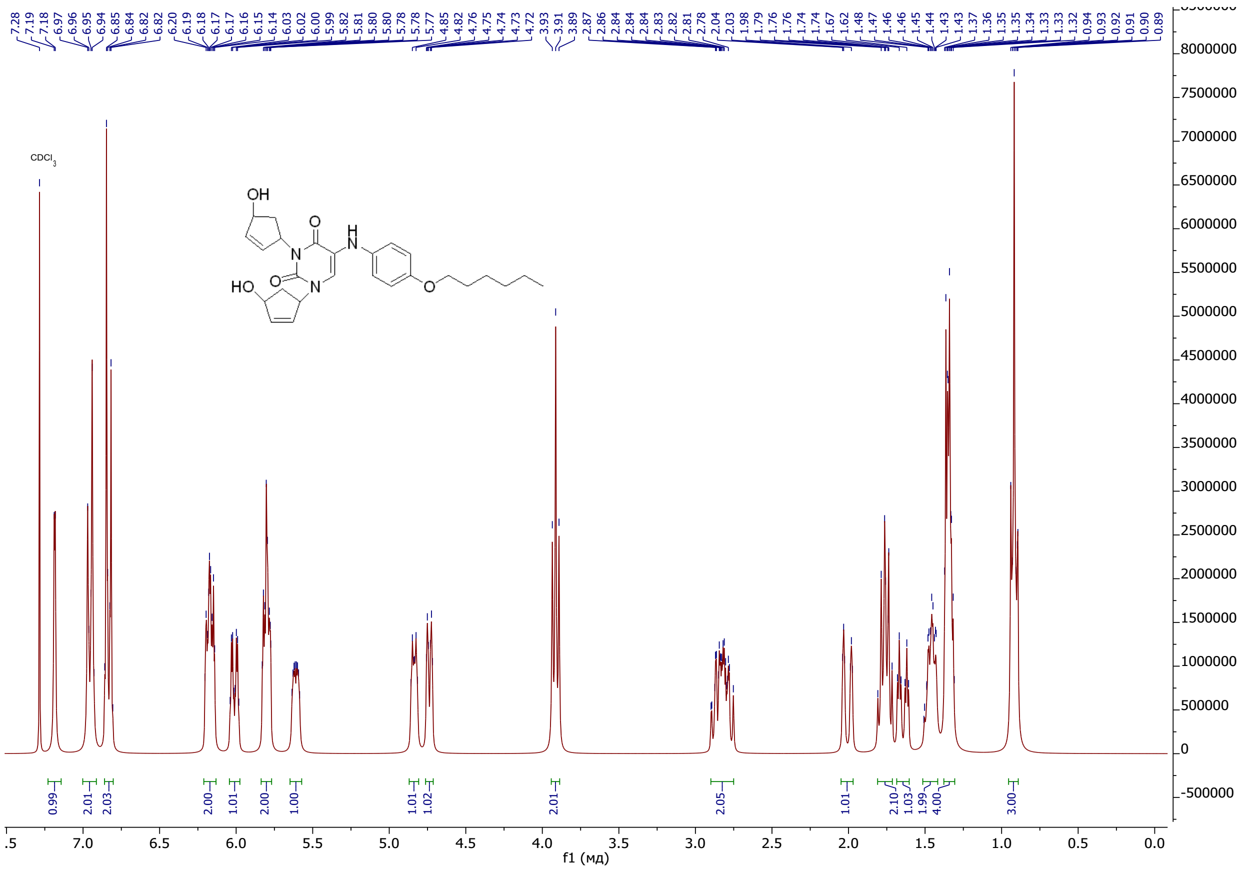

Figure S57 ^{1}H NMR spectrum of compound **4h** in $CDCl_3$ at 400 MHz.

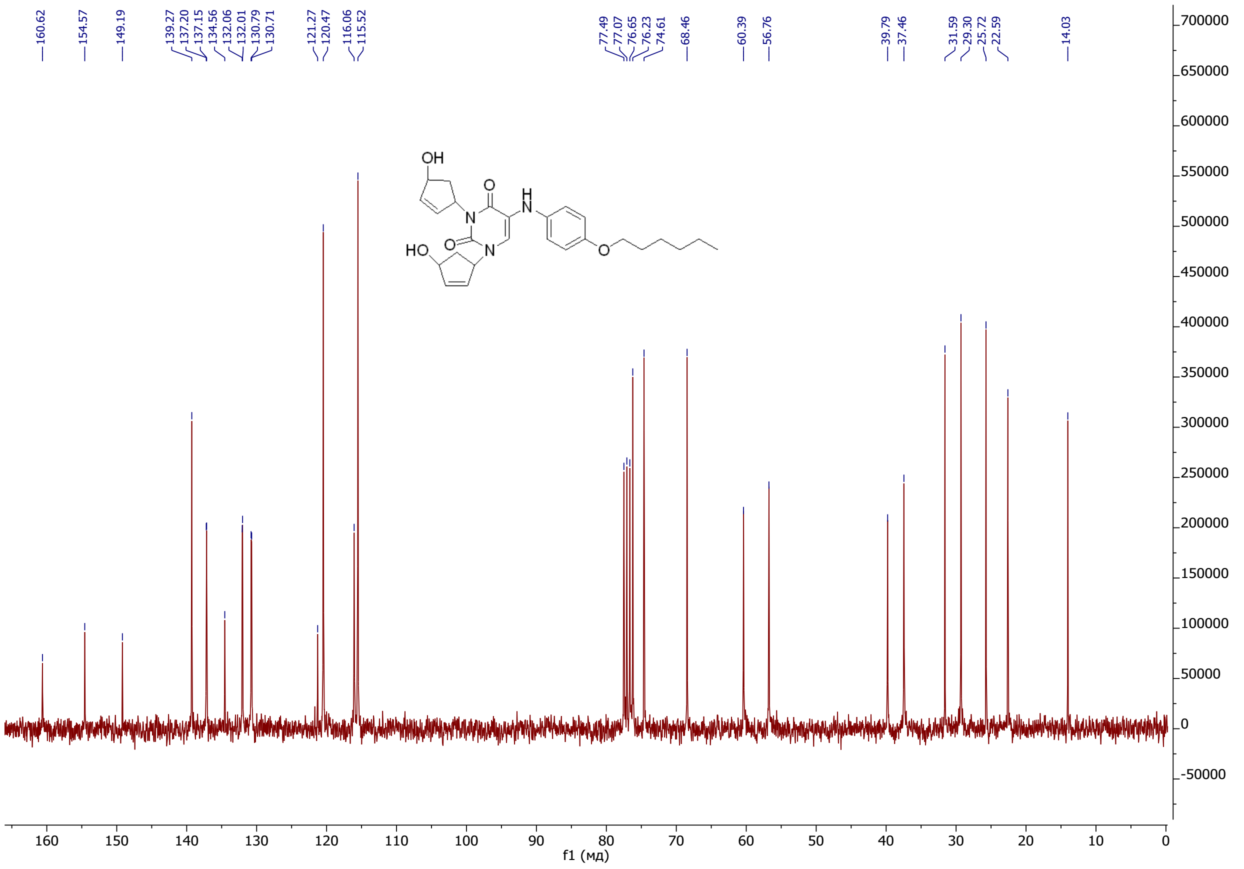

Figure S58 ^{13}C NMR spectrum of compound **4h** in $CDCl_3$ at 100 MHz.

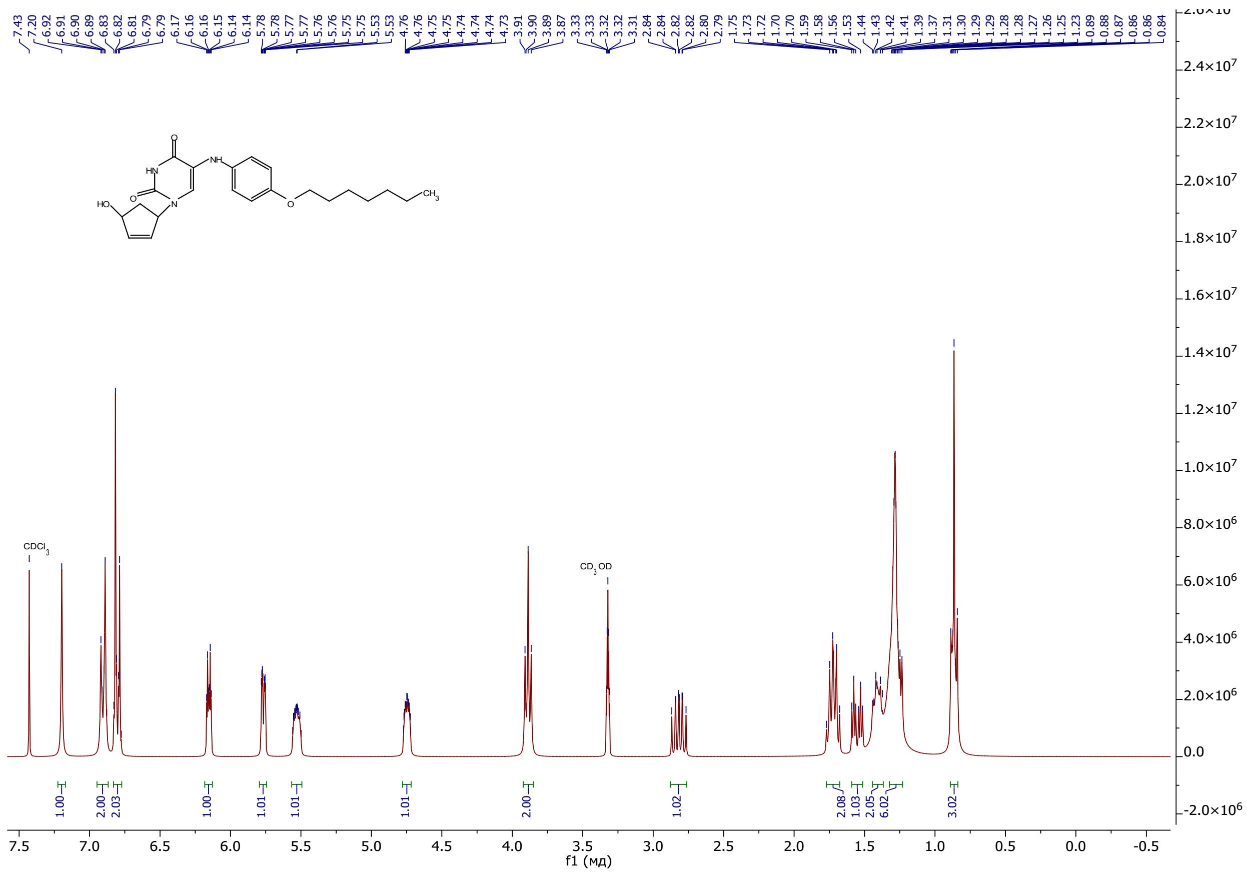

Figure S59 ^{1}H NMR spectrum of compound **3i** in $CDCl_3$:CD_3OD at 400 MHz.

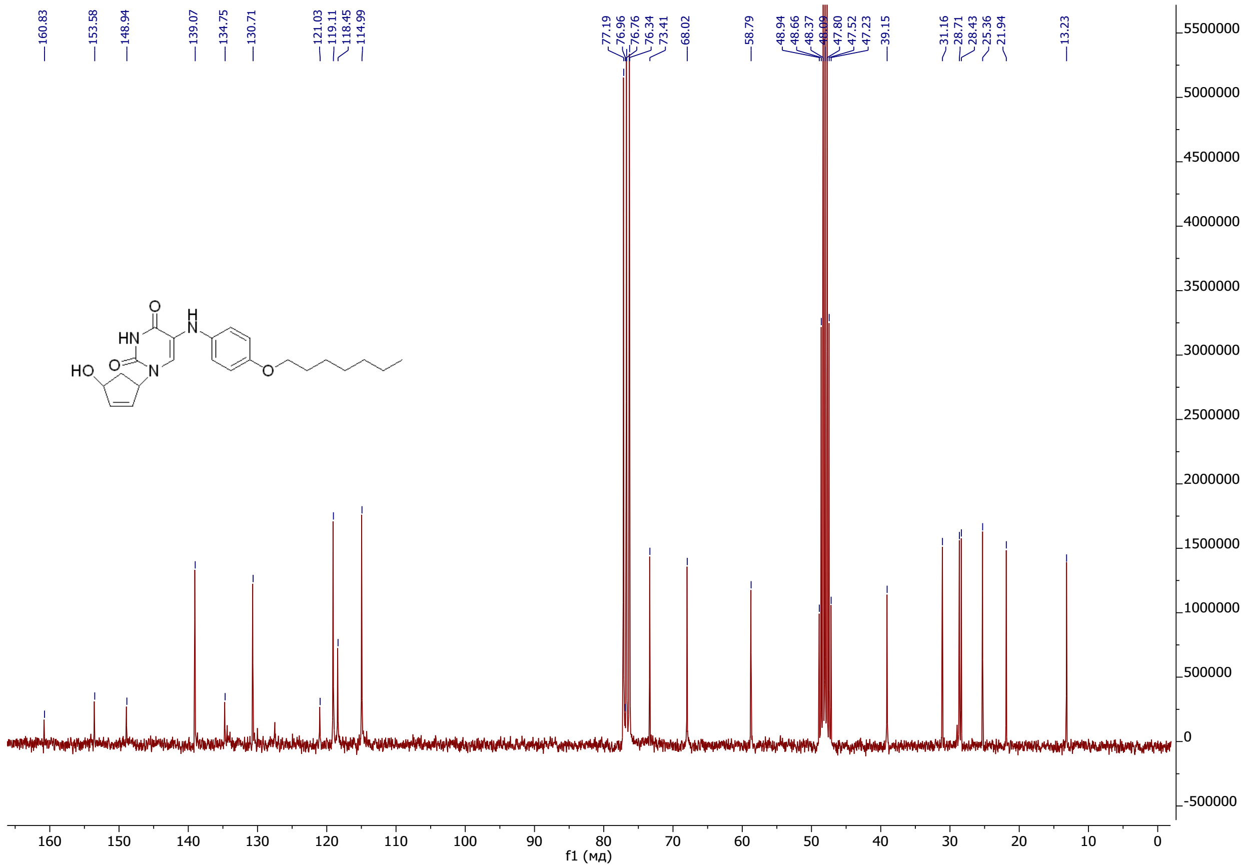

Figure S60 ^{13}C NMR spectrum of compound **3i** in $CDCl_3$:CD_3OD at 100 MHz.

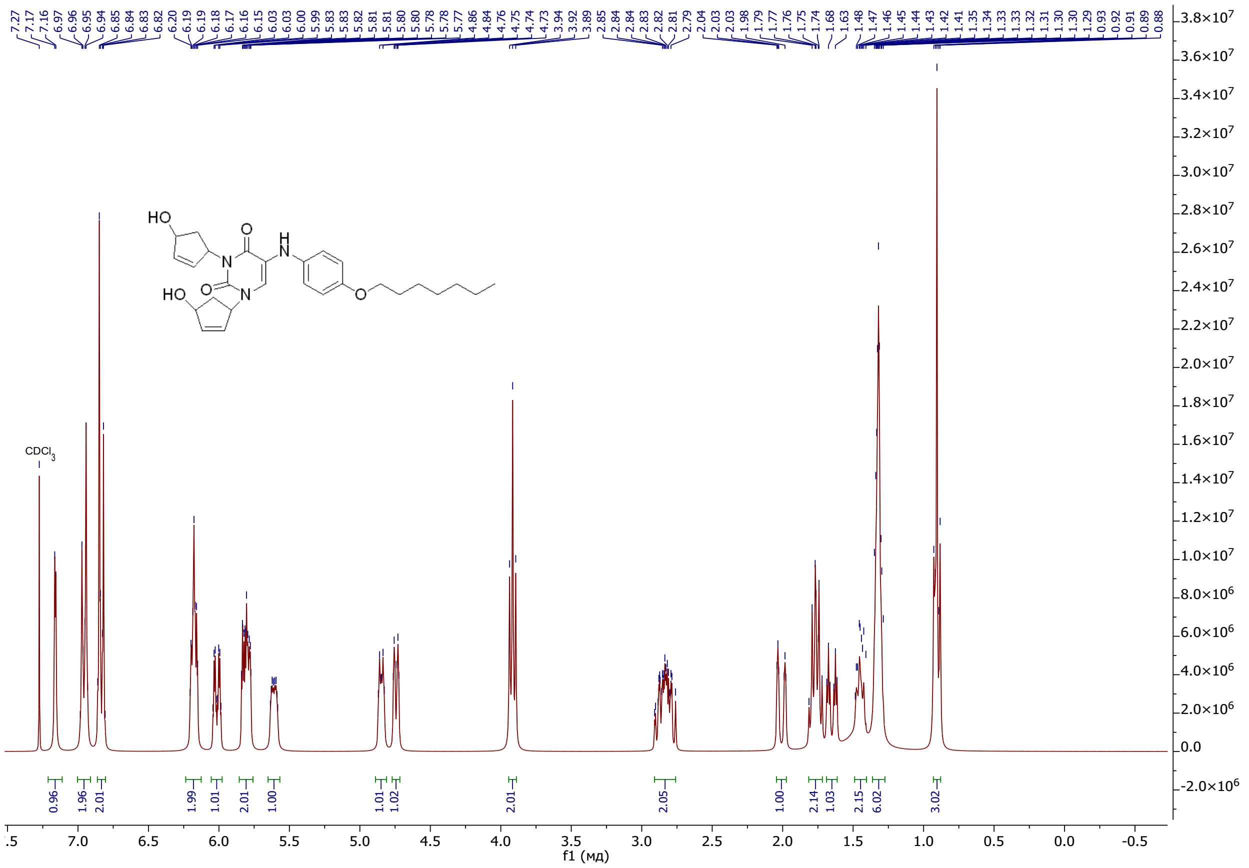

Figure S61 ^{1}H NMR spectrum of compound **4i** in $CDCl_3$ at 400 MHz.

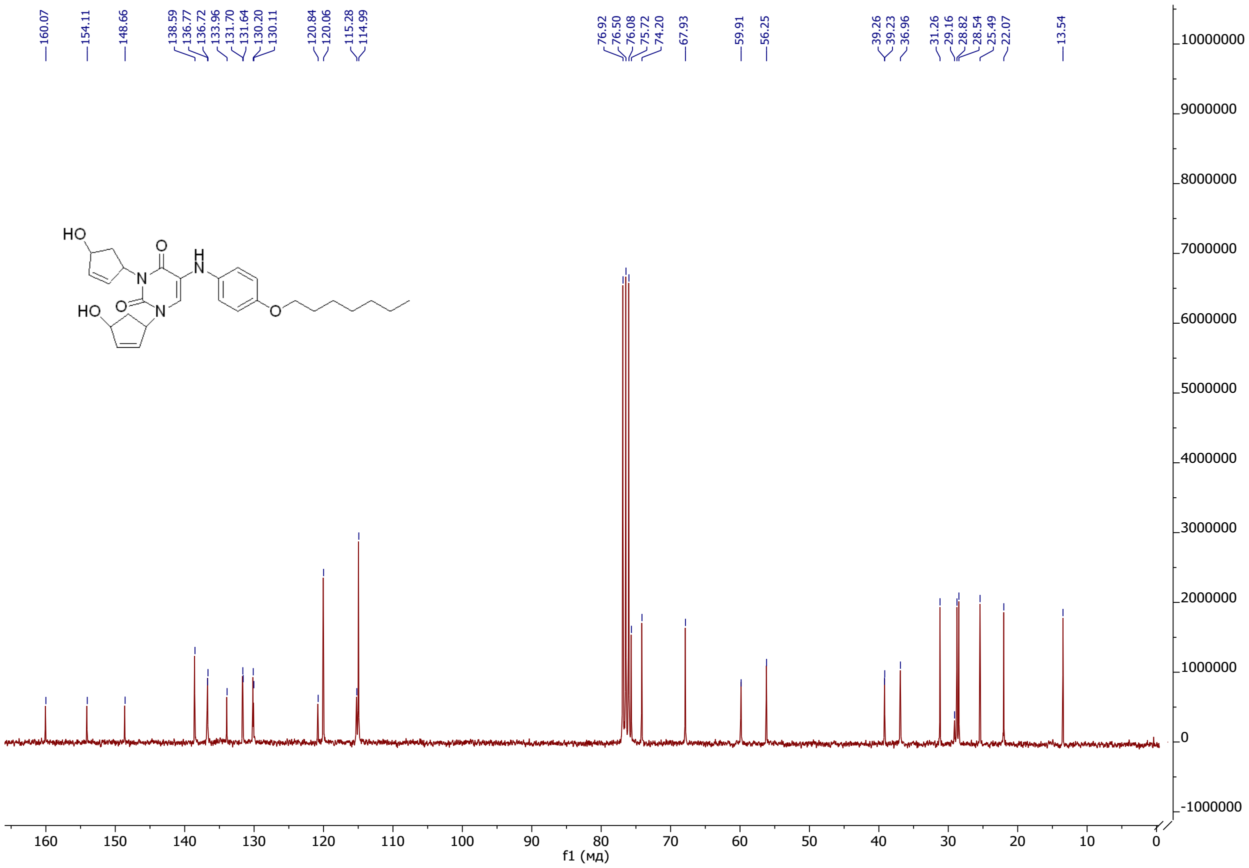

Figure S62 ^{13}C NMR spectrum of compound **4i** in $CDCl_3$ at 100 MHz.

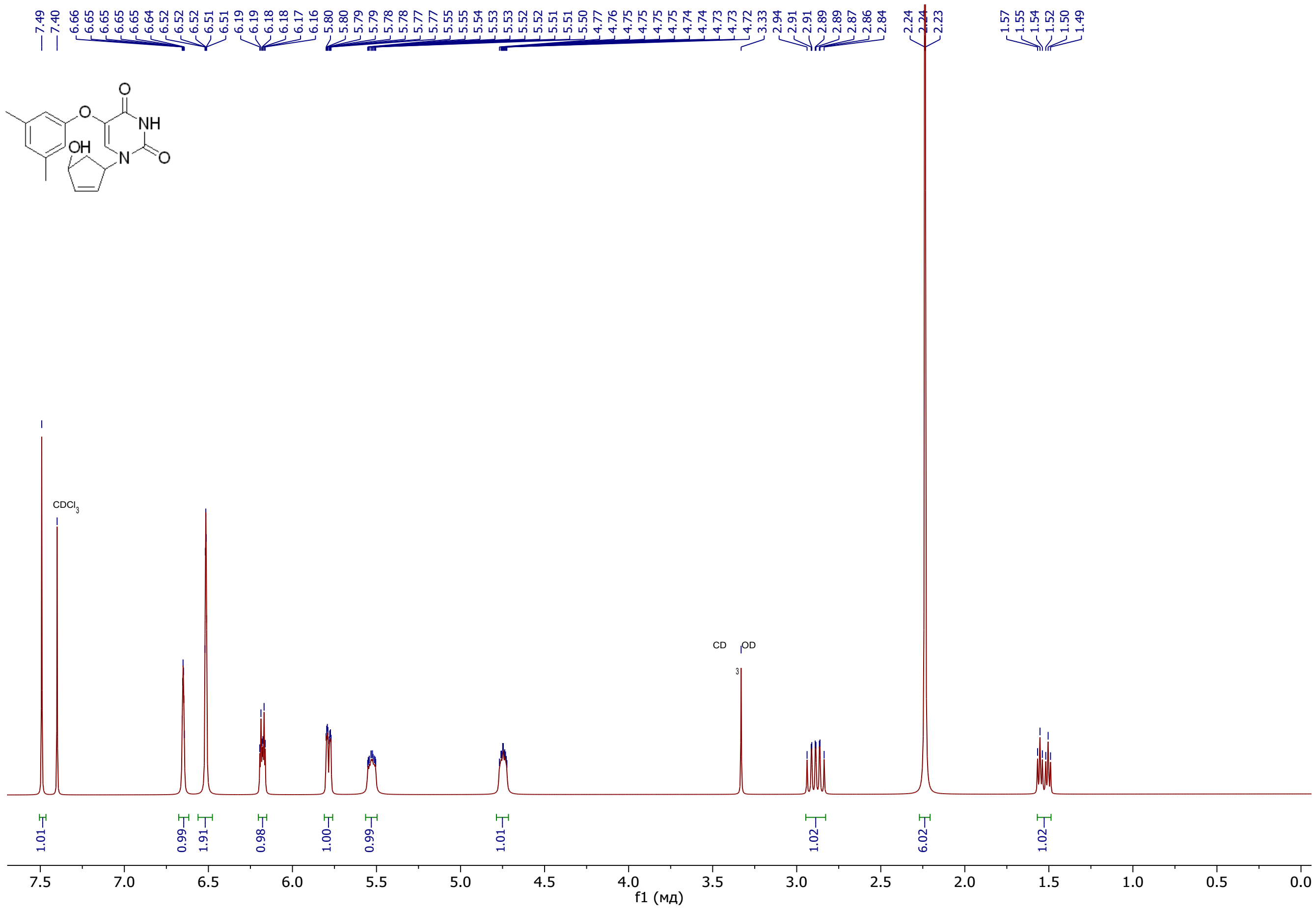

Figure S63 ^{1}H NMR spectrum of compound **10a** in $CDCl_3$:CD_3OD at 400 MHz.

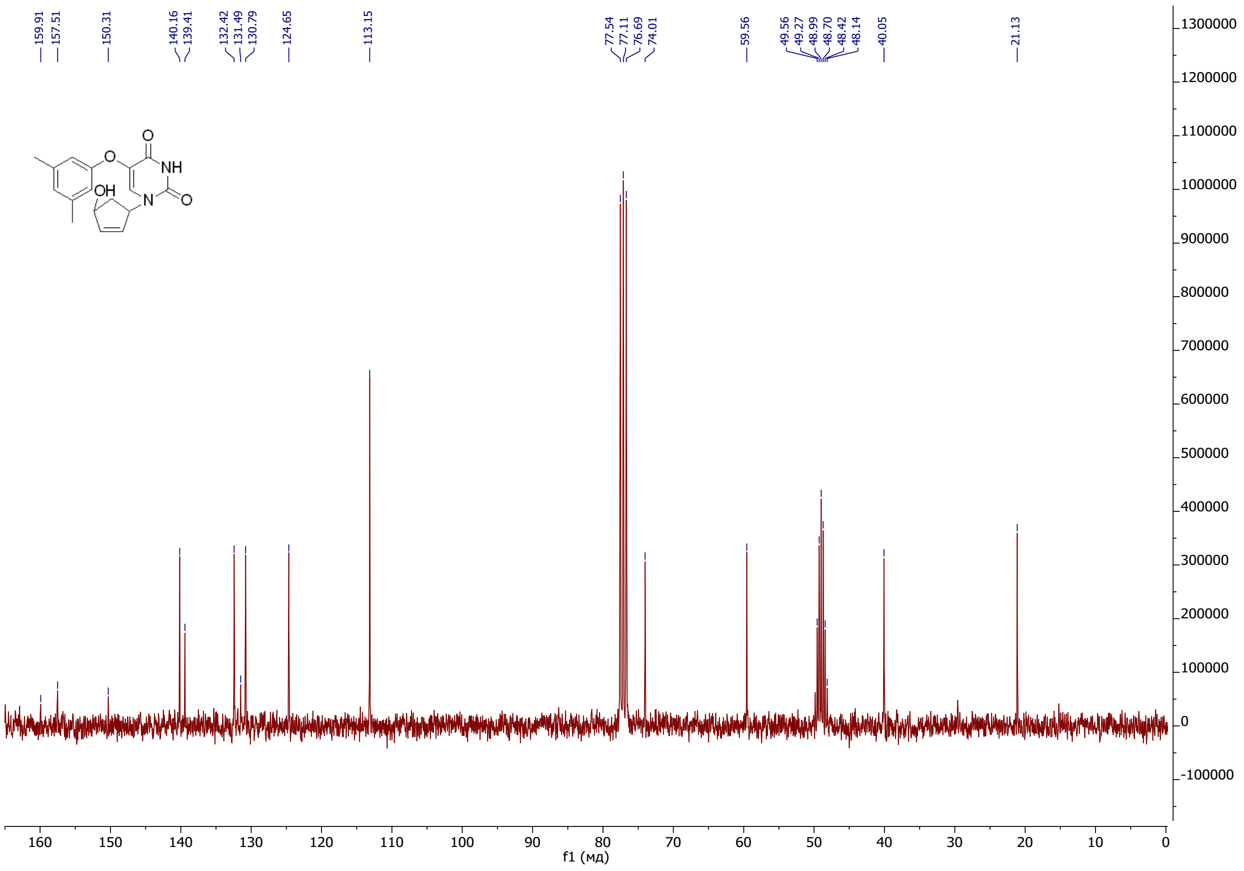

Figure S64 ^{13}C NMR spectrum of compound **10a** in $CDCl_3$:CD_3OD at 100 MHz.

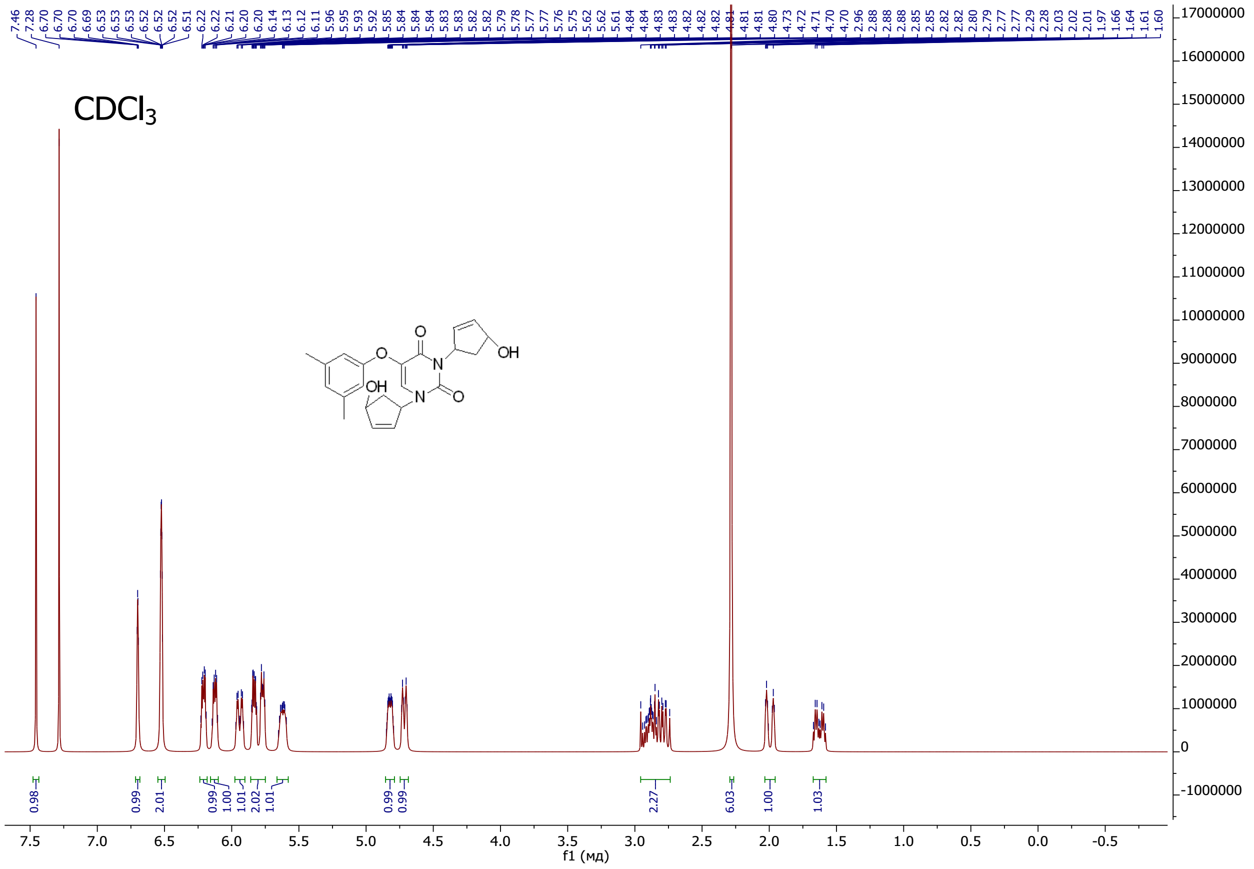

Figure S65 ^{1}H NMR spectrum of compound **11a** in $CDCl_3$ at 400 MHz.

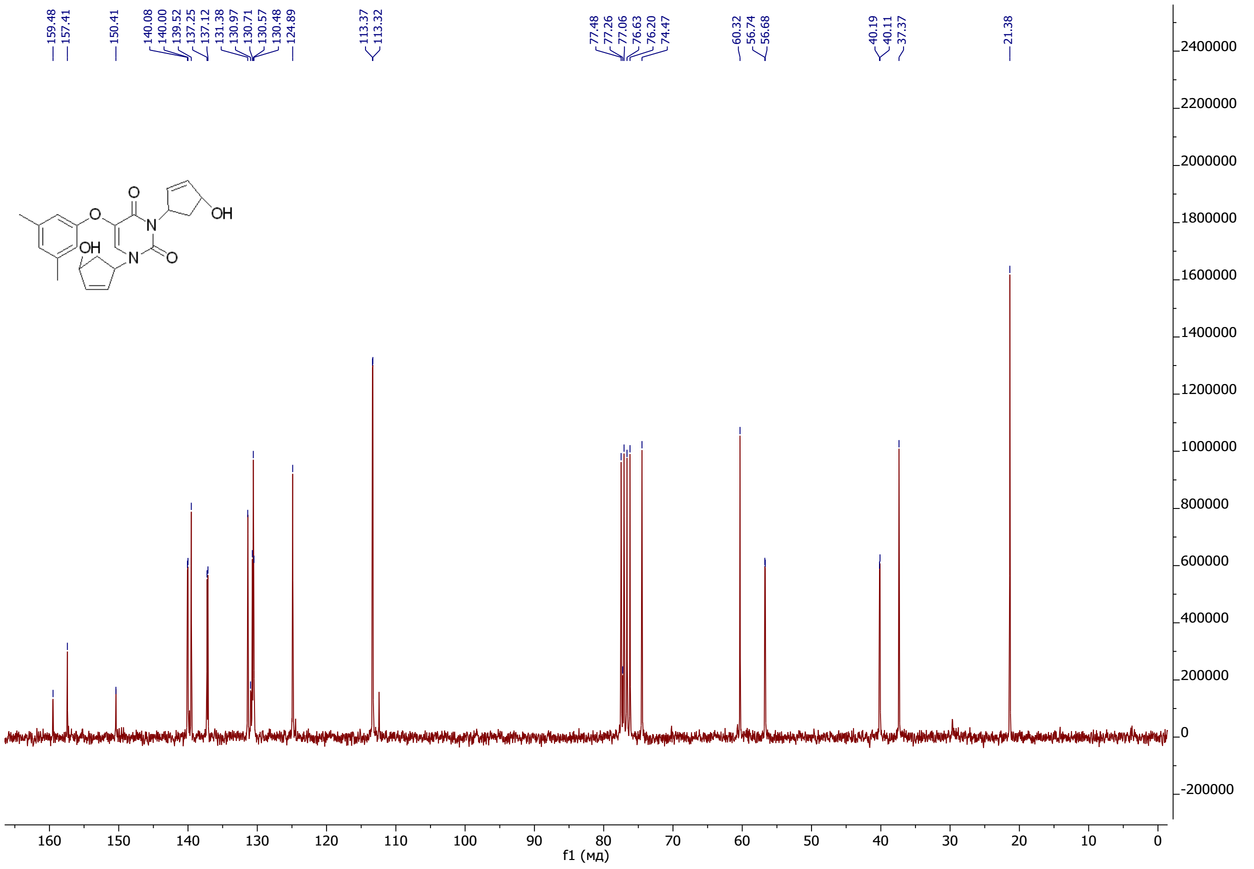

Figure S66 ^{13}C NMR spectrum of compound **11a** in $CDCl_3$ at 100 MHz.

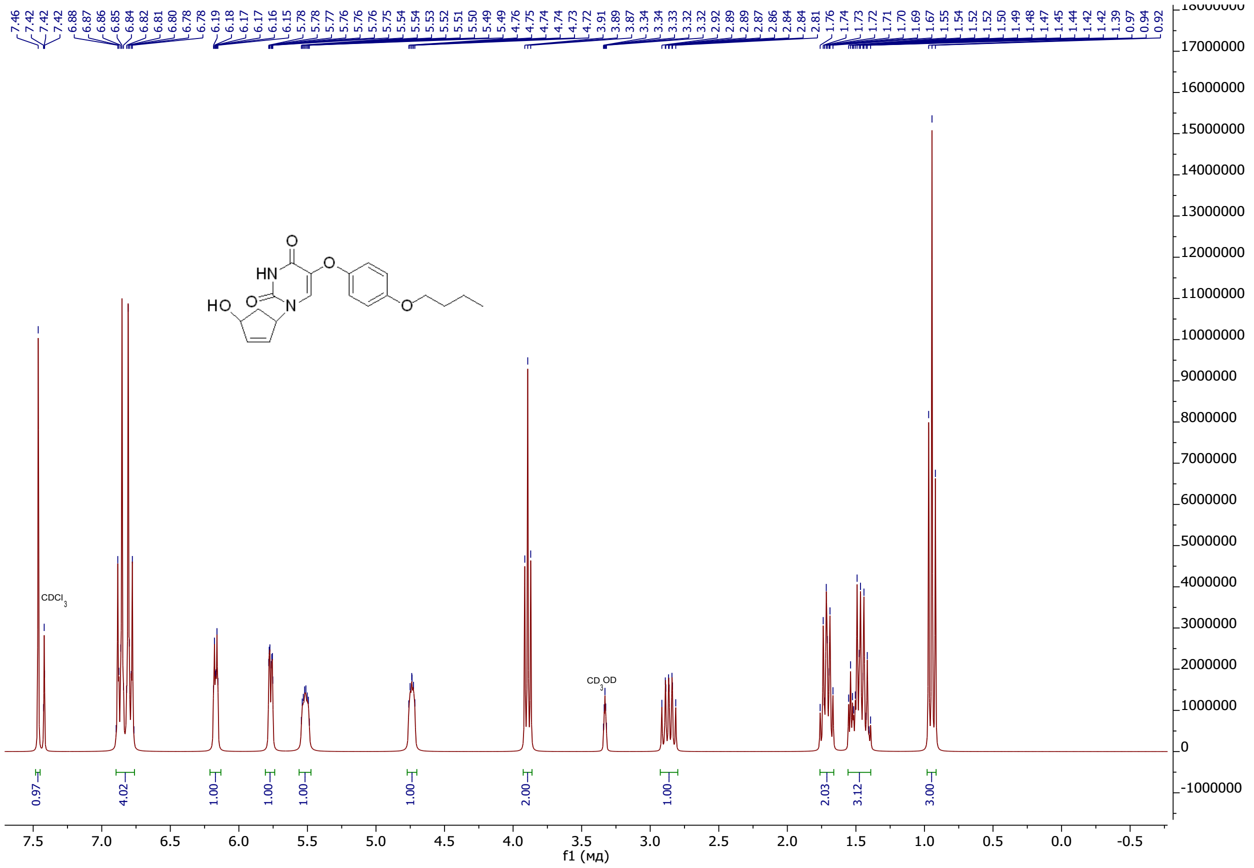

Figure S67 ^{1}H NMR spectrum of compound **10b** in $CDCl_3$:CD_3OD at 400 MHz.

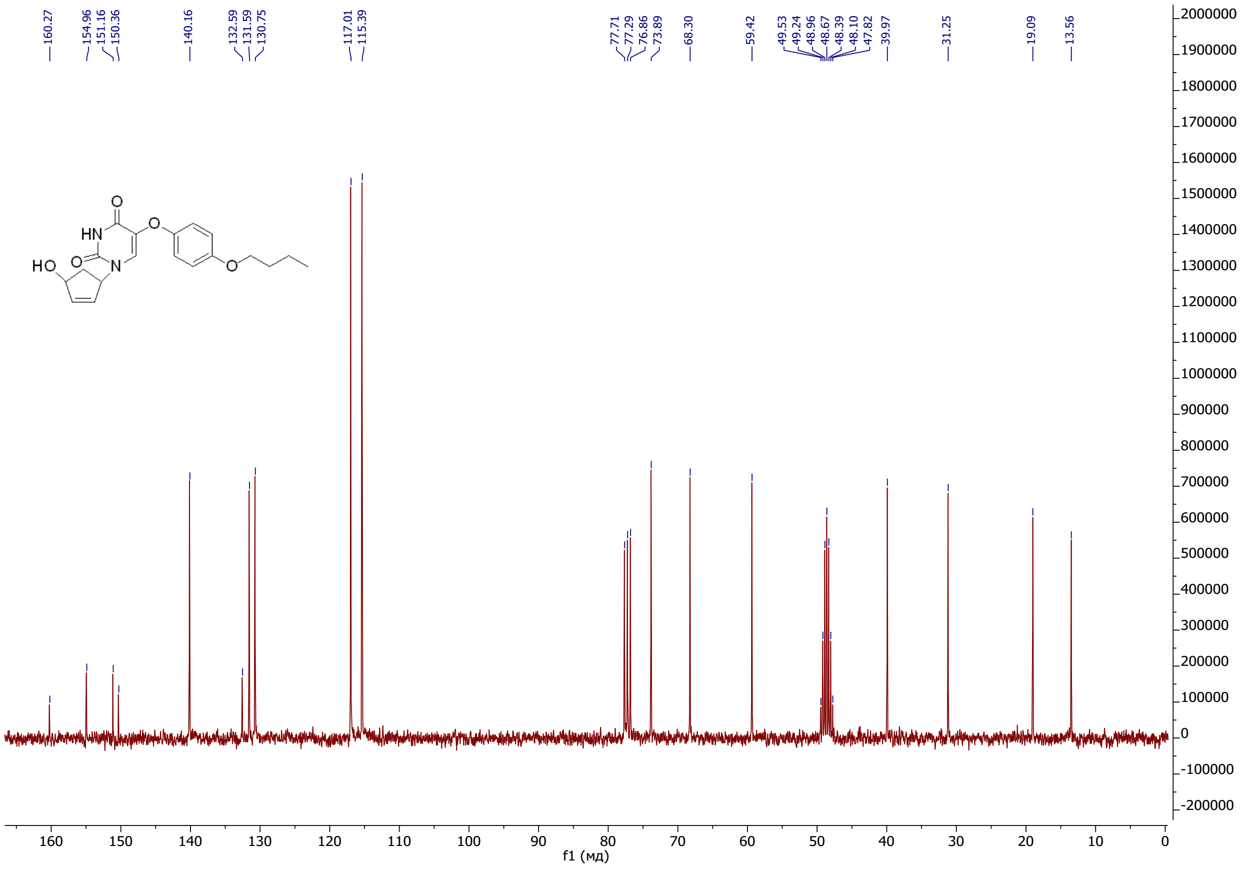

Figure S68 ^{13}C NMR spectrum of compound **10b** in $CDCl_3$:CD_3OD at 100 MHz.

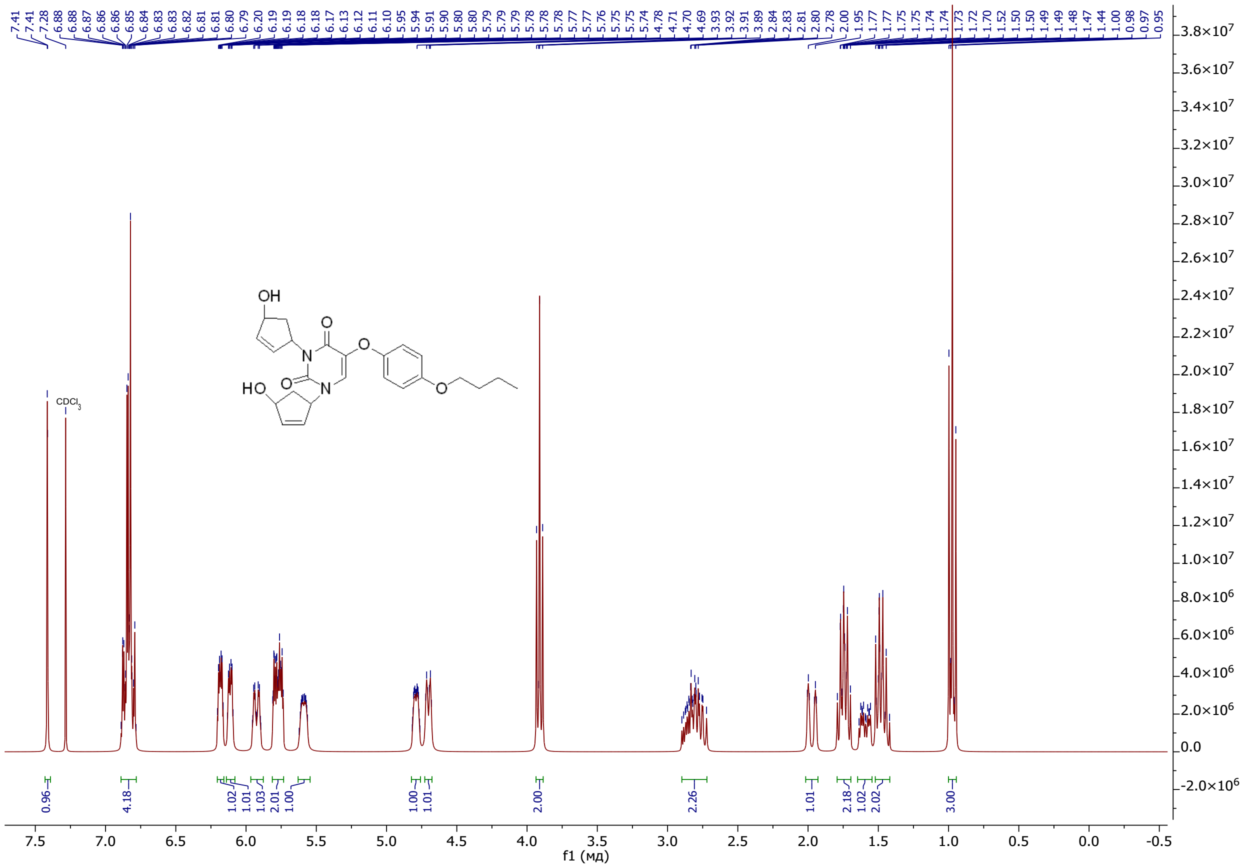

Figure S69 ^{1}H NMR spectrum of compound **11b** in $CDCl_3$ at 400 MHz.

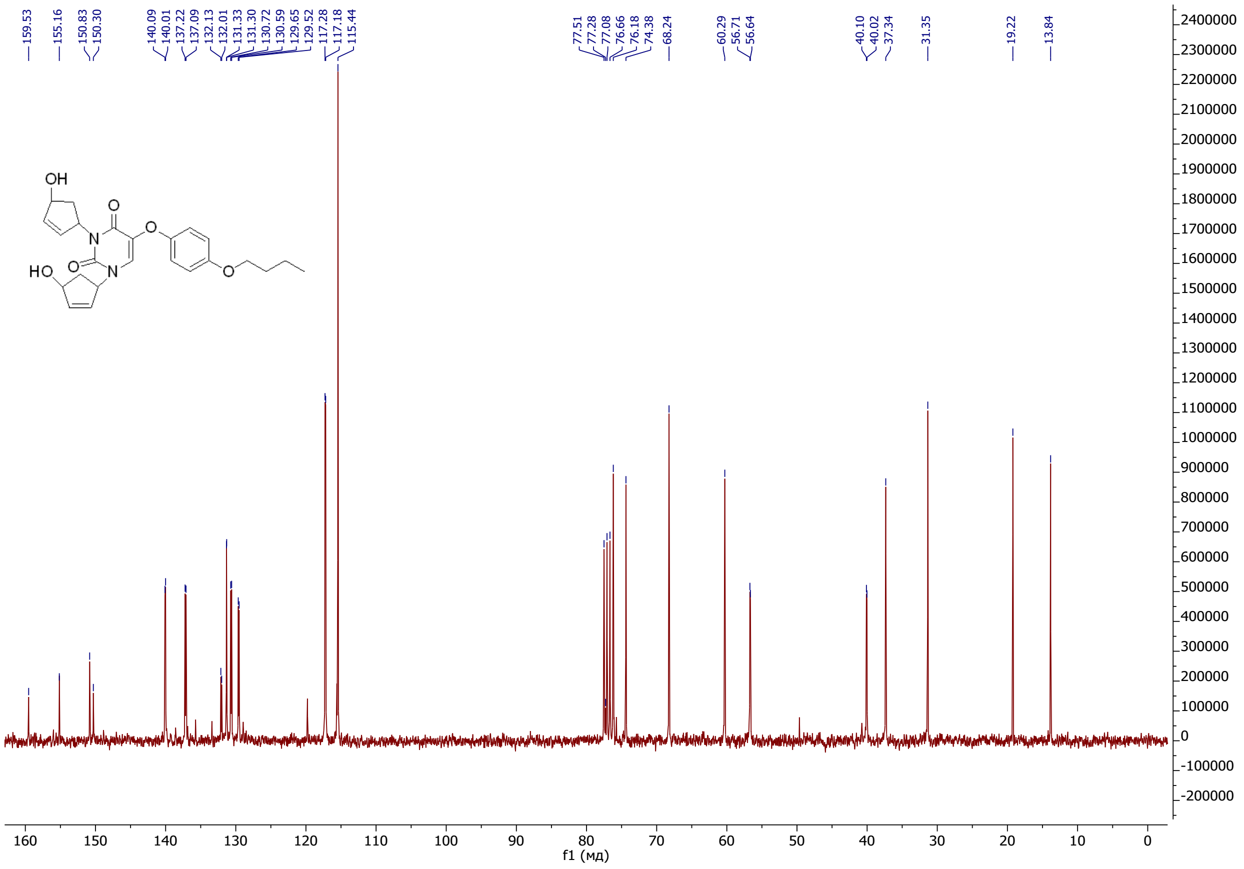

Figure S70 ^{13}C NMR spectrum of compound **11b** in $CDCl_3$ at 100 MHz.

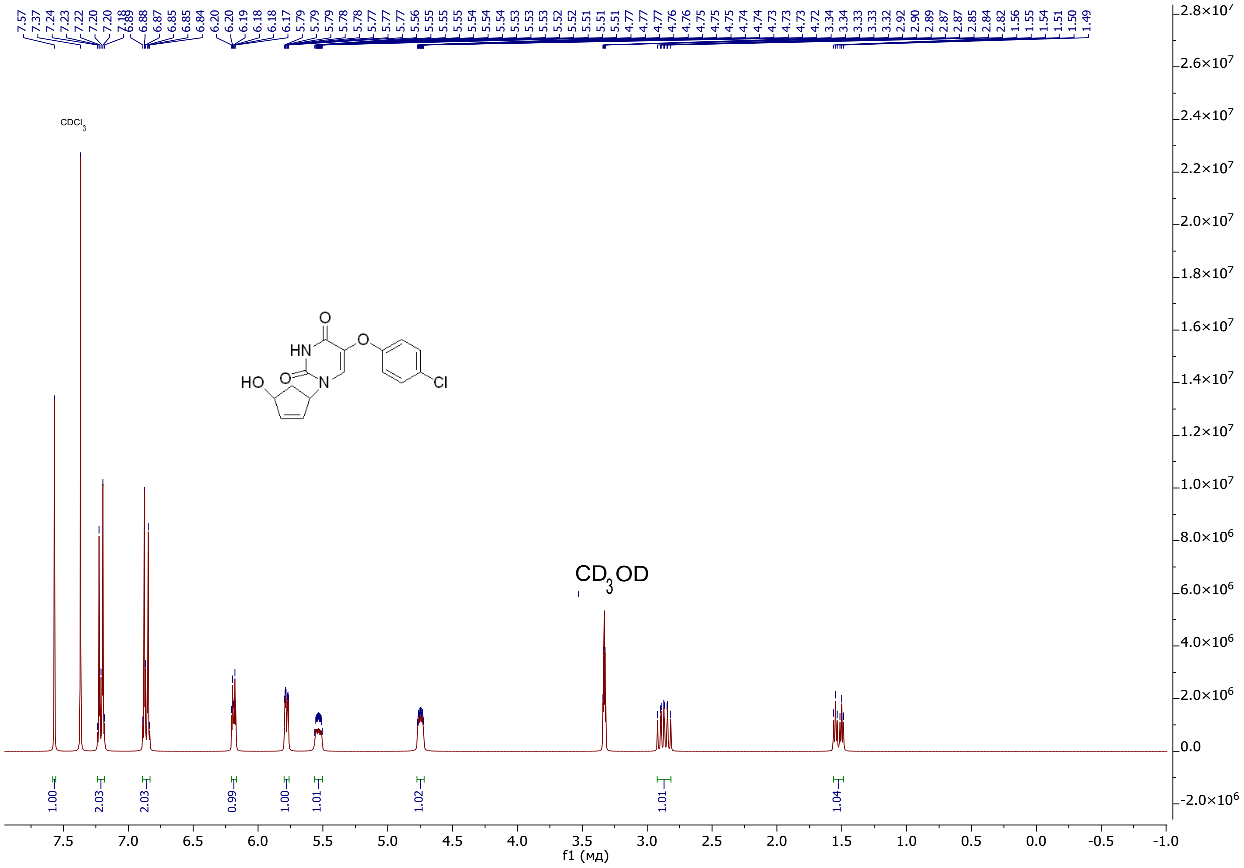

Figure S71 ^{1}H NMR spectrum of compound **10c** in $CDCl_3$:CD_3OD at 400 MHz.

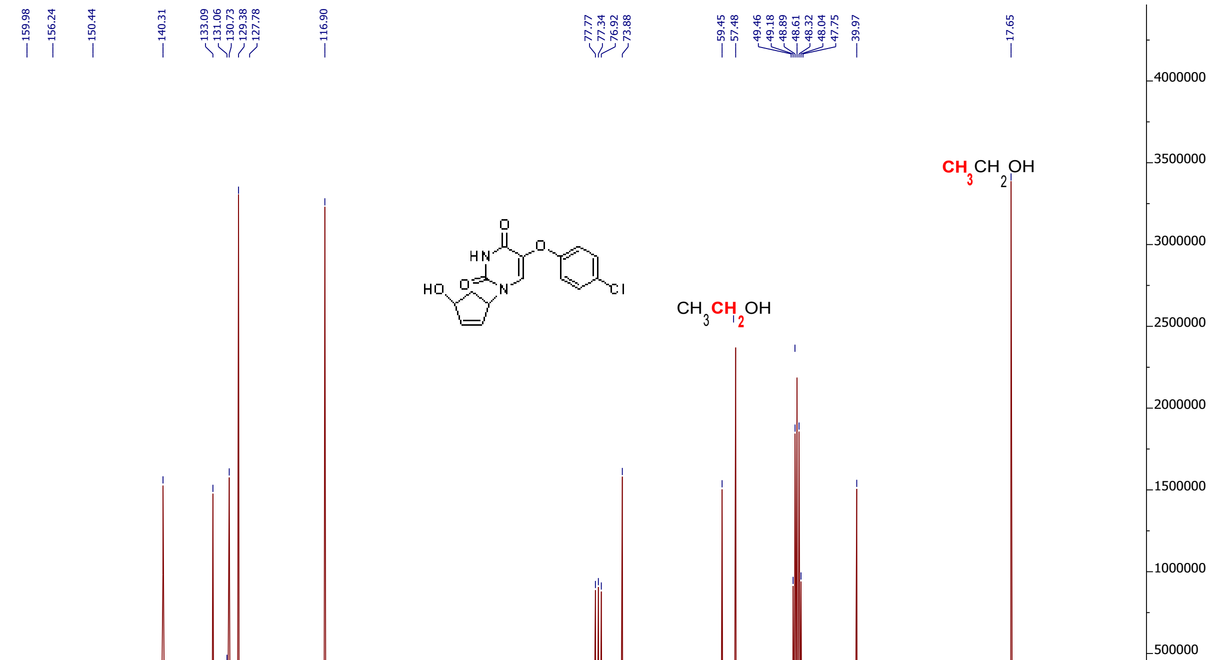

Figure S72 ^{13}C NMR spectrum of compound **10c** in $CDCl_3$:CD_3OD at 100 MHz.

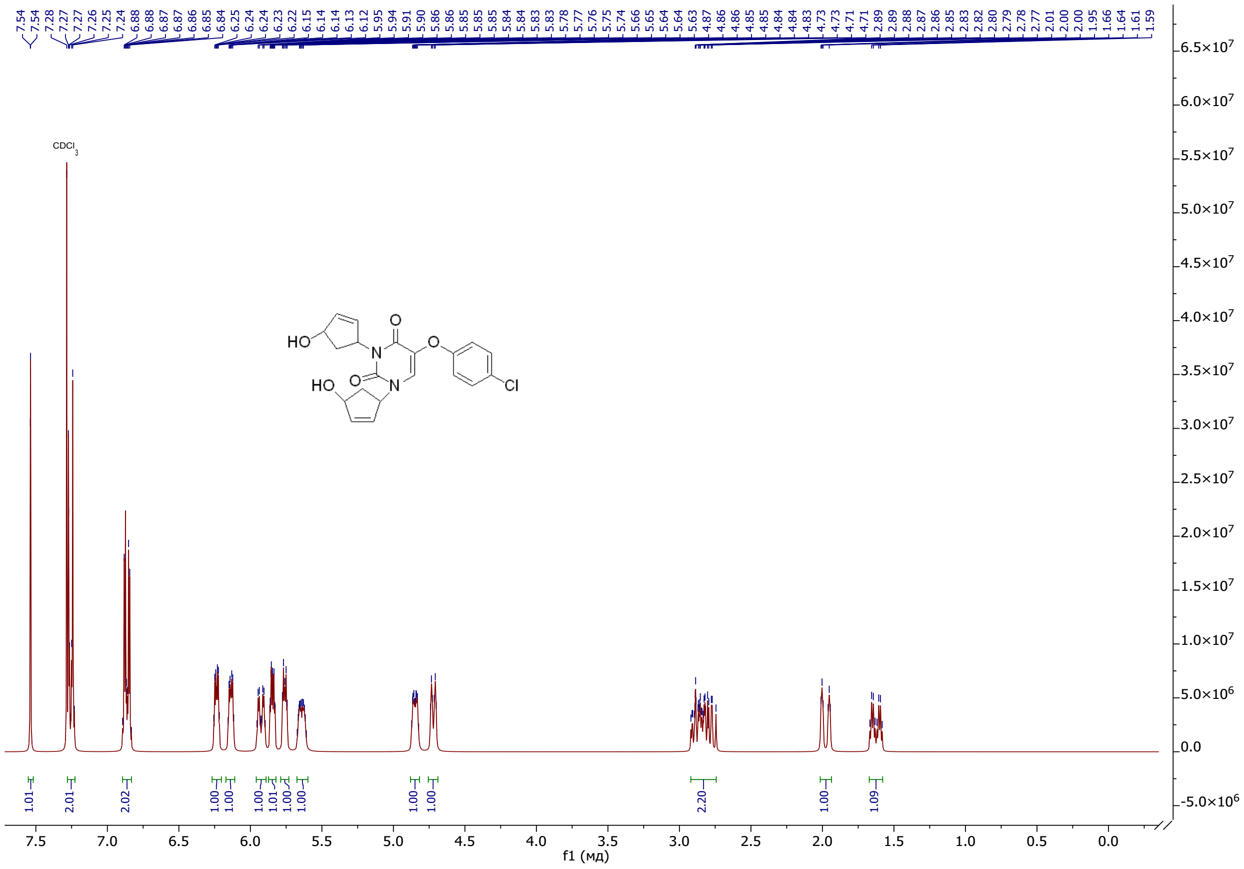

Figure S73 ^{1}H NMR spectrum of compound **11c** in $CDCl_3$ at 400 MHz.

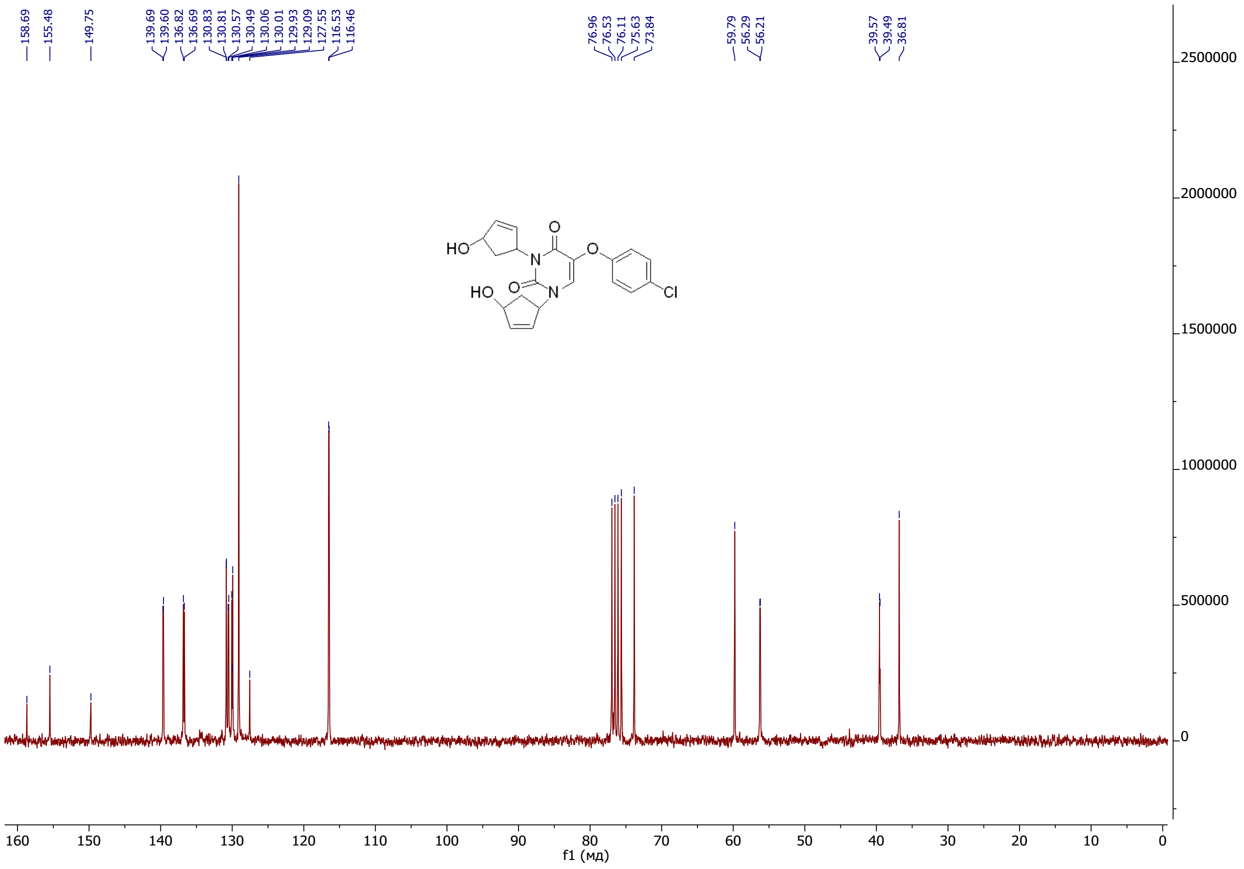

Figure S74 ^{13}C NMR spectrum of compound **11c** in $CDCl_3$ at 100 MHz.

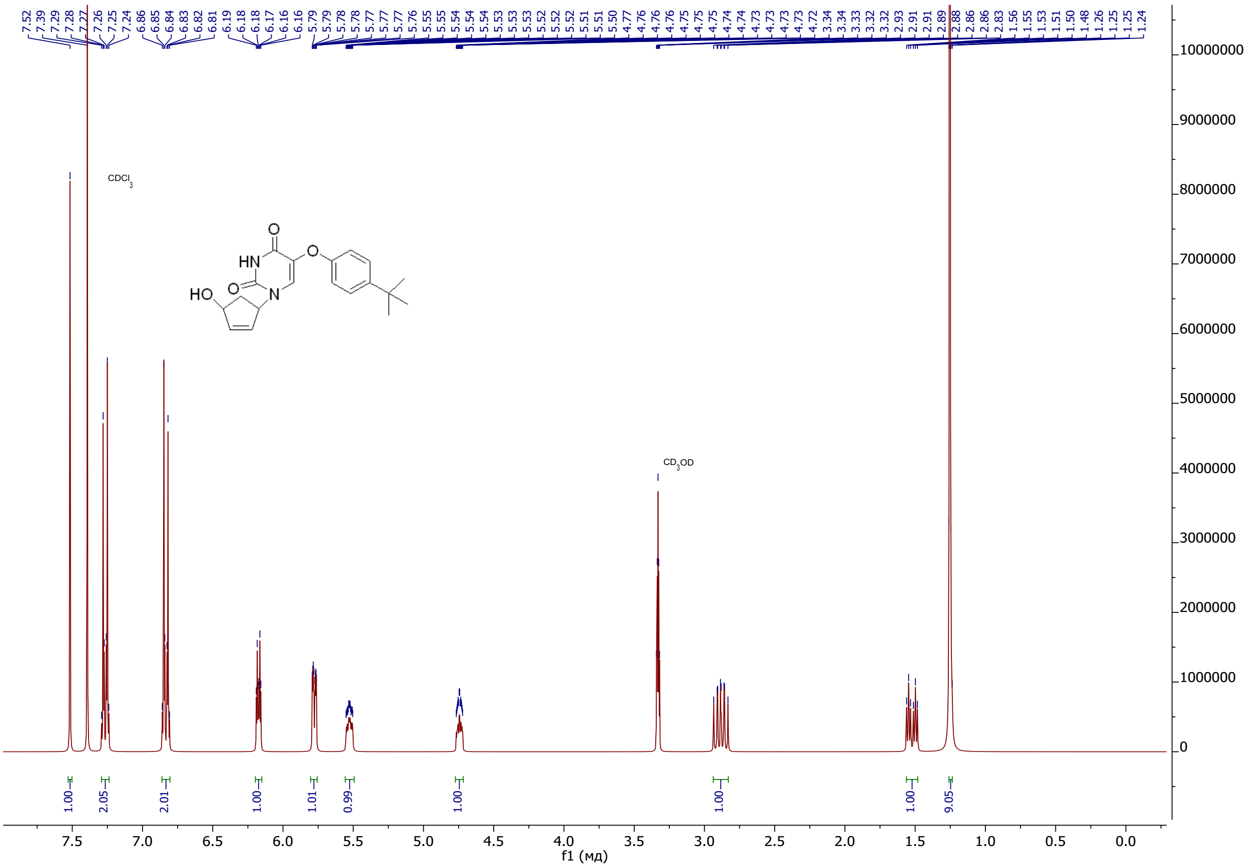

Figure S75 ^{1}H NMR spectrum of compound **10d** in $CDCl_3$:CD_3OD at 400 MHz.

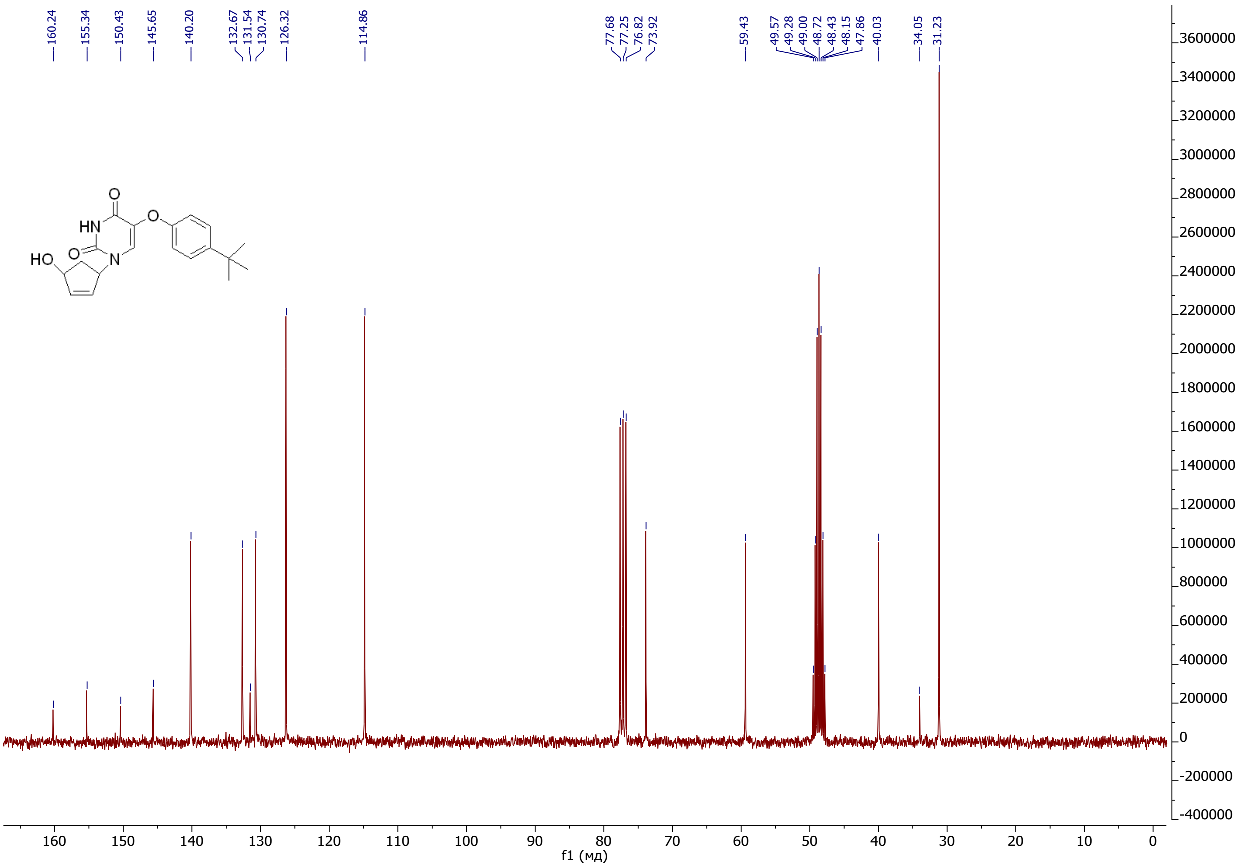

Figure S76 ^{13}C NMR spectrum of compound **10d** in $CDCl_3$:CD_3OD at 100 MHz.

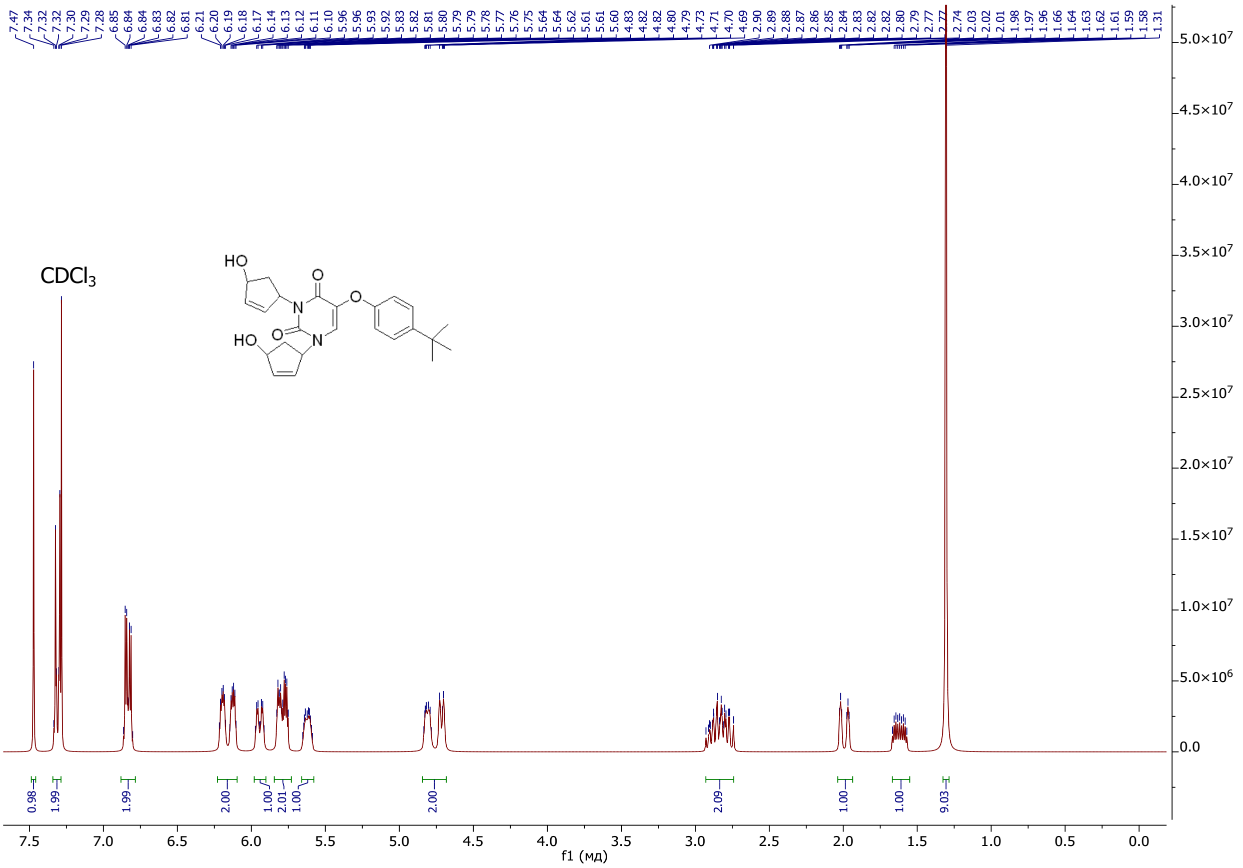

Figure S77 ^{1}H NMR spectrum of compound **11d** in $CDCl_3$ at 400 MHz.

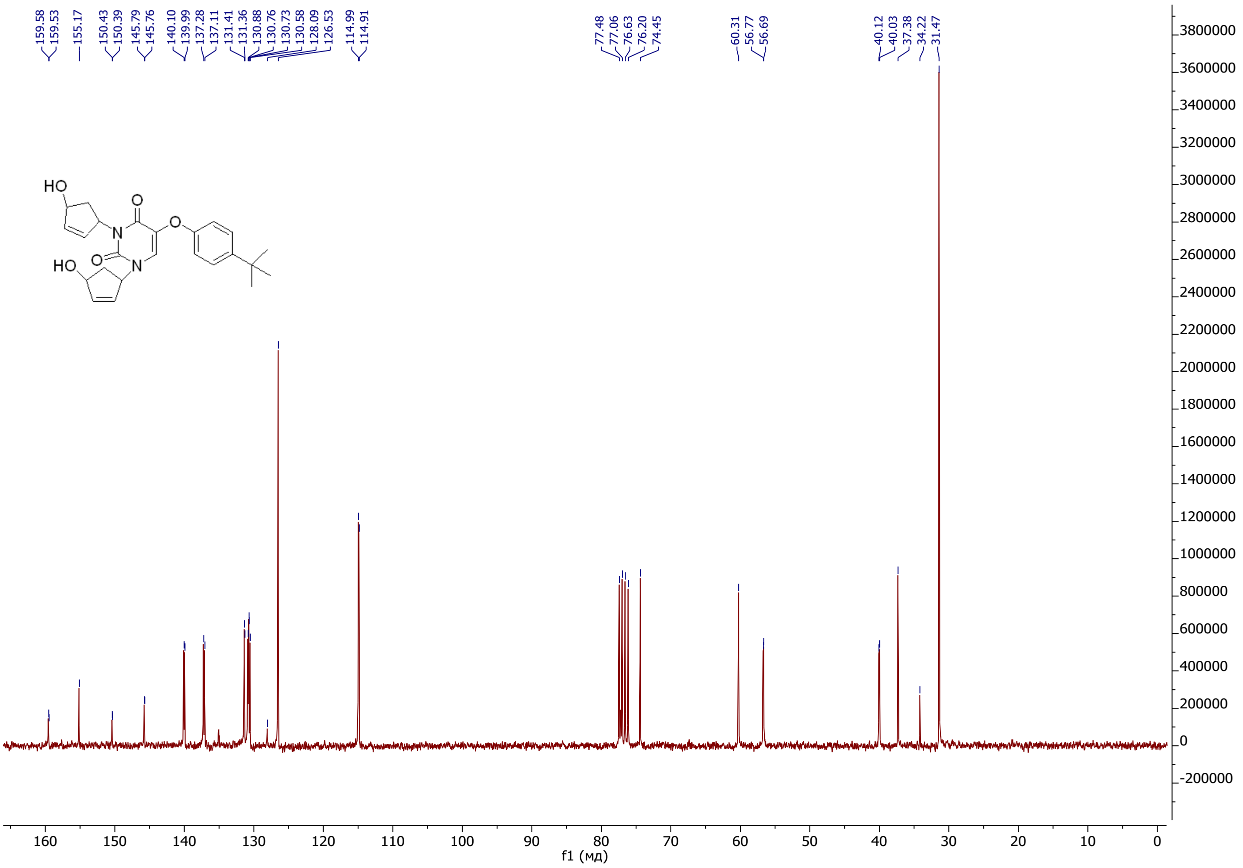

Figure S78 ^{13}C NMR spectrum of compound **11d** in $CDCl_3$ at 100 MHz.
